# A genome-wide association study of blood cell morphology identifies cellular proteins implicated in disease aetiology

**DOI:** 10.1038/s41467-023-40679-y

**Published:** 2023-08-18

**Authors:** Parsa Akbari, Dragana Vuckovic, Luca Stefanucci, Tao Jiang, Kousik Kundu, Roman Kreuzhuber, Erik L. Bao, Janine H. Collins, Kate Downes, Luigi Grassi, Jose A. Guerrero, Stephen Kaptoge, Julian C. Knight, Stuart Meacham, Jennifer Sambrook, Denis Seyres, Oliver Stegle, Jeffrey M. Verboon, Klaudia Walter, Nicholas A. Watkins, John Danesh, David J. Roberts, Emanuele Di Angelantonio, Vijay G. Sankaran, Mattia Frontini, Stephen Burgess, Taco Kuijpers, James E. Peters, Adam S. Butterworth, Willem H. Ouwehand, Nicole Soranzo, William J. Astle

**Affiliations:** 1 British Heart Foundation Cardiovascular Epidemiology Unit, Department of Public Health and Primary Care, Strangeways Research Laboratory, University of Cambridge, Wort’s Causeway, Cambridge, CB1 8RN UK; 2 Department of Human Genetics, The Wellcome Sanger Institute, Wellcome Genome Campus, Hinxton, Cambridge CB10 1HH UK; 3 Medical Research Council Biostatistics Unit, University of Cambridge, East Forvie Building, Cambridge Biomedical Campus, Forvie Site, Robinson Way, Cambridge, CB2 0SR UK; 4 The National Institute for Health and Care Research Blood and Transplant Unit in Donor Health and Genomics, Strangeways Research Laboratory, Strangeways Research Laboratory, University of Cambridge, Wort’s Causeway, Cambridge, CB1 8RN UK; 5 Department of Epidemiology and Biostatistics, School of Public Health, Imperial College London, London, UK; 6 Department of Haematology, University of Cambridge, Cambridge Biomedical Campus, Long Road, Cambridge, CB2 0PT UK; 7 National Health Service Blood and Transplant, Cambridge Centre, Cambridge Biomedical Campus, Long Road, Cambridge, CB2 0PT UK; 8 British Heart Foundation Centre of Research Excellence, University of Cambridge, Addenbrooke’s Hospital, Cambridge Biomedical Campus, Cambridge, CB2 0QQ UK; 9 Victor Phillip Dahdaleh Heart and Lung Research Institute, University of Cambridge, Cambridge, CB2 0BB UK; 10 Division of Hematology/Oncology, Boston Children’s Hospital, Harvard Medical School, 1 Blackfan Circle, Boston, MA 02115 USA; 11 Department of Pediatric Oncology, Dana-Farber Cancer Institute, Harvard Medical School, 450 Brookline Ave, Boston, MA 02115 USA; 12 Broad Institute of MIT and Harvard, 415 Main St, Cambridge, MA 02142 USA; 13 Harvard-MIT Health Sciences and Technology, Harvard Medical School, 77 Massachusetts Ave, Cambridge, MA 02139 USA; 14 Department of Haematology, Barts Health National Health Service Trust, London, E1 1BB UK; 15 National Institute for Health and Care Research Cambridge BioResource, Box 229, Addenbrooke’s Hospital, Cambridge Biomedical Campus, Cambridge, CB2 0QQ UK; 16 Wellcome Centre for Human Genetics, University of Oxford, Roosevelt Drive, Oxford, OX3 7BN UK; 17 European Molecular Biology Laboratory, European Bioinformatics Institute, Wellcome Trust Genome Campus, Hinxton, Cambridge CB10 1SD UK; 18 European Molecular Biology Laboratory, Genome Biology Unit, 69117 Heidelberg, Germany; 19 Division of Computational Genomics and Systems Genetics, German Cancer Research Center (DKFZ), 69120 Heidelberg, Germany; 20 Health Data Research UK Cambridge, Wellcome Genome Campus and University of Cambridge, Cambridge, UK; 21 Nuffield Division of Clinical Laboratory Sciences, Radcliffe Department of Medicine, University of Oxford, Headley Way, Headington, Oxford OX3 9DU UK; 22 National Institute for Health Research Oxford Biomedical Research Centre—Haematology Theme, John Radcliffe Hospital, Headley Way, Headington, Oxford OX3 9DU UK; 23 National Health Service Blood and Transplant, Oxford Centre, John Radcliffe Hospital, Headley Way, Headington, Oxford OX3 9DU UK; 24 Health Data Science Research Centre, Fondazione Human Technopole, Viale Rita Levi Montalcini 1, Milan, 20157 Italy; 25 Department of Clinical and Biomedical Sciences, University of Exeter Medical School, Faculty of Health and Life Sciences, RILD Building, Barrack Road, Exeter, EX2 5DW UK; 26 Department of Pediatric Immunology, Rheumatology and Infectious Disease, Emma Children’s Hospital, Amsterdam University Medical Center, Amsterdam, CB2 0PT UK; 27 Department of Blood Cell Research, Sanquin Research and Landsteiner Laboratory, Sanquin, University of Amsterdam, Amsterdam, Netherlands; 28 Department of Immunology and Inflammation, Imperial College London, Commonwealth Building, The Hammersmith Hospital, Du Cane Road, London, W12 0NN UK; 29 Department of Haematology, University College London Hospitals, WC1E 6AS London, UK; 30 Genomics Research Centre, Fondazione Human Technopole, Viale Rita Levi Montalcini 1, Milan, 20157 Italy

**Keywords:** Cell biology, Medical genomics, Haematopoiesis, Drug development

## Abstract

Blood cells contain functionally important intracellular structures, such as granules, critical to immunity and thrombosis. Quantitative variation in these structures has not been subjected previously to large-scale genetic analysis. We perform genome-wide association studies of 63 flow-cytometry derived cellular phenotypes—including cell-type specific measures of granularity, nucleic acid content and reactivity—in 41,515 participants in the INTERVAL study. We identify 2172 distinct variant-trait associations, including associations near genes coding for proteins in organelles implicated in inflammatory and thrombotic diseases. By integrating with epigenetic data we show that many intracellular structures are likely to be determined in immature precursor cells. By integrating with proteomic data we identify the transcription factor FOG2 as an early regulator of platelet formation and α-granularity. Finally, we show that colocalisation of our associations with disease risk signals can suggest aetiological cell-types—variants in *IL2RA* and *ITGA4* respectively mirror the known effects of daclizumab in multiple sclerosis and vedolizumab in inflammatory bowel disease.

## Abstract (web summary)

The authors identify genetic variation associated with properties of the internal biological structures of blood cells in hundreds of genes and show how such discoveries can be used to improve understanding of cellular mechanisms causing disease.

## Introduction

Blood cells play vital roles in human physiology, including in oxygen transport, in haemostasis, and in host defence. Many of the biological functions of blood cells, such as thrombotic aggregation and the killing of pathogens or the killing of virally infected cells, are mediated by proteins stored in cell granules that are released into the extracellular space in response to a stimulus. At present, the functional responses of blood cells to stimuli cannot be measured using high-throughput instruments. Consequently, genetic association studies of cell function traits have been limited to small studies (*n* ≤ 5000) of platelet aggregation phenotypes, which have identified associations in approximately thirty loci^1^. Meanwhile, high-power genome-wide association studies (GWAS) of blood cell traits have concentrated on phenotypes in classical complete blood counts (cCBCs)^2–4^. cCBCs are standard clinical reports, which include measurements of the counts (i.e. concentrations) and average volumes of various types of cell in the peripheral blood. However, cCBCs do not measure the properties of intracellular structures, which play important roles in many functional haematological processes. For example, pathologies of leukopoiesis that result in the absence of specific granules from neutrophils can cause immune dysfunction^5^, but are frequently accompanied by a normal neutrophil count and so cannot be detected by cCBCs.

cCBCs are produced by automated haematology analysers, which usually contain a built in flow-cytometer, which can measure the fluorescence and diffraction of laser light incident on individual blood cells. Variation in structural properties of blood cells—many of functional or clinical relevance—can be detected by flow-cytometry. For example, the intensity of light side-scattered (SSC) by a neutrophil is a measure of the cell’s intracellular organelle complexity, including its granule content. Neutrophil granularity is an important immune phenotype. Activated neutrophils are known to exhibit greater SSC than resting neutrophils^6,7^. Hypo- and hyper-granulated neutrophils are observed in inflammation and also in myelodysplasia^8–11^. Historically, neutrophil granularity was assessed manually by microscopy of blood smears. Such estimates of granularity correlate well with optical measurements made by haematology analyser flow-cytometers^9^. Indeed, flow-cytometry measured neutrophil SSC is a predictor of neutrophil toxic granulation^9^. More generally, a range of flow-cytometry measured properties of platelets, neutrophils, lymphocytes, and monocytes have been implicated as statistical predictors of clinical outcomes, including thrombocytopenic purpura^12^, sepsis^13–15^, myelodysplastic syndromes^16,17^, Sezary disease^18^ and the need for mechanical ventilation in COVID-19 patients^10,19^.

**Fig. 1:**
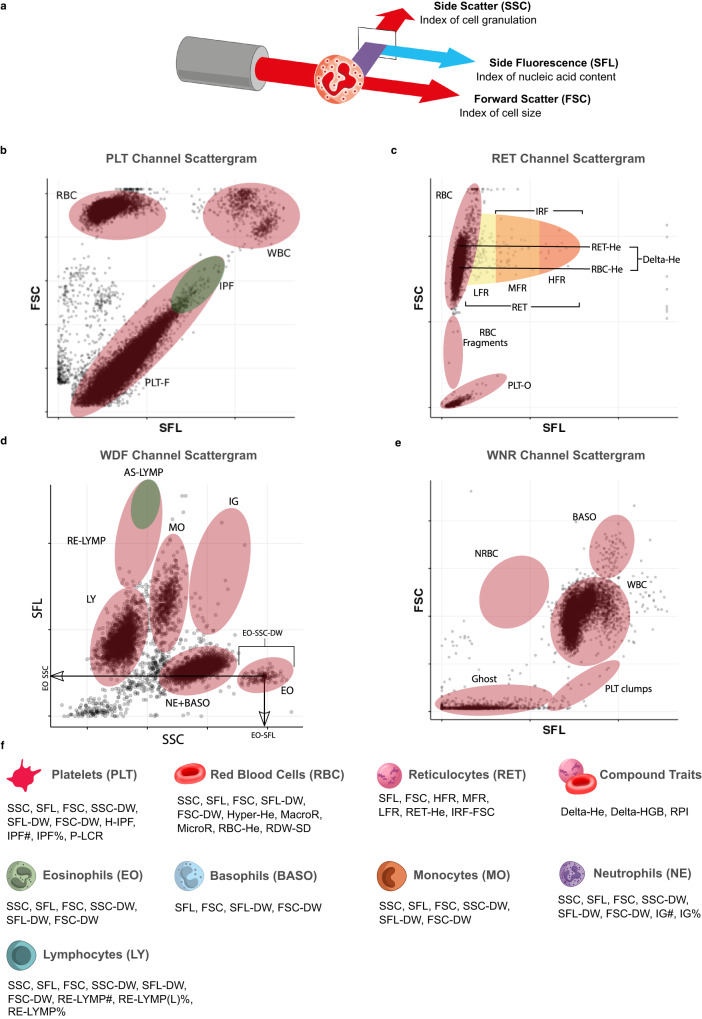
Flow-cytometry traits measured by the Sysmex XN-1000 haematology analyser (adapted from Sysmex XN-1000 Manual^104^). **a** Schematic of a granulocyte cell passing through the laser of the internal flow-cytometer of the analyser. The instrument measures the intensities of incident light scattered sidewise (SSC, cell complexity/granularity) by the cell and forward (FSC, cell volume) by the cell and the intensity of the light which is absorbed by the cell and fluoresced at a new wavelength (SFL, cell nucleic acid content). **b**–**e** Cytometry scattergrams from an arbitrary participant in the INTERVAL study: 2-dimensional projections of the cell level intensity data (SSC, SFL, FSC) measured in each of the four XN-1000 flow-cytometry channels active for the INTERVAL study: PLT-F (platelet flow) channel (**b**), RET (reticulocyte) channel (**c**), WDF (white cell differential) channel (**d**), WNR (white cell and nucleated red cell) channel (**e**). Many of the traits correspond to averages or distribution widths (DWs) of cell level measurements in scattergram regions (indicated approximately by ellipses) occupied by cells of particular types. This is illustrated for three eosinophil traits (in panel **d**). Supplementary Data 2 contains a full description of the measurement procedure for each trait. **f** The 63 cytometry traits classified by the type of cells which they measure: platelets (PLT), mature red blood cells (RBC), reticulocytes (RET), neutrophils (NE), eosinophils (EO), basophils (BASO), monocytes (MO) and lymphocytes (LY). The three compound traits (Delta-HE, Delta-HGB, and RPI) depend on measurements of both mature red cells and reticulocytes. We thank Joanna Westmoreland for the artwork in (**a**) and (**f**).

Here, we report the first large-scale GWAS of flow-cytometry measured non-classical CBC (ncCBC) traits (Fig. 1a–e). We call these phenotypes ncCBC traits because they were acquired using a haematology analyser, the Sysmex XN-1000, but are not included in standard cCBC reports (Fig. 1f). The XN-1000 analyser contains a flow-cytometer, which can detect variation in the intracellular organelle complexity of cells from the intensity of SSC diffracted light. It can also detect variation in the nucleic acid content and membrane permeability of cells from the fluorescence intensity (SFL) of light emitted by a cell staining dye and variation in the size of cells from the intensity of forward scattered (FSC) diffracted light (Fig. 1a–e). The analyser reports individual level summaries of the cell level distributions of SSC, SFL and FSC intensity measurements (as averages or distribution widths over a set of cells of a given type). These phenotypes can capture variation in biological processes with functional or clinical relevance^7,9,20^. By integrating the results of GWAS of ncCBC traits with the results of multi-omic and disease GWAS, we show how flow-cytometry phenotypes can be used to identify the secretory origins of proteins in the blood plasma and used to study the role of blood cells in mechanisms mediating disease risk variation.

## Results

### Hundreds of new genetic determinants of blood cell flow-cytometry traits

**Fig. 2:**
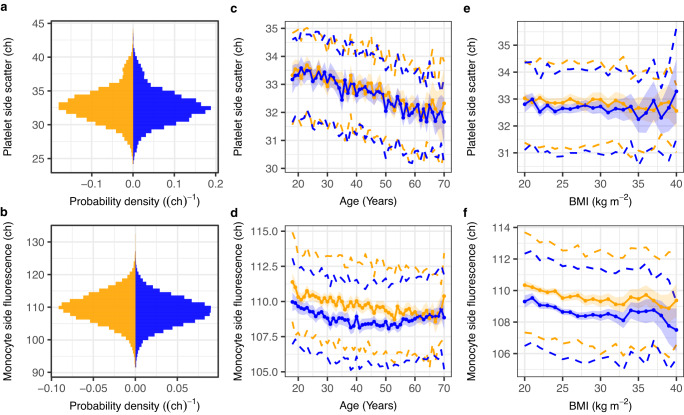
The distributions of selected ncCBC traits and their covariation with age, sex and BMI. Summary plots for two exemplar technically adjusted traits (Methods) using data from participants who contribute to the GWAS of the respective trait. The upper row and lower row panels correspond respectively to the platelet side scatter (PLT-SSC, *n* = 29,675) and monocyte side fluorescence (MO-SFL, *n* = 39,586) phenotypes. **a**, **b** Probability density histograms stratified by sex: female (orange) and male (blue). **c**, **d** Covariation between the phenotype and participant age stratified by sex. Parameters of the stratified trait distributions were estimated in bins corresponding to years of age. The linearly interpolated coloured points show estimates of the within strata-means and the underlying coloured ribbons show the corresponding 95% confidence intervals. The dashed lines show estimates of the upper and lower quartiles. **e**, **f** Covariation between each trait and body mass index (BMI). Estimates of sex stratified summaries were made in bins of 1 kg m^−2^. The components of the plots are as for (**c**, **d**). Analogous plots for all 63 traits are presented in Supplementary Figs. 1–3.

We studied 63 ncCBC phenotypes in INTERVAL (Supplementary Data 1 and 2), a cohort of blood donors, which has been described previously by Moore et al.^21^. Eleven of the traits explicitly summarise cell-level measurements of intracellular complexity/granularity (SSC), sixteen of the traits explicitly summarise cell level measurements of cell nucleic acid content/membrane lipid content (SFL) and fifteen of the traits explicitly summarise cell-level measurements of cell morphology/volume (FSC) (Supplementary Data 2). Ten of the traits are platelet phenotypes, twenty are red cell phenotypes and 33 are white cell phenotypes. After performing exploratory analysis—investigating the distributions of the traits and their co-distributions with various covariates including, age, sex, menopause and BMI (Fig. 2, Supplementary Figs. 1–3, Supplementary Data 3)—we developed models to regress out extraneous variation (Methods). We also investigated covariation between the traits, observing greater correlation on average between traits specific to red cells or platelets than traits specific to white cells, principally because the white cell traits measure properties of biologically heterogeneous subtypes of cells (neutrophils, basophils, eosinophils, lymphocytes and monocytes) (Supplementary Figs. 4–7).

**Fig. 3:**
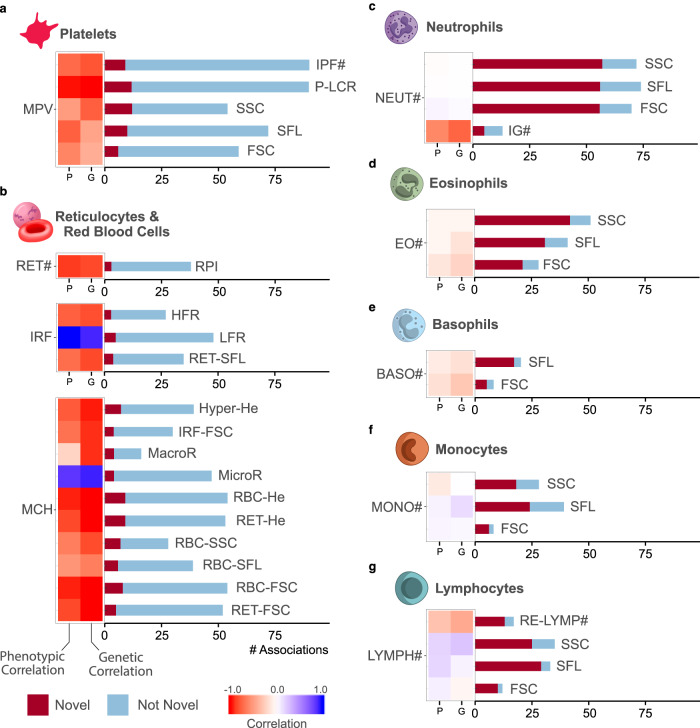
The distribution and novelty of association signals by cell-type. **a**–**g** Each panel presents statistics for selected ncCBC traits of the given cell-type. The heat map on the left of each subplot shows the estimated phenotypic (left) and genetic (right) correlation between the cCBC trait indicated to its left and the ncCBC trait corresponding to each horizontal bar. (Each ncCBC trait has been grouped with the cCBC trait studied in Vuckovic et al.^3,4^ with which it has maximal absolute phenotypic correlation in the study sample.) The bar plot on the right of each subplot indicates the number of distinct (conditionally significant) associations identified for each ncCBC trait and the number of distinct associations with variants that do not fall into a LD clump with a variant reported to be associated with a blood trait of the same cell-type by Vuckovic et al. or Chen et al. (‘Novel’)^3,4^. The absolute genetic correlations between the ncCBC and cCBC traits of white cells are lower than those of red cells and platelets. This is reflected in the variation between cell-types of the proportion of identified associations that are novel. We thank Joanna Westmoreland for the artwork in (**a**–**g**).

We performed univariable genetic association analyses (Supplementary Data 4) in subsets of a complete genetic dataset comprising imputed genotypes for 26.8 million variants in 43,059 European-ancestry participants. Stepwise regression analysis applied to each trait in turn identified 2172 distinct (unadjusted conditional *P*-value < 8.31 × 10^−9^) variant-trait associations (Supplementary Data 4). To identify distinct association signals we pooled the conditionally significant variant-trait associations across the traits and applied a standard linkage disequilibrium (LD, *r*^2^ > 0.8) based greedy clumping algorithm (Methods). This clustered the 2172 variant-trait associations into 849 clumps, 231 of which represented platelet signals (i.e. contained at least one platelet cell-trait associated variant), 211 of which represented red cell signals and 432 of which represented white cell signals (Fig. 3, Supplementary Fig. 8). We compared these associations to those identified by a European-ancestry GWAS of cCBC phenotypes with a 14-fold larger sample size^3^ and a transethnic GWAS of cCBC phenotypes with an 18-fold larger sample size^4^. More than half—242 (56%)—of the 432 white cell association signals were novel—i.e. the corresponding clump did not contain a variant in LD (*r*^2^ > 0.8) with a variant reported to be associated with a white cell phenotype by Vuckovic et al. or Chen et al.^3,4^ (Supplementary Fig. 9). Whereas fewer than a quarter—56 (24%) and 45 (21%) respectively—of the 231 platelet and 211 red cell signals were novel, according to the same criterion. The enrichment of novel associations in white cell traits may be explained by the fact that white cells exhibit greater biological complexity than platelets or red cells, complexity which is captured by ncCBC flow cytometry traits, but not standard cCBC phenotypes. White cells are nucleated and contain complicated intracellular organelles such as granules and vacuoles which differ according to white cell subtype. Both red cells and platelets are anuclear, but only platelets contain granules, which are generally smaller than those of white cells.

### Identification of granule proteins undiscovered by GWAS of classical blood phenotypes

**Fig. 4:**
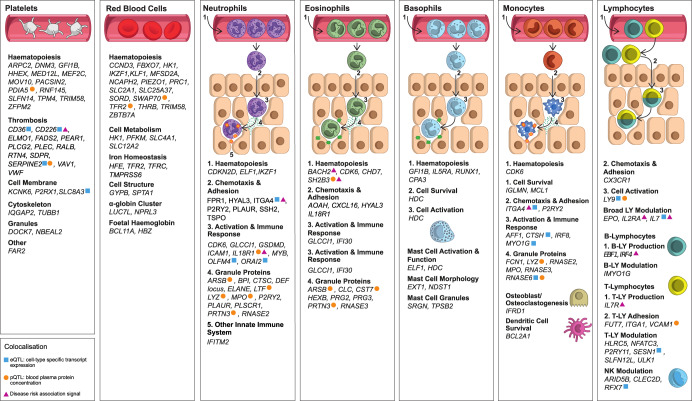
Summary of the biological functions of the genes assigned to associated variants identified from a survey of the literature. Each panel contains a list of genes assigned by VEP or by eQTL/pQTL colocalisation to genetic associations with traits corresponding to the given cell-type, for which a literature search identified evidence of known function. Each list is stratified into functional categories relevant to the cell-type. Supplementary Data 4 contains a complete list of the associated variants, their VEP annotated genes, and relevant references to literature. The coloured symbolic annotations indicate genes assigned to variants which colocalise with eQTL (blue square), pQTL (orange circle), or disease GWAS associations (purple triangle). The gene(s) assigned by eQTL or pQTL colocalisation occasionally differ from the gene(s) assigned by VEP. We thank Joanna Westmoreland for the artwork.

To identify possible molecular mediators of the cell-trait association signals, we annotated each conditionally significant variant with the genes for which VEP predicted the most severe transcriptional consequence (Methods, Supplementary Data 4). We also performed analyses to identify colocalisations of the associations with eQTL for the corresponding cell-types. Of the conditionally significant associations with a VEP gene annotation that exhibited strong evidence (Bayesian posterior probability, PP > 80%) for colocalisation with an eQTL, 67% had a gene annotation consistent with the transcript of the colocalizing eQTL (Supplementary Data 5). An extensive literature search highlighted roles in known fundamental cellular functions including thrombus formation for platelet traits (e.g. *VWF, SERPINE2*), iron homoeostasis for red cell traits (e.g. *HFE, TFRC*) and chemotaxis and adhesion for myeloid white cell traits (e.g. *P2RY2, SSH2*) (Fig. 4; Supplementary Data 4).

We identified genes, not previously found by GWAS of granulocyte traits, that code for various white cell granule proteins including Arylsulfatase B, Lactoperoxidase, RNase 3 and multiple defensins. These proteins have well understood roles in immunity^22^, illustrating the potential for GWAS of ncCBC traits to identify genes critical to blood cell function. Specifically, we identified 35 associations with NE-SSC (neutrophil granularity), 30 associations with EO-SSC (eosinophil granularity), and twelve associations with MO-SSC (monocyte granularity) that did not correspond to any of the genetic associations with cCBC traits reported by Vuckovic et al. or Chen et al. (Supplementary Data 4)^3,4^. These include associations near genes with well understood functions in blood cells, including roles in transcription and translation (*AFF1, RPL3P2, PTBP1*), in exocytosis (which is critical to granule formation and release; *AP1M2, SMAP1*), as granule cargo (*FCN1, HYAL3, PRG2, RNASE3, ARSB, LPO, DEFA*), as lysozyme cargo (*CTNS*, *HEXB*), and in immune response (*FPR1, IFI30, KIAA0922, MYO1G, LGMN*), confirming that our approach has uncovered biologically relevant genes, including genes in molecular pathways modulating cellular complexity and granule formation.

We used data from mass spectrometric profiling of neutrophil granules^23^ to identify the subcellular localisation of proteins expressed by the genes identified by the neutrophil trait GWAS in a wide variety of organelles (Supplementary Data 6). Thirteen clumps contained neutrophil trait associated variants annotated by VEP with genes coding for proteins expressed in azurophilic granules and two clumps contained neutrophil trait associated variants annotated with genes coding for proteins in specific or secondary granules. Cellular granules are also prominent in eosinophils, basophils, and present in monocytes where they play critical roles in innate immune responses by releasing antimicrobial proteins. We identified signals near genes encoding several such proteins, including *HYAL3, PRG2, PRTN3* and *RNASE3* (Supplementary Data 4)^22,24–26^.

### The biogenesis of cellular structures

Many of the intracellular structures generating variation in flow-cytometry phenotypes have developmental origins in the immature precursors of peripheral blood cells. Granule formation, for example, is a cell-type specific process occurring at particular stages of cellular differentiation; the granules of platelets and the granules of granulocytes begin to form in megakaryoblasts and myeloblasts, respectively^27^. Consequently, the absence of a colocalizing mature blood cell eQTL for a cytometry trait association may reflect the fact that the associated genetic variant exerts its variance-generating effect after lineage commitment, but before terminal differentiation. To test this hypothesis, we applied FINEMAP 1.3.1^28^ to identify Bayesian posterior credible sets of variants associated with the ncCBC traits and assessed the enrichment of the variant sets in the nucleosome depleted regions (ATAC-seq) of nine types of progenitor cell (localised in the bone marrow) and nine types of the mature cell (generally localised in the peripheral blood)^29^.

We observed patterns of enrichment in the open chromatin regions of the progenitor cell-types ancestral to neutrophils, eosinophils, monocytes, and lymphocytes (Supplementary Fig. 10a–e) which suggest the possible developmental stages (Supplementary Fig. 10f) at which the cell characteristics corresponding to particular traits (SSC, SFL, FSC) are likely to develop. For instance, neutrophils contain three classes of cytotoxic granules—azurophilic, specific and gelatinase—which are formed sequentially at distinct stages of differentiation^27^. The relative enrichments of genetic variants associated with NE-SSC (neutrophil granularity) in the nucleosome-depleted regions of the hematopoietic stem cell (HSC) and the four types of myeloid progenitor cell (CMP, GMP-A, GMP-B, GMP-C) are consistent with a progressive increase in the accessibility of enhancers regulating granule formation during myeloid differentiation and point to an origin of these granules in lineage-committed myeloid progenitors. In monocytes, we observed an enrichment of MO-SFL (monocyte nucleic acid content) associated variants in the nucleosome-depleted regions of granulocyte-macrophage progenitor cells (GMP)—the differentiation phase of proliferation and cell division— and an enrichment of MO-SSC (granularity) in the nucleosome depleted regions of peripheral blood monocytes, suggesting that granularity may be regulated in the ultimate stages of monocyte differentiation before egress from the bone marrow.

### The cellular origins of plasma proteins

We hypothesised that some genetic variants associated with cytometric traits, in particular genetic variants associated with side scatter traits, which capture the abundance of secretory granules in cells, also influence the concentration of secretory proteins in the blood plasma. To explore this, we used the results of Sun et al., a GWAS of plasma concentrations of 1478 proteins, which identified 1927 associations (protein quantitative trait loci, pQTL)^30^. For 943 of these proteins, there is strong evidence that transcripts are expressed (log_2_FPKM > 1.0, i.e. log_2_ of fragments per kilobase of transcript per million mapped fragments is greater than 1.0) in at least one of the blood cell-types surveyed by the ncCBC phenotypes (megakaryocytes (MKs) and erythroblasts—the respective progenitors of platelets and red cells—and neutrophils, eosinophils, basophils, monocytes, CD4^+^ T cells, CD8^+^ T cells, naive B cells). We performed colocalisation analysis between the pQTL and our cytometry trait-associated conditionally significant variants and identified 61 and 1021 colocalisations (PP > 80%) with cis and trans pQTL, respectively (Fig. 4, Supplementary Data 7). There were 181 proteins with a pQTL that colocalised with ncCBC trait association signal for just one cell-type suggesting that blood cells differentially contribute to the plasma proteome (e.g. *VEGFA*, which encodes vascular endothelial growth factor A, is associated with eight different platelet traits, while *RNASE6*, which encodes RNase K6, is associated solely with monocyte side fluorescence) (Supplementary Data 4). Notably, many associations with variants assigned by VEP (gene of worse consequence, Methods) to genes encoding granule proteins colocalised with pQTL for the corresponding proteins in blood plasma (Fig. 4). Examples include *ARSB* (PP = 99%)*, LY9* (PP = 98%)*, MPO* (PP = 100%)*, PRTN3* (PP = 100%) and *RNASE6* (PP = 99%) (Supplementary Data 7).

### Evidence that FOG2 is a regulator of platelet α-granularity

**Fig. 5:**
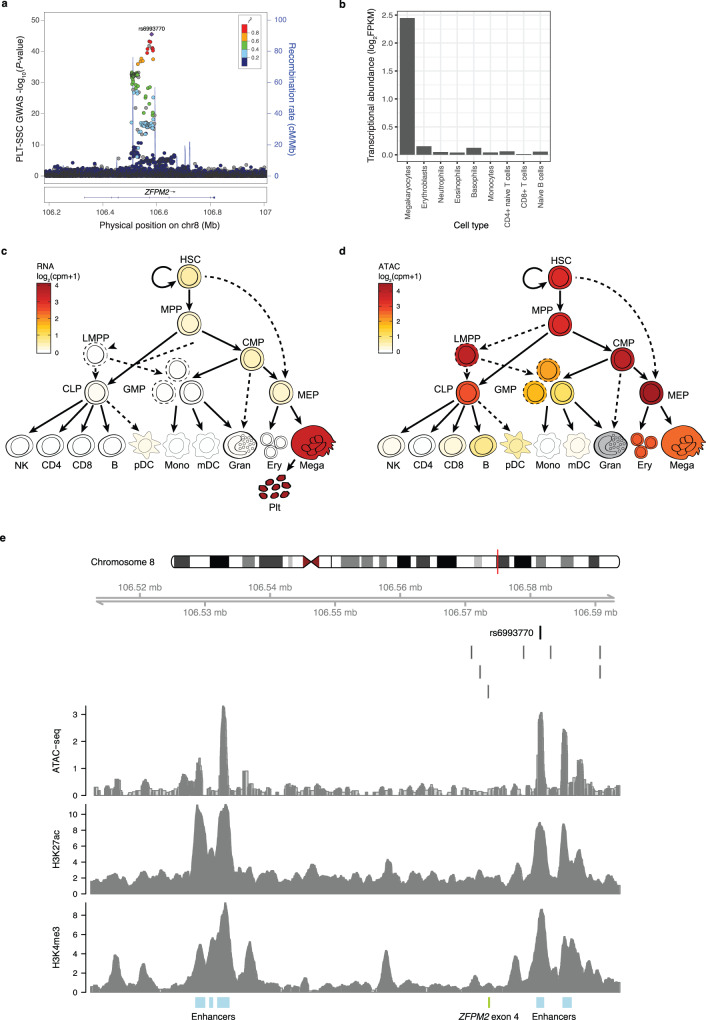
The association of rs6993770 with PLT-SSC is mediated by *ZFPM2* expression. **a** A LocusZoom plot for the *ZFPM2* locus^105^. Each dot corresponds to a variant tested for association. The *x*-axis represents the physical position on chromosome 8 in GRCh37 coordinates. The (left-hand) *y*-axis represents the −log_10_(*P*-value) from a univariable BOLT-LMM test for additive allelic association between the imputed genotypes of the variant and PLT-SSC (*n* = 29,675). The colour of the dot represents the LD (*r*^2^) in the study sample between the corresponding variant and rs6993770. The blue line represents an estimate of the local recombination rate (right-hand *y*-axis). Conditional analysis identified a single association signal in the 82 kb interval of low recombination containing rs6993770. **b** The abundance of *ZFPM2* transcripts (log_2_FPKM) in MKs, erythroblasts, neutrophils, eosinophils, basophils, monocytes, CD4+ naive T cells, CD8+ T cells, and naive B cells, in which cell-types *ZFPM2* transcription is limited to MKs^31^. **c** *ZFPM2* transcript expression is higher in platelets, MKs and their precursor cell-types—MEP (megakaryocyte-erythroid progenitor cells), CMP (common myeloid progenitor), MPP (multipotent progenitor), and HSC (hematopoietic stem cell)—than in other blood cell and blood cell precursor cell-types. **d** ATAC-seq applied to multiple blood cell-types show that rs6993770 lies in an open chromatin region in the platelet precursor cell-types MK, MEP, CMP, MPP and HSC. **e** Measurements of epigenetic activity in MKs across the 82 kb recombination interval containing the association signal. The *x*-axis represents the physical position on chromosome 8. The dark vertical line indicates the position of rs6993770. The nearby light vertical lines indicate the locations of seven variants in high LD (*r*^2^ > 0.9) with rs6993770. The *y*-axis of each panel corresponds to the sequencing read depth of an epigenetic assay. From top to bottom the panels correspond to ATAC-seq (open chromatin), H3K27ac (a mark of active enhancers) and H3K4me3 (a mark of accessibility to transcription factors). The blue rectangles at the bottom of the figure indicate enhancer regions in MKs inferred from a set of six histone modifications (H3K4me1, H3K4me3, H3K9me3, H3K27ac, H3K27me3 and H3K36me3) using the IDEAS chromatin segmentation algorithm^106,107^. The green rectangle indicates the position of exon 4 of *ZFPM2*. Panels c-d are adapted with permission from Ulirsch, J. C. et al. Interrogation of human hematopoiesis at single-cell and single-variant resolution. Nat. Genet. 51, 683–693 (2019), Springer Nature^29^.

Platelet activation is important in thrombus formation, wound healing, inflammation and the chemotaxis and activation of myeloid white cells. Critical to these biological processes are coagulation proteins, growth factors, proteases, chemokines and other signalling peptides that diffuse into the blood plasma when α-granules are released by activated platelets. Our GWAS of PLT-SSC (platelet granularity) identified an association in *ZFPM2* (encoding the transcription factor Friend of GATA-2 or FOG2) (Fig. 5a), colocalizing with trans-pQTL for 24 plasma proteins, of which thirteen are platelet α-granule localised proteins (Supplementary Data 6). RNA-seq data from nine differentiated nucleated blood cell-types^31^ showed that transcripts of *ZFPM2* are substantially expressed solely in MKs (Fig. 5b). Complementary data from a study of the entire hematopoietic system showed that *ZFPM2* is specifically expressed in the MK lineage (Fig. 5c)^29^. A stepwise multiple regression analysis of the platelet granularity phenotype (PLT-SSC) on variants in the *ZFPM2* locus suggested a single conditionally significant association, and fine-mapping of the locus identified a single intronic SNP (rs6993770) as the most probable causal variant (PP = 95%). rs6993770 is located in a region of open chromatin (ATAC-seq) in MKs, which contains histone modifications indicative of a transcriptional enhancer (H3K4 tri-methylation, H3K27 acetylation; Fig. 5d, e). The variant is located 25 b upstream of a GATA motif on the negative strand, and 34 b upstream from the palindromic E-box binding motif CAGCTG. The juxtaposition of these motifs is characteristic of a hematopoietic co-binding site for GATA-1 and TAL1, two of the three key MK lineage determining transcription factors^32–34^. None of the seven variants in high LD (*r*^2^ ≥ 0.9) with rs6993770 were located in regions for which epigenetic data supported causality in MKs (Fig. 5e).

**Fig. 6:**
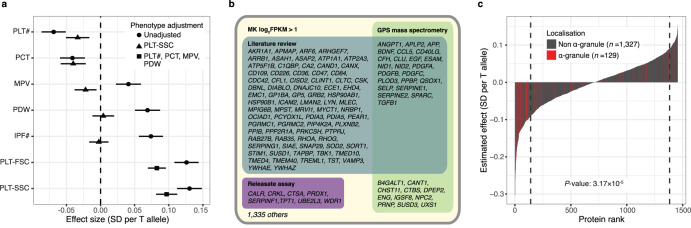
*ZFPM2* is a regulator of platelet α-granularity. **a** Forest plot showing the additive allelic effect of rs6993770-T on the means of the inverse rank normalised distributions of the platelet traits PLT# (platelet count, *n* = 29,657), PCT (plateletcrit, *n* = 28,044), MPV (mean platelet volume, *n* = 28,050), PDW (platelet distribution width, *n* = 28,052), IPF# (immature platelet fraction count, *n* = 30,587), PLT-FSC (platelet volume, *n* = 29,662), and PLT-SSC (platelet granularity, *n* = 29,675) measured in the INTERVAL study. Circles correspond to estimates of direct effects, triangles correspond to estimates of effects adjusted for PLT-SSC and squares correspond to estimates of effects adjusted for PLT#, PCT, MPV, and PDW. The horizontal lines correspond to 95% confidence intervals. The effect of rs6993770-T on PLT-SSC and PLT-FSC does not appear to be mediated substantially through the four cCBC phenotypes. **b** A Venn diagram cross classifying the 1456 genes coding for proteins studied by Sun et al. that are expressed in MKs (mRNA transcript log_2_FPKM > 1)^30^. The classifying categories indicate that the protein was implicated as an α-granule protein coding gene by one of: a literature review (turquoise), detection by mass spectrometry of significant under expression in the platelets of grey platelet syndrome patients (which lack α-granules) compared to those of healthy volunteers (green), identification in the platelet releasate—proteins expelled from activated platelets (purple). **c** The estimated per allele effect of rs6993770-T on the mean concentration of the 1456 plasma proteins. The *y*-axis measures the per allele effect size and the *x*-axis its rank. Bars corresponding to proteins localised to platelet α-granules are coloured red. Proteins with ranks in the tails bounded by the dashed lines exhibit significant evidence for an association with rs6993770-T at a relaxed critical threshold (unadjusted *P-*value < 10^−3^). α-granule proteins are significantly (embedded two-sided Fisher’s exact test unadjusted *P*-value) enriched in the negative compared to the positive tail.

There are well-known associations between rs6993770 and the four platelet cCBC traits: platelet count (PLT), volume (MPV), crit (PCT) and volume distribution width (PDW)^2,35^. We identified new associations with the ncCBC traits IPF#, PLT-SSC, and PLT-FSC (Supplementary Data 4, Fig. 6a). The estimated effect of rs6993770 on mean PLT-SSC was not significantly attenuated by a multivariable linear adjustment for the four cCBC traits, suggesting that the PLT-SSC association is independent of the association with classical platelet phenotypes. Consequently, we hypothesised that *ZFPM2* plays a role in α-granule biology. To investigate this, we considered the effect of rs6993770 on the mean plasma concentrations of 1456 of the proteins studied by Sun et al., for which there was evidence of expression in MKs (RNA-seq log_2_FPKM > 1, Fig. 6b)^30^. rs6993770-T had a significant effect on the mean plasma concentration of 215 of these proteins at a relaxed threshold (unadjusted *P*-value < 10^−3^)^30^. Of the 215 associated proteins, 44 were localised to α-granules and 171 were not. The direction of the effect size estimates of Sun et al. imply that rs6993770-T reduces the mean plasma concentration of 40 (91%) of the 44 α-granule localised proteins but only 101 (59%) of the 171 proteins not localised to α-granules (Fig. 6c). This indicates that rs6993770-T significantly differentially reduces the plasma concentration of proteins expressed in MKs according to their platelet α-granule localisation (Fisher’s exact test unadjusted *P*-value = 3.17 × 10^−5^).

To control for the possibility that the differential effect of rs6993770-T might be explained by differences in expression levels correlated with protein α-granule localisation, we regressed the plasma protein concentration estimated effect sizes of rs6993770-T on a dummy variable indicating α-granule localisation, adjusting for the abundance of the mRNA transcripts in MKs, using data corresponding to the 1456 proteins expressed in MKs (log_2_FPKM > 1). We estimated the additive allelic effect of rs6993770-T on plasma proteins to be 0.038 phenotypic standard deviations lower on average (two sided *t-*test unadjusted *P*-value = 5.6 × 10^−10^) in proteins localised to α-granules compared to other proteins, adjusting for gene expression in MKs (Supplementary Fig. 11). This analysis suggests that in addition to reducing PLT-SSC, rs6993770-T preferentially reduces the concentration of platelet α-granule proteins in plasma over other platelet expressed proteins, thus implicating the *ZFPM2* in platelet granule regulation.

We studied the role of *ZFPM2* in platelet production using an in vitro model of megakaryopoiesis^32^ applied to two independent *ZFPM2* knockouts (KO) (Methods, Supplementary Fig. 12a). In this model, the ablation of *ZFPM2* resulted in a drastic reduction in megakaryopoiesis (Supplementary Fig. 12b, c). Because of the dramatically limited production of MKs by the KO lines, it was not possible to test the effect of *ZFPM2* KO on Sysmex platelet traits or perform further biomolecular analysis. However, our results suggest that expression of *ZFPM2* plays a critical role in platelet biology, with important consequences for platelet biogenesis and α-granule regulation.

Interestingly Klarin et al., recently reported an association between the T allele of rs6993770 and decreased risk of venous thromboembolism (VTE)^36^. They postulated that FOG2 mediates a decrease in the plasma concentration of the principal inhibitor of plasminogen activator PAI-1, which is encoded by *SERPINE1*^37^. The notion that lower levels of plasma PAI-1 cause a reduced risk of VTE is biologically plausible. However, we have shown that rs6993770 is pleiotropic, modifying the process of platelet formation and platelet granule content and the concentrations of many platelet α-granule derived proteins in the blood plasma (Fig. 6), highlighting the importance of broader multi-trait and multi-omic integrative analysis, for aetiological inference and the characterisation of disease risk association signals.

### Flow-cytometry phenotypes, disease aetiology and drug targets

We sought to explore more generally how genetic associations with ncCBC traits can improve or confirm our understanding of the role of blood cells in disease aetiology. We chose to focus on immune, inflammatory and cardiovascular diseases, in which blood cells are known to play a critical role. Firstly, we retrieved publicly available GWAS summary statistics for 15 diseases^38^ and assessed the evidence for colocalisation between genetic associations with ncCBC traits and disease risks (Supplementary Data 8)^39^. We found strong evidence (PP > 80%) for colocalisation between 73 of the variant-ncCBC trait associations—corresponding to 29 variant clumps—and at least one disease association (Supplementary Data 8).

Eight variant clumps contained variants associated with lymphocyte traits that colocalised with genetic associations for the risk of multiple sclerosis (MS), coeliac disease, primary biliary cirrhosis, hay fever/rhinitis or coronary artery disease (Supplementary Data 8). Five of these eight clumps contained variants with lymphocyte trait associations that colocalised with associations for risk of MS, recapitulating the known importance of lymphocytes in the aetiology of MS^40^. The colocalizing associations were located in the gene encoding the transcription factor *BACH2*, in the genes encoding receptors for Interleukin(IL)-2 (*IL2RA*) and IL-7 (*IL7R*) and in the gene encoding IL-7 itself (*IL7*). IL-2 receptor α-chain (the product of *IL2RA*) is the target of the therapeutic antibody Daclizumab, which is known to be clinically effective in the treatment of MS^41,42^, but has been withdrawn due to severe side effects including encephalitis^43–46^.

Alternative therapeutic approaches to MS are required to ameliorate the risk that subsets of patients develop severe side effects on existing drugs. The IL-7 receptor (the product of *IL7R*) is one possible target^47,48^, and genetic evidence suggests that a higher serum concentration of soluble IL-7R increases the risk of multiple sclerosis^49^. A non-synonymous coding variant in *IL7RA* (rs6897932-C) effects alternative splicing causing a two-fold increase in the skipping of exon 6 resulting in production of soluble IL-7R^50,51^. rs6897932-C is in high LD (*r*^2^ = 1) with rs11567705-C (AF = 72.5%) which is associated with increased reactive lymphocyte count (RE-LYMP#, additive allelic effect size=0.09 SD, unadjusted *P*-value = 4.4 × 10^−21^). This association with RE-LYMPH# colocalised with increased risk of MS (PP = 95.7%). Thus our analysis suggests that the effect of soluble IL-7R on MS aetiology may be mediated by modulation of lymphocyte activation as measured by RE-LYMPH#, consistent with the role of IL-7R/IL-7 as an efficacious target for the treatment of MS^50^.

Further examples demonstrate the ability of ncCBC GWAS associations to indicate which blood cell-types may mediate disease aetiology. For instance, rs2124440-A is associated with properties of monocytes (MO-SFL-DW, additive allelic effect size = 0.048 SD, unadjusted *P*-value = 3.4 × 10^−11^; MO-SFL additive allelic effect size = −0.034 SD, unadjusted *P*-value = 2.0 × 10^−6^) and these associations colocalised with lower expression of *ITGA4* in monocytes (CD14, PP = 99.7%) and higher inflammatory bowel disease (IBD) risk (PP = 98.2%). This finding is interesting because Vedolizumab was originally used to treat IBD^52,53^ and was assumed to reduce trafficking of α4β7-positive gut-specific T-helper lymphocyte by diminishing integrin interaction with the mucosal addressin cell adhesion molecule 1 (MadCAM-1)^54,55^. However, a recent study has suggested that the antibody also reduces the ability of monocytes to egress into the colonic mucosa as an alternative mechanism^55–57^ consistent with the results of our genetic analyses of monocyte flow-cytometry traits.

## Discussion

Over the last decade, GWAS have identified thousands of genetic associations with risks of common complex diseases. A major motivation for these studies has been to improve our understanding of the molecular and cellular mechanisms underlying disease aetiology, in order to develop safe and effective pharmacological treatments. Unfortunately, only a fraction of the biological mechanisms underlying the genetic associations with disease risk that have been identified are presently understood. Sometimes even the mediating cell-types and tissues are unknown. One approach to understanding a genetic association with disease risk, is to consider the context of colocalizing genetic associations with other phenotypes that have been published in the literature or online catalogues, a so-called ‘phenoscan’^58,59^. Genetic associations with quantitative traits measuring cell-type specific biological variation (for example, eQTL identified from studies of gene expression variation) can suggest mediating tissue types. Consequently, there is a need to catalogue genetic associations with phenotypes measuring cell-type specific biological variation at different levels of complexity, from variation in molecular abundances to variation in the properties of organelles.

We have studied a new class of flow-cytometry phenotypes, which can capture cell-type specific quantitative variation in the functional structures of blood cells, such as secretory granules. These phenotypes can be measured affordably in tens of thousands of study participants using clinical haematology analysers. GWAS analyses of these traits showed that they are heritable, have complex genetic architectures, and are affected by variation in genes implicated in a variety of molecular and cellular pathways. The broad biological sensitivity of flow-cytometry phenotypes means that their genetic associations have a coarser interpretation than those of many other intermediate quantitative phenotypes, such as molecular abundances (e.g transcriptomics, proteomics or lipidomics). For example, cytometry measured ‘neutrophil granularity’ depends on the average number of granules in peripheral blood neutrophils, and the level of expression of genes coding for proteins in neutrophil granules (e.g. *ELANE, MPO, PRTN3*). rs138303849-C, a variant upstream of *PRTN3* (encoding proteinase-3, PR3) is associated with increased NE-SSC (granularity), NE-SFL (nucleic acid content), NE-FSC (volume), and decreased NE-SSC-DW (SSC distribution width). The allele, which is known to be associated with an increased risk of a form of vasculitis characterised by autoantibodies against PR3^60^, is an eQTL for *PTRN3* in whole blood^61^. We recently showed that the risk allele is associated with increased PR3 concentration in plasma^30^. In the present analysis, we observed that the risk allele and the cis-pQTL for higher plasma PR3 levels all colocalise (PP = 99.9%) with the neutrophil ncCBC associations. This suggests that the vasculitis risk allele not only increases *PRTN3* transcription and PR3 plasma protein levels, but also changes the structural and granular properties of neutrophils.

We have shown more generally how genetic associations with the new traits can be used to interpret colocalizing associations with disease risks and provide evidence to support drug targets, through examples which are corroborated by existing evidence in the literature, including the role of lymphocyte activation in IL-7R/IL-7 mediated risk of MS and the role monocytes *ITGA4* mediated risk of inflammatory bowel disease. We have also shown that genetic associations with flow-cytometry traits can identify key genes regulating the formation and retention of intracellular structures. In particular, our multi-omic analysis of the association in *ZFPM2* with PLT-SSC (platelet granularity), has shown that the transcription factor FOG2 is a probable regulator of platelet α-granularity, which influences the concentrations of a multitude of α-granule proteins in the blood plasma.

Our study has some limitations. Firstly, because participants in the INTERVAL cohort are overwhelmingly of European ancestry, we have not been able to study genetic variation extraneous to the European ancestry population. Secondly, because most of the ncCBC traits are not intended for clinical use, they are subject to greater technical variability than cCBC traits. Nevertheless, we are confident that we have been able to remove much of this variation statistically. Genetic studies, which seek to estimate differences in phenotypic averages between genotype groups, do not rely on the calibration of phenotypes against an absolute standard. However, investigators who wish to perform non-genetic studies of ncCBC traits should be aware of the potential for between instrument variation. Thirdly, because we have no information about the medications taken by the participants, we were unable to exclude the possibility of confounding due to differential prescribing by genotype. However, we expect that such confounding is unlikely, given the general good health of blood donors and the fact that most common genetic variants have a modest effect on disease risk and hence prescribing risk. Fourthly, because the ncCBC phenotypes are novel to genetics, we do not have access to a replication dataset. However, CBC trait associations identified in INTERVAL using the same analysis protocol have a high rate of replicability in other datasets (e.g. UK Biobank). This gives us confidence in our results, although the ultimate validation of any genetic association must rely on cellular and functional laboratory follow up. Finally, although flow-cytometry traits are able to capture aspects of biological variation related to cell function, they lack the clean biological interpretation of phenotypes designed to measure particular cellular mechanisms, such as the response of cells to perturbation by an agonist. In future, the development of efficient cell-type specific assays may enable large-scale population studies of isolated functional processes such as activation, degranulation and cell motility likely to play a role in the aetiologies of cardiovascular and immune disorders.

## Methods

### INTERVAL study

The INTERVAL study was a randomised trial of approximately 45,000 blood donors aged eighteen years or older who were recruited at 25 NHSBT (National Health Service Blood and Transplant) static donor centres across England^21^. The study was approved by the Cambridge East Research Ethics Committee and we complied with the relevant ethical regulations. Informed consent was obtained from all participants during recruitment. Individuals who have suffered recent illness or infection are ineligible to donate blood. Consequently, the participants were predominantly healthy at the time of recruitment. The participants completed a baseline survey which included questions about their lifestyle and state of health, including their smoking habits, their alcohol consumption habits, whether they suffered from doctor-diagnosed anaemia, their use of medication (hormone replacement therapy, iron supplements) and their menopausal status. At baseline, participants were randomly allocated to a sex specific trial arm, according to which men were asked to donate blood every twelve, ten or eight weeks and women were asked to donate blood every sixteen, fourteen or twelve weeks. Participants gave blood samples for research purposes at baseline and at a follow up visit approximately two years later. In the intervening period they were asked to donate blood according to the schedule of their allocated trial arm. The blood samples given for research were collected from a pouch attached to a standard blood collection unit. The samples for CBC analysis were stored in 3 ml EDTA tubes which were inverted three times before being transported in rigid boxes at ambient temperature, via three NHSBT holding sites (Manchester, Colindale, and Bristol), to UK Biocentre, Stockport, Greater Manchester. Here, extended clinical blood count reports, which include measurements of cCBC and ncCBC traits, were generated using a pair of Sysmex XN-1000 analysers. Respectively, 72% and 98% of samples were processed within 24 h and 48 h of venipuncture.

### Sysmex XN-1000 automated haematology analysis

The Sysmex XN series of haematology analysers can be configured to use various combinations of seven measurement channels: an impedance channel, a photometric channel and five flow-cytometry channels. The analysers used for the INTERVAL study were configured to use the following six channels:

- RBC/PLT (red cell and platelet counts/volumes using Coulter’s impedance principle)
- HGB (photometric measurement of total blood HGB)
- WNR (white cell and nucleated red cell flow-cytometry)
- WDF (white cell differential flow-cytometry)
- RET (reticulocyte and red cell flow-cytometry)
- PLT-F (platelet flow-cytometry).

The channels used to measure each of the 63 traits we studied are indicated in Supplementary Data 2. These traits include all the continuously distributed (i.e. without a discrete component) variables reported by the analysers (when configured as described) that are not cCBC traits.

The analyser generates an aliquot of blood for each measurement channel. The flow-cytometry channel aliquots are diluted (1:200 for RET/PLT-F channels, 1:60 for other channels) and pre-treated with channel-specific reagents which differentially lyse and perforate cells by type. The aliquots are then treated with channel specific reagents containing dyes that bind to nucleic acids in organelles and the nucleus. The PLT-F flow-cytometry channel uses a reagent containing an oxazine based dye (Fluorocell PLT), while the other flow-cytometry channels use reagents containing a polymethine dye (Fluorocell WNR, Fluorocell WDF and Fluorocell RET). Each flow-cytometry channel measures three cell-level properties of each particle in the blood aliquot: side fluorescence, forward scatter and side scatter. Side fluorescence is a measure of cell nucleic acid content, but also depends on cell membrane lipid content; forward scatter is a measure of cell size; side scatter is a measure of cell granularity. The cell-level measurements are used by the analyser to classify cells by type (Fig. 1). The classification algorithms are commercially confidential. Individual-level traits are calculated by the analyser from summaries of the cell-type specific cell-level measurements.

The analysers were calibrated using an artificial blood containing stabilised human and animal cells by a Sysmex certified engineer, upon installation and subsequently every few months. The same material, prepared in three concentrations, was used by UK Biocentre staff to make daily quality control measurements. Sysmex reference analysers in Kobe, Hamburg and Chicago make routine measurements of the artificial blood to provide a reference for engineer calibration and for daily comparison with the local quality control measurements, so that any malfunction of an analyser between scheduled engineer visits can be identified.

### Selection of phenotype data

For each participant, where possible we used the ncCBC measurements derived from the blood sample donated at baseline. For a subset of traits, the data from the baseline sample were unavailable for some participants and consequently we substituted the data derived from the analyses of the samples donated at the two year follow up visit. In particular, the PLT-F flow channel of one of the two analysers was incorrectly configured during the first 90 days of the study. Data were therefore missing for the traits measured by this channel during this period for approximately half the blood samples analysed.

We limited our analysis to samples that were analysed within 36 h of venipuncture. We excluded data points for which the analyser ‘flag’ variables indicated a sample or measurement abnormality (e.g. due to platelet clumping). We also excluded measurements if they were made on a day for which fewer than ten measurements of the corresponding trait were available from the corresponding analyser, or if they were made on a day when the median of the measurements of the trait made by the analyser deviated substantially (more than 8 median absolute deviations) from the global median of the measurements made the analyser over the course of the study.

### Removal of extraneous trait variation

Haematology analyser data contain both technically and biologically mediated variation. Where this variation has a non-genetic origin it is a source of noise, which reduces the power of GWAS analysis. We adjusted the data from each trait in two stages using generalised additive models (GAMs) to remove phenotypic variation likely to be extraneous. We identified variables to include as predictors in the GAMs at each stage by consulting haematologists and performing exploratory visual and linear regression analyses. In the first stage, we adjusted each trait to remove technical variation and variation explained by the time at which the blood sample was analysed. The latter variation includes variation due to machine drift, variation caused by machine calibration and variation due to seasonal changes in the physiology of donors. In the second stage, we adjusted each trait for various environmental and physiological factors thought likely to generate variation in blood traits, but unlikely to be colliders of genotype and phenotype. At each stage, we adjusted the traits differently according to whether they were directly measured by the analyser or derived by the analyser from the directly measured traits (Supplementary Data 2). The directly measured traits were adjusted using regression models and the derived traits were re-calculated accordingly from the measured traits. Before each regression adjustment we log-transformed the measured traits that are positively supported (such as cell counts) and logit-transformed the measured traits that are proportions or percentages.

In the first stage, we adjusted each transformed trait for the time duration between the start of the study and the analysis of the blood sample, the time duration between venipuncture and the analysis of the blood sample, the day of the week, the number of days between the start of the year and the day of the measurement, and the instrument used for the analyse. The adjustments were made by fitting a GAM (Generalised Additive Model, R package *mgcv*) to each trait, using splines to model the dependence of the trait on the covariates jointly. The models were based on the regression Eq. (1), in which a regression coefficient corresponding to each term in square brackets has been suppressed for simplicity.

1 $${\mathbb{E}}a({y}_{i})=	 s[t(i)\otimes m(i)]+c[{t}_{{{{{{\rm{year}}}}}}}(i)]+tp[({t}_{{{{{{\rm{day}}}}}}}(i),{t}_{{{{{{\rm{ven}}}}}}}(i))\otimes (m(i),I(i))]\\ 	+\mathop{\sum }\limits_{D\in \{{{{{{\rm{tues}}}}}},{{{{{\rm{wed}}}}}},\ldots {{{{{\rm{sun}}}}}}\}}[{\mathbb{1}}_{D(i)=D}]$$

Here:

- *i* is an index for the observation (the blood donor);
- *y*_*i*_ represents the measured trait value and *a*(*y*_*i*_) the value after its initial transformation;
- *t*(*i*) denotes the number of seconds between the start of the study and the measurement of the trait value;
- *m*(*i*) is a categorical variable with two levels, representing the haematology analyser used to record the measurement;
- *t*_year_(*i*) is the number of seconds between midnight on 1^st^ of January in the year the measurement was made and the time at which the measurement was made;
- *t*_day_(*i*) is the time of day that the measurement was made, in seconds after midnight;
- *t*_ven_(*i*) is the time duration in seconds between venipuncture and the midnight immediately preceding the measurement;
- *I*(*i*) is a binary variable which indicates whether *t*_ven_(*i*) was imputed (rather than observed), imputation was by the median values of the observed *t*_ven_(*i*) variable;
- *D*(*i*) is a categorical variable with seven levels, representing the day of the week on which the measurement was made, the baseline category is monday;
- *s*[] represent a P-spline smoothing spline;
- *c*[] represents a cyclic smoothing spline;
- *tp*[] represents a thin plate spline smoothing spline;
- ⊗ indicates a binary operator, which expands additively to generate main effects and interaction terms.

For each trait, we took the residuals and re-centred them to have mean $\overline{a({y})}$, the mean of the unadjusted *a*-transformed traits. We called the variables obtained after applying the transformation *a*^−1^ to these re-centred residuals, the *technically adjusted traits*.

In the second stage, we further adjusted each trait for covariates measured at baseline and identified by haematologists as likely to generate variation in blood traits, this time using a GAM based on the regression Eq. (2), in which a regression coefficient corresponding to each term in square brackets has been suppressed for simplicity.

2 $${\mathbb{E}}b({y}_{i})=	 s\left[{{{{{\rm{age}}}}}}(i) \otimes {{{{{\rm{meno}}}}}}(i)\right]+tp\left[({{\log }}({{{{{\rm{wgt}}}}}}(i)),{{\log }}({{{{{\rm{hgt}}}}}}(i)))\otimes {{{{{\rm{meno}}}}}}(i)\right] \\ 	+[{\mathbb{1}}_{{{{{{\rm{wgt}}}}}}.{{{{{\rm{na}}}}}}(i)}]+[{\mathbb{1}}_{{{{{{\rm{hgt}}}}}}.{{{{{\rm{na}}}}}}(i)}]+\mathop{\sum}\limits_{d\in K}[{\mathbb{1}}_{{{{{{\rm{drk}}}}}}(i)=d}]+\mathop{\sum}\limits_{a\in A}[{\mathbb{1}}_{{{{{{\rm{alc}}}}}}(i)=a}]+s[{{{{{\rm{pckyrs}}}}}}(i)] \\ 	+\mathop{\sum}\limits_{m\in M}[{\mathbb{1}}_{{{{{{\rm{smk}}}}}}(i)=m}]+\mathop{\sum}\limits_{f\in F}[{\mathbb{1}}_{{{{{{\rm{sfrq}}}}}}(i)=f}]+[{\mathbb{1}}_{{{{{{\rm{pckyrs}}}}}}.{{{{{\rm{na}}}}}}(i)}]+\mathop{\sum}\limits_{r\in R}[{\mathbb{1}}_{{{{{{\rm{arm}}}}}}(i)=r}]$$

Here:

- *b*(*y*_*i*_) is the residual from the first stage regression model corresponding to observation *i*;
- age(*i*) is the age of the participant;
- meno(*i*) is a categorical variable with five levels indicating the menopausal status of the participant, taking values in {post, pre, hysterectomy, male, NA};
- wgt(*i*) is the weight of the participant (missing data were imputed by the population mean);
- hgt(*i*) is the height of the participant (missing data were imputed by the population mean);
- drk(*i*) is a categorical variable with four levels indicating the drinking status of the participant, *K* = {previous, current, NA}, the baseline category is ‘never’;
- alc(*i*) is a categorical variable with six levels indicating the alcohol consumption rate of the participant, *A* = {rarely, 1 to 3 months, 1 to 2 weeks, 3 to 5 weeks, most days}, the baseline category is ‘never’;
- pckyrs(*i*) is the number of pack-years the participant has smoked (missing data were imputed by the population mean);
- smk(*i*) is a categorical variable with four levels indicating the smoking status of the participant, *M* = {previous, current, NA}, the baseline category is ‘never’;
- sfrq(*i*) is a categorical variable with six levels indicating the smoking frequency of the participant, *F* = {special occasions, rarely, occasional, most days, every day}, the baseline category is ‘never’;
- arm(*i*) is a categorical variable with six levels indicating the INTERVAL trial arm to which the participant was assigned (see ‘INTERVAL Study’ above), *R* = {M10, M12, F12, F14, F16}, the baseline category is ‘M8’;
- wgt.na(*i*), hgt.na(*i*), pckyrs.na(*i*), evaluate to true if the participant has missing data respectively for weight, height, or pack years smoked and otherwise evaluate to false.

### Outlier removal and trait transformation

For each trait, we removed data points for which the adjustment was very large and removed data points for which the adjusted values were outliers in the adjusted distributions. Specifically, we took the residuals from the second stage GAM adjustment and removed data points for which the difference between the raw measured value (on the adjustment scale) and the adjusted value was more than 3.5 median absolute deviations from the median of the distribution of such differences. We then removed data points lying more than 4.5 median absolute deviations from the median of the distribution of the residuals. We also performed multivariate outlier removal within groups of phenotypes corresponding to each cell-type, by computing the sum of squares of the leading *d* principal components scores where *d* is the number of independent measurements required to compute the variables in each group and removing any data points for which the sum of squares fell into the upper 10^−7^ tail probability of a χ_*d*_^2^ distribution. Finally, we quantile-inverse-normal transformed the trait data, stratifying by haematology analyser, sex and menopausal status. The final numbers of participants that contributed to the GWAS of each trait are given in Supplementary Data 2.

### Presentation of exploratory visual and linear regression analyses

Although the covariation between the ncCBC traits and biological variables is likely to be of interest to researchers and clinicians, it is difficult to interpret the estimates of coefficients of the GAMs used to adjust the phenotypes. Consequently, we reperformed some of the visual (Fig. 2, Supplementary Figs. 1–3) and linear regression analyses (Supplementary Data 3) used to explore the relationships between ncCBC traits and the variables age, sex, menopausal status and BMI, using the technically adjusted trait data but restricting to the set of participants contributing to the GWAS. We performed a multiple linear regression of each trait on sex, menopausal status and age to estimate:

- the mean of each trait in males, pre-menopausal females and post-menopausal females adjusted for age (estimates correspond to the mean age in the study sample, which is 43.7 years) and
- the effect of age on the mean of the trait while adjusting for sex and menopause.

We also performed a multiple linear regression of each trait on sex, menopause status, age and BMI to estimate the effect of BMI on each trait while adjusting for sex and menopause status.

### Genotyping, quality control of genetic data and imputation of unmeasured genotypes

DNA extraction was performed at LGC Genomics (UK) from buffy coat using a Kleargene method. Samples were packaged and shipped to Affymetrix (now Thermo Fisher Scientific; Mountain View, CA, USA) in 96-well barcoded plates, two wells of which were left empty for standard Affymetrix controls. A sample selection algorithm was used to ensure that the samples on each plate came from participants with a representative distribution of recruitment centre, recruitment date, regional hub and sex. A PicoGreen-based method was used to identify plates with a high proportion of low DNA concentration samples, which were replaced prior to genotyping. Genotyping was performed on the Affymetrix GeneTitan Multi-Channel Instrument according to the Affymetrix Axiom 2.0 Assay Automated Workflow. Genotypes were called in batches of approximately 50 plates (4800 samples) using the Axiom GT1 algorithm implemented in the Affymetrix Power Tools software package.

A full description of the quality control procedures applied to the INTERVAL genotype data can be read in the Methods section of Astle et al.^2^. Data from samples showing evidence of DNA contamination were excluded. Data from samples that were genetic duplicates, from samples corresponding to related groups of participants and from samples from non-European ancestry participants were identified and removed. Data from samples with discordant self-reported and genetically determined sex were removed. Genetic duplicates and relatives were identified using a methods-of-moments estimator for coefficients of pairwise relatedness. Participants were considered to be of European ancestry if their scores on the first two principal components of genetic variation fell below component-specific thresholds in a dataset combining the genotyped INTERVAL participants and genotyped individuals in the major population groups of the 1000 Genomes project. These thresholds were determined by considering the bivariate distribution of principal components scores of individuals self-declaring their ethnicity as ‘White’. Non-autosomal variants, variants with a low call rate, multi-allelic variants and variants showing significant (unadjusted *P*-value < 5 × 10^−6^) deviation from the Hardy-Weinberg equilibrium were removed. The data were phased using SHAPEIT3, following which the genotypes of 87,696,910 variants were imputed from the combined 1000 Genomes Phase 3-UK10K panel using the PBWT imputation algorithm on the Sanger Imputation Server (https://imputation.sanger.ac.uk)^62^.

### Univariable genome-wide association analysis

We used BOLT-LMM version 2.3.1. to perform univariable (i.e. a single genetic variable) linear mixed model regression of each ncCBC phenotype on the imputed alternative allele dose at each of the 29.5 million genetic variants which passed quality control filters (MAF > 0.04%, INFO score > 0.4). We included as covariates, dummy variables indicating the donor clinic at which the blood sample was taken and the score vectors corresponding to the leading ten principal components of genetic variation in the study sample. The Genomic Control inflation factor λ-values ranged between 1.001 and 1.058 suggesting that the mixed model analysis and principal components covariates adequately adjusted for any confounding by relatedness or large scale population stratification^63^.

### Conditional analysis

For each trait, we performed stepwise multiple linear regression analyses to identify a parsimonious subset of genetic variants explaining the univariable genome-wide significant (Wald test unadjusted *P*-value < 8.31 × 10^−9^) associations^64^. We partitioned the set of genome-wide significant variants for each trait into the maximum number of subsets (‘blocks’) such that no pair of variants in distinct subsets were separated by fewer than 10 Mb. We implemented a version of Efroymson’s stepwise regression algorithm using the *fastLM* function in the R package *RcppEigen* and applied it to the variants in each block. We initialised the algorithm by specifying a linear regression model *M*, ‘the current model’ that included the covariates used in the univariable GWAS analysis (PC score vectors and clinic), but no genetic variants. We then performed iterations as follows:

1. For each variant not currently included in *M*,
  augment *M* with the variant to create M* and compare M* to *M* by a likelihood ratio test; compute the corresponding *P*-value.
2. Identify the variant corresponding to the lowest *P-*value in the set of model comparisons performed in step 1. If that *P*-value is lower than 8.31×10^−9^ and does not have a LD *r*^2^ score higher than 0.9 with any variants already in the model then update *M* by adding the variant to the model. If no such variant exists, terminate the algorithm.
3. For each variant currently included in *M*,
  reduce *M* by removing the variant to create M* and compare M to M* by a likelihood ratio test; compute the corresponding *P*-value.
4. Identify the variant corresponding to the greatest *P-*value in the set of model comparisons performed in step 3. If that *P*-value is greater 8.31×10^−9^ then update *M* by removing the variant from the model and return to 3.
5. Return to 1

Finally, for each trait, we ran the algorithm again, but beginning at step 3 and initialising *M* as the union of the sets of variants selected by the block-wise analyses for the trait. The terminating model of this run contains the set of ‘*conditionally significant variants*’ for the trait.

### Fine mapping of association signals

We performed statistical fine-mapping by applying FINEMAP 1.3.1^28^ to each of a set of genomic windows covering the association signals. We generated the windows for each trait by centreing a 500 kb precursor window on each conditionally significant variant and merging those that overlapped. The resulting windows ranged in size from 500,000 b to 599,652 b. For each application of FINEMAP, we specified the maximum number of causal variants (–n-causal-snps option) to be the number of conditionally significant variants in the window. We set the prior standard deviation on the effect size (–prior-std option) to 0.08. We computed the linkage disequilibrium (LD) correlation structure of the variants in each window from the imputed genetic dataset used for the univariable GWAS analysis. We computed a 95% Bayesian credible set of variants for each trait-window as the minimal set of variants jointly covering at least 95% of the posterior probability of association.

### Linkage disequilibrium clumping

The stepwise model selection procedure of the conditional analysis was performed independently for each ncCBC trait. Consequently, a genetic variant causally associated with multiple ncCBC traits may generate association signals that are tagged by different conditionally significant variants for different traits. To identify variants likely to represent common association signals, we sought to partition the full set of conditionally significant variants into sets of variants connected by chains of high LD. We applied the greedy clumping algorithm in PLINK 1.90b3l, which assigns each variant to a distinct clump and then iteratively merges pairs of clumps if there exists a variant in each clump such that the pair of variants is in high LD. We used a threshold of 0.8 on the *r*^2^ measure of LD to merge clumps.

### Identification of association signals not reported by Vuckovic et al. or Chen et al.

In order to identify association signals not previously reported in large cCBC GWAS we declared a clump to represent a ‘novel’ blood cell trait signal if it did not contain a variant in strong LD (*r*^2^ > 0.8) with a conditionally significant variant reported by Vuckovic et al. or Chen et al.^3,4^. Following this, we performed an analysis at the cell-type (platelet, red cell, neutrophil, eosinophil, basophil, monocyte, lymphocyte) level. Each clump containing a variant associated with a trait of a given cell-type was declared to represent a novel association with that cell-type if it did not contain any variants in strong LD (*r*^2^ > 0.8) with a variant identified by the conditional analyses of Vuckovic et al. or Chen et al. to be associated with cCBC traits of the same cell-type (Table 1).

**Table 1:** The cell-type-specific cCBC traits studied by Vuckovic et al. and Chen et al. classified by cell-type

cCBC trait(s)	Cell-type
MPV, PCT, PDW, PLT#	Platelets
HCT, HGB, HLSR#, HLSR%, IRF, MCH, MCHC, MCV, RBC#	Red cells
NEUT#	Neutrophils
EO#	Eosinophils
BASO#	Basophils
MONO#	Monocytes
LYMPH#	Lymphocytes

### Gene assignment

We used Variant Effect Predictor (VEP) version 84 to annotate the conditionally significant variants with the gene symbols of enveloping or proximal (within 5 kb) transcripts (Supplementary Data 4)^65^. All gene transcripts were considered. If a variant was enveloped by or proximal to the transcript of more than one gene, we reported only the genes for which the variant was predicted to have the maximally severe transcriptional consequence according to the Ensembl ranking of transcriptional consequences. We applied the *alias2symbol* function in the R package *‘limma’* to map alias gene names to HGNC gene symbols. We excluded genes without an HGNC symbol.

### Genetic correlation

We estimated the pairwise genetic correlation between each pair of ncCBC traits and between each pair of ncCBC and cCBC traits in the European ancestry population by applying LD Score Regression^66^. We used the LD scores supplied with the LDSC software, which were pre-computed from the 1000 Genomes European data at the 1.2 million common SNPs identified in the CEU population by the HapMap 3 project. We used our own GWAS summary statistics for the ncCBC traits and those of Vuckovic et al. for the cCBC traits^3^.

### Colocalisation with eQTL

We applied *gwas-pw* to identify ncCBC genetic association signals that colocalised with blood cell eQTL^39^. We performed eQTL mapping, in windows extending 1MB from each gene boundary, in platelets (*n* = 424), CD15^+^ neutrophils (*n* = 300) and CD14^+^ monocytes (*n* = 1490), CD4^+^ T-cells, CD8^+^ T-cells and CD19^+^ B-cells using genetic and transcriptomic data obtained from CEDAR, Cardiogenics, the Wellcome Trust Centre for Human Genetics (WTCHG), and BLUEPRINT. Unmeasured genotypes were imputed using the Haplotype Reference Consortium release 1.1 reference panel. These data contributed to an eQTL meta-analysis which has been published in Võsa et al.^67^. We performed colocalisation analysis between genetic associations from 35 cell-type matched ncCBC cytometry traits and the eQTL. Association signals from H-IPF, P-LCR, PLT-SSC, PLT-SFL, PLT-FSC, PLT-SSC-DW, PLT-SFL-DW, and PLT-FSC-DW were colocalised with eQTL of platelets. Association signals from NE-SSC, NE-SFL, NE-FSC, NE-SSC-DW, NE-SFL-DW, NE-FSC-DW, EO-SSC, EO-SFL, EO-FSC, EO-SSC-DW, EO-SFL-DW and EO-FSC-DW were colocalised with eQTL of CD15^+^ cells. Association signals from MO-SSC, MO-SFL, MO-FSC, MO-SSC-DW, MO-SFL-DW, and MO-FSC-DW were colocalised with eQTL from CD14^+^ cells. Finally, association signals from RE-LYMP#, RE-LYMP(L)%, RE-LYMP%, and LY-SSC, LY-SFL, LY-FSC, LY-SSC-DW, LY-SFL-DW, and LY-FSC-DW were colocalised with eQTL of CD4^+^, CD8^+^, and CD19^+^ cells.

A colocalisation analysis was performed between the ncCBC association signal of each conditionally significant variant and each eQTL of the corresponding cell-type, providing that the variants were in strong LD (*r*² > 0.8). Each analysis was performed using data from the recombination region of Berisa et al.^68^ containing the conditionally significant variant. If the region contained multiple conditionally significant variants of the ncCBC trait, we regressed the ncCBC trait on the conditionally significant variants that were not in strong LD (*r*² > 0.8) with the variant of interest and used the resulting residuals as a phenotype for univariable association tests, to obtain ncCBC association statistics for each variant in the region with the effect of secondary signals removed. We filtered the results to report only those which are ‘highly likely’ to represent true colocalisation: those with a posterior probability greater than 80%^30^ (Supplementary Data 5).

### ATAC-Seq enrichment analysis

Following the procedure described by Ulirsch et al., we applied g-chromVAR to compute the enrichment of genetic variants associated with ncCBC traits in the nucleosome depleted regions of the genome of eighteen blood cell populations representing various stages of differentiation^29^. Briefly, regions depleted in at least one cell-type were approximated using 500 b wide windows, each centred at the summit of one of a set of ATAC-seq read peaks selected by applying a consensus finding method to the union of peaks called across the eighteen cell-types. For each cell-type, the ATAC-seq fragment counts in each region were weighted by the sum of the FINEMAP posterior probability of variants in the region, restricted to those variants for which the posterior probability of association was greater than 0.1%. The genome-wide sum of these weighted counts was compared to its expectation under a null model which assumed no enrichment and controlled for GC content and average peak intensity.

### Colocalisation with pQTL

We applied *gwas-pw* to identify ncCBC genetic association signals that colocalised with the genetic associations of 1478 blood plasma proteins identified by Sun et al.^30^. We followed the procedure described for the eQTL colocalisation analysis, except that the regional summary statistics for genetic association with the plasma protein concentrations were adjusted to remove local secondary signals, following the procedure previously described to remove secondary associations from the ncCBC summary statistics (Supplementary Data 7).

### Identification of platelet α-granule proteins

Firstly, we conducted a literature review to identify proteins localised to platelet α-granules by mass spectrometry^69–71^, electron microscopy, immunogold labelling, or platelet sub-fractionation^72–80^; cytoskeletal proteins were excluded. Proteins identified in the platelet releasate were included, absent other evidence that they were α-granule proteins^81–83^. Secondly, we performed a differential abundance analysis comparing the platelets of patients with grey platelet syndrome (GPS; *n* = 5), which lack α-granules, to those of healthy controls (*n* = 5). Platelets were isolated, analysed by mass spectrometry, and normalised abundance values (NAVs) were calculated for each protein detected^84^. We performed two-sample Student’s *t*-tests (without assuming equal variances) to test for a difference in the mean of the log NAVs of each protein between the two conditions. Proteins for which the test was significant (unadjusted *P*-value ≤ 0.05) and for which the log-ratios were greater than 2 standard deviations from the median were considered to be differentially abundant. Differentially abundant proteins that were less abundant in GPS platelets than controls were assumed to be localised to the platelet α-granule.

### The effect of rs6993770 on the average plasma concentrations of proteins expressed in platelets

We extracted from the results of Sun et al. the statistics summarising the univariate associations of rs6993770 with 1456 proteins showing strong evidence for expression in MKs (RNA-seq log_2_FPKM > 1)^30^. The estimated effect size corresponding to 215 of these proteins was sufficiently large to correspond to an unadjusted *P*-value < 1 × 10^−3^. 44 of these were localised to α-granules, of which 40 had negative estimated T-allelic effect sizes, while 171 were not localised to α-granules, of which 101 had negative T-allelic effect sizes. We performed a Fisher’s exact test for the corresponding 2×2 table.

Using a dataset in which each row corresponded to one of 1456 plasma proteins studied by Sun et al. and expressed in MKs (log_2_FPKM > 1), we performed a linear regression of the estimated effect size of rs6993770-T on a dummy variable indicating α-granule localisation while adjusting for the abundance of the mRNA transcripts for the corresponding genes in MKs.

### Colocalisation with disease risk variants

A catalogue of summary statistics from GWAS of 15 complex diseases was collated following a literature search. The diseases considered were: allergic disease^85^, Alzheimer’s disease^86^, asthma^87^, coeliac disease^88,89^, coronary artery disease^90^, Crohn’s disease^91,92^, eczema^93^, hayfever or rhinitis^85^, inflammatory bowel disease^91,92^, multiple sclerosis^94–96^, primary biliary cirrhosis^97,98^, primary sclerosing cholangitis^99^, systemic lupus erythematosus^100^, type 1 diabetes^101^ and ulcerative colitis^91^. We performed colocalisation to identify variants associated with ncCBC traits and disease risk, following the procedure described for eQTL, but without regressing out secondary signals (Supplementary Data 8).

### *ZFPM2* knock-out

*ZFPM2* knockout cell lines were obtained from human iPSCs (HPSI1113i-qolg_3 HipSci cell line) by CRISPR/Cas9 genome editing. The sgRNA (5’ GA GTC GAC AGC AAC TTC CAG 3’) was cloned into the lentiGuide-Puro (Addgene plasmid # 52963^102^). The sgRNA and Cas9 plasmids were co-transfected into a human iPSC line using the Lonza Nucleofector™ and the Human Stem Cell NucleofectorTM Kit 1 (Lonza, VPH-5012). Nucleofected cells were single cell sorted and knock-out variants were confirmed by Sanger sequencing (Supplementary Fig. 12).

### In vitro differentiation of MKs

MKs were differentiated from human iPSCs (HPSI1113i-qolg_3 HipSci cell line, wild-type and ZFPM2 knockout) using the protocol described in Moreau et al.^32^. Briefly, cells were trypsinized and seeded with 100,000 cells per well. The next day, cells were infected with lentiviruses encoding the GATA1, FLI1 and TAL1 transcription factors (Vectalis). The first two days after infection, cells were cultured with BMP4 (BioTechne) and FGF2 (Wellcome—MRC Cambridge Stem Cell Institute, Tissue Culture facility) to induce mesoderm. Eighteen days were allowed for the differentiation and maturation of MKs, during which flow-cytometry experiments were used to monitor the expression of CD41a (BD, cat 559777) and CD42b (BD, 555473) as measures of maturation. Three technical replicates were nested within each of three biological replicates: two knockouts and one wild-type.

### Reporting summary

Further information on research design is available in the Nature Portfolio Reporting Summary linked to this article.

### Supplementary information

Supplementary Information

Description of Additional Supplementary Files

Supplementary Data 1–8

Reporting Summary

## Data Availability

For ethical and legal reasons access to INTERVAL data are subject to controls. Bona fide scientists can seek access to relevant de-identified individual participant data—including genetic, haematology analyser and proteomic data—and a copy of the trial’s data dictionary by applying to the INTERVAL Data Access Committee using the email address helpdesk@intervalstudy.org.uk. The INTERVAL Data Access Committee (supplemented, when required, by expertise from additional external scientists) meets several times a year to review applications according to the usual academic criteria of scientific validity and feasibility. Following approval by the INTERVAL Data Access Committee, a material transfer or research collaboration agreement will be agreed and signed with the applicants. Applicants might be requested to provide reimbursement of data management or preparation costs, as the INTERVAL trial is no longer in receipt of funding. Applicants will be required to provide updates to the INTERVAL Data Access Committee on their use of the INTERVAL trial data, including provision of copies of any publications. Applicants will be required to adhere in publications with the INTERVAL trial’s policy for acknowledgment of the trial’s funders, stakeholders, and scientific or technical contributors. The GRCh37 genome reference build is available for download from https://grch37.ensembl.org/info/data/ftp/index.html. Genomewide summary statistics may be downloaded by anonymous ftp from ftp://ftp.sanger.ac.uk/pub/project/humgen/summary_statistics/sysmex_blood_cell_genetics. The data from Ulirsch et al.^29^ are available from https://github.com/caleblareau/singlecell_bloodtraits/, from the Gene Expression Omnibus (GEO) under accession GSE119453 and from the Sequence Read Archive (SRA) under accession PRJNA491478. Other MK epigenetic data were generated by the BLUEPRINT project and are available in the EGA dataset EGAD00001001871.

## Code availability

The R code used for the association analysis is available in the git repository: https://github.com/ParsaAkbari/UKBB500K-Conditional-Analysis.
