# Supplementary material for: A genome-wide association study of blood cell morphology identifies cellular proteins implicated in disease aetiology
Source: Nat Commun. 2023 Aug 18;14:5023. doi: 10.1038/s41467-023-40679-y (PMC10439125; doi:10.1038/s41467-023-40679-y)
Supplement: Supplementary file 1 — Supplementary Information [file 41467_2023_40679_MOESM1_ESM.pdf]

# Supplementary Information

Akbari et al., A genome-wide association study of blood cell morphology identifies cellular proteins implicated in disease aetiology

## Contents


Supplementary Fig. 1 ........ 2
Supplementary Fig. 2 ........ 13
Supplementary Fig. 3 ........ 24
Supplementary Fig. 4 ........ 35
Supplementary Fig. 5 ........ 36
Supplementary Fig. 6 ........ 37
Supplementary Fig. 7 ........ 38
Supplementary Fig. 8 ........ 39
Supplementary Fig. 9 ........ 39
Supplementary Fig. 10 ........ 40
Supplementary Fig. 11 ........ 41
Supplementary Fig. 12 ........ 42
Supplementary References ........ 43

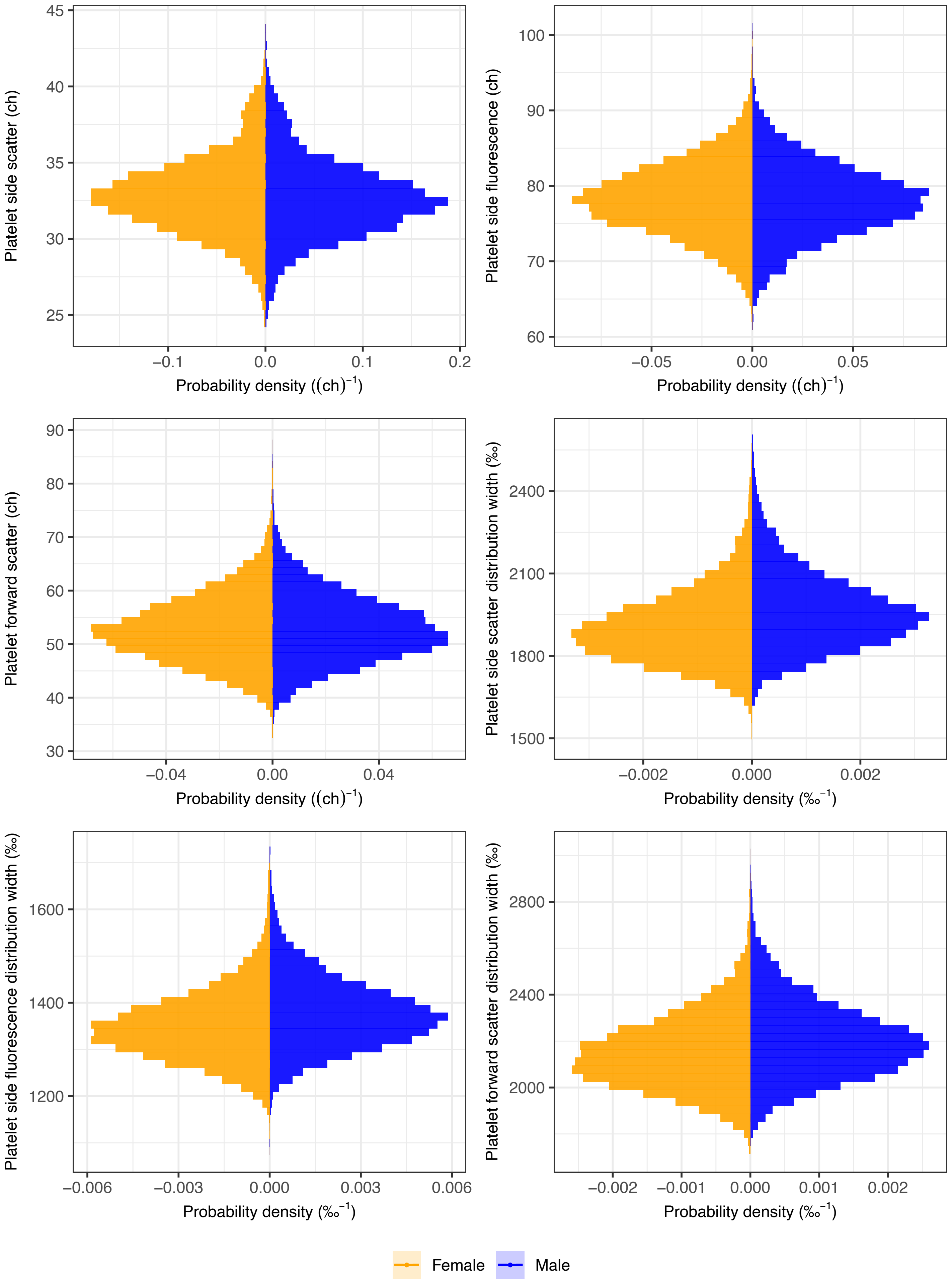

Platelet side scatter (ch)
45
40
35
30
25
−0.1
0.0
0.1
0.2
Probability density ((ch)$^{-1}$)
Platelet side fluorescence (ch)
100
90
80
70
60
−0.05
0.00
0.05
Probability density ((ch)$^{-1}$)
Platelet forward scatter (ch)
90
80
70
60
50
40
30
−0.04
0.00
0.04
Probability density ((ch)$^{-1}$)
Platelet side scatter distribution width (‰)
2400
2100
1800
1500
−0.002
0.000
0.002
Probability density (‰$^{-1}$)
Platelet side fluorescence distribution width (‰)
1600
1400
1200
−0.006
−0.003
0.000
0.003
0.006
Probability density (‰$^{-1}$)
Platelet forward scatter distribution width (‰)
2800
2400
2000
−0.002
−0.001
0.000
0.001
0.002
Probability density (‰$^{-1}$)
Female
Male

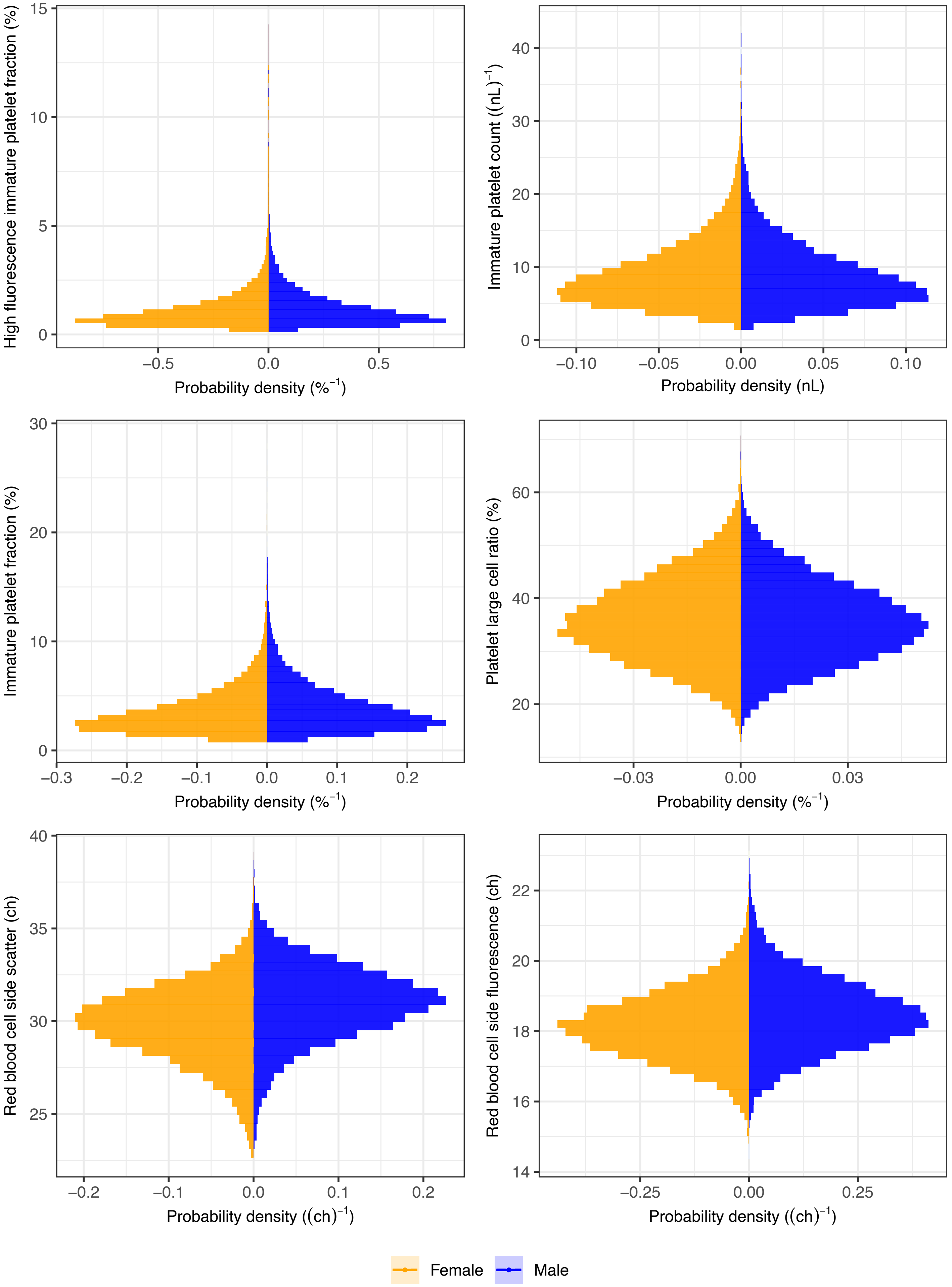

High fluorescence immature platelet fraction (%)
Probability density (%$^{-1}$)
Immature platelet count ((nL)$^{-1}$)
Probability density (nL)
Immature platelet fraction (%)
Probability density (%$^{-1}$)
Platelet large cell ratio (%)
Probability density (%$^{-1}$)
Red blood cell side scatter (ch)
Probability density ((ch)$^{-1}$)
Red blood cell side fluorescence (ch)
Probability density ((ch)$^{-1}$)
Female
Male

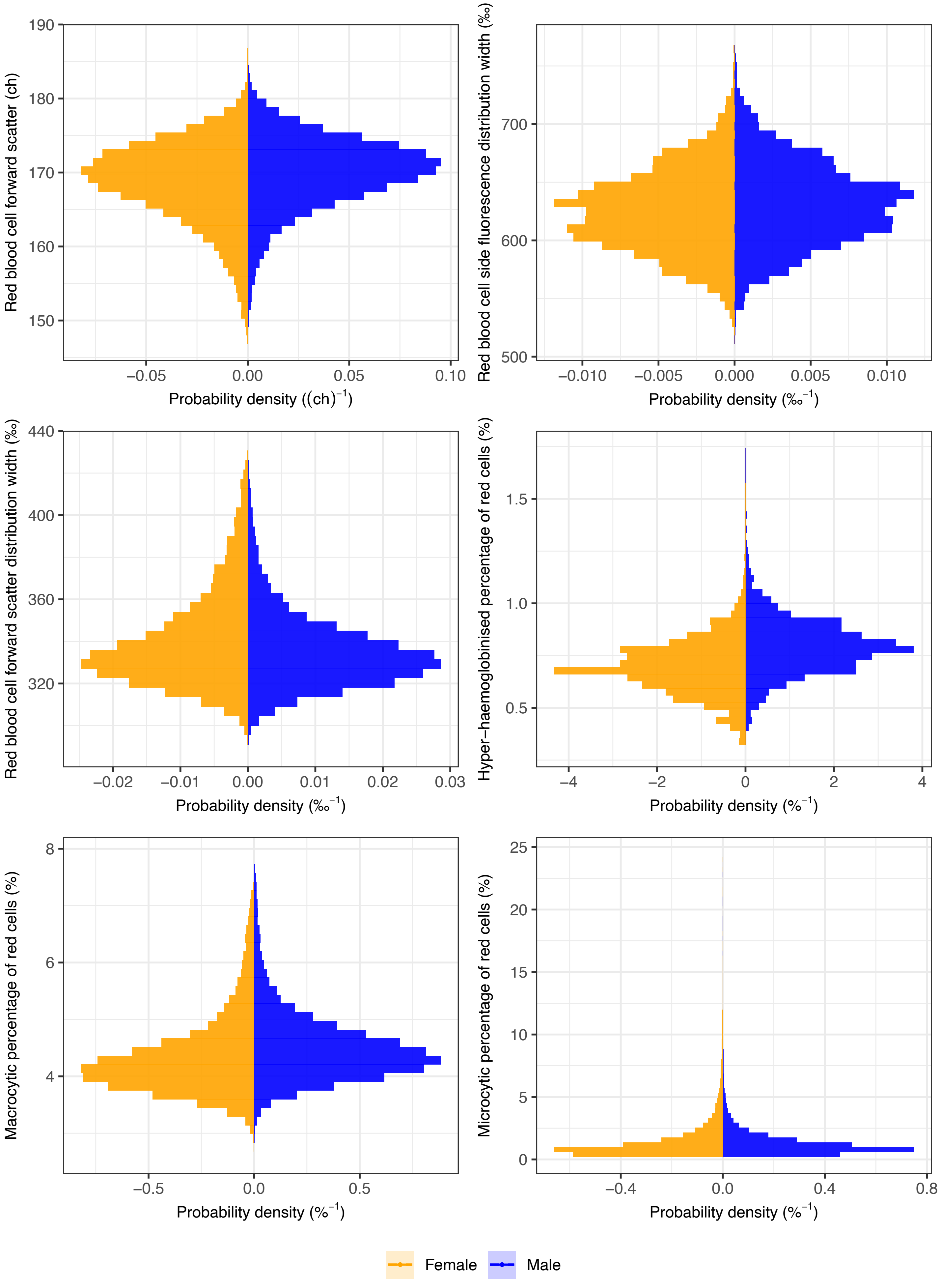
Red blood cell forward scatter (ch)
Probability density ((ch)$^{-1}$)
Red blood cell side fluorescence distribution width (‰)
Probability density (‰$^{-1}$)
Red blood cell forward scatter distribution width (‰)
Probability density (‰$^{-1}$)
Hyper−haemoglobinised percentage of red cells (%)
Probability density (%$^{-1}$)
Macrocytic percentage of red cells (%)
Probability density (%$^{-1}$)
Microcytic percentage of red cells (%)
Probability density (%$^{-1}$)
Female
Male

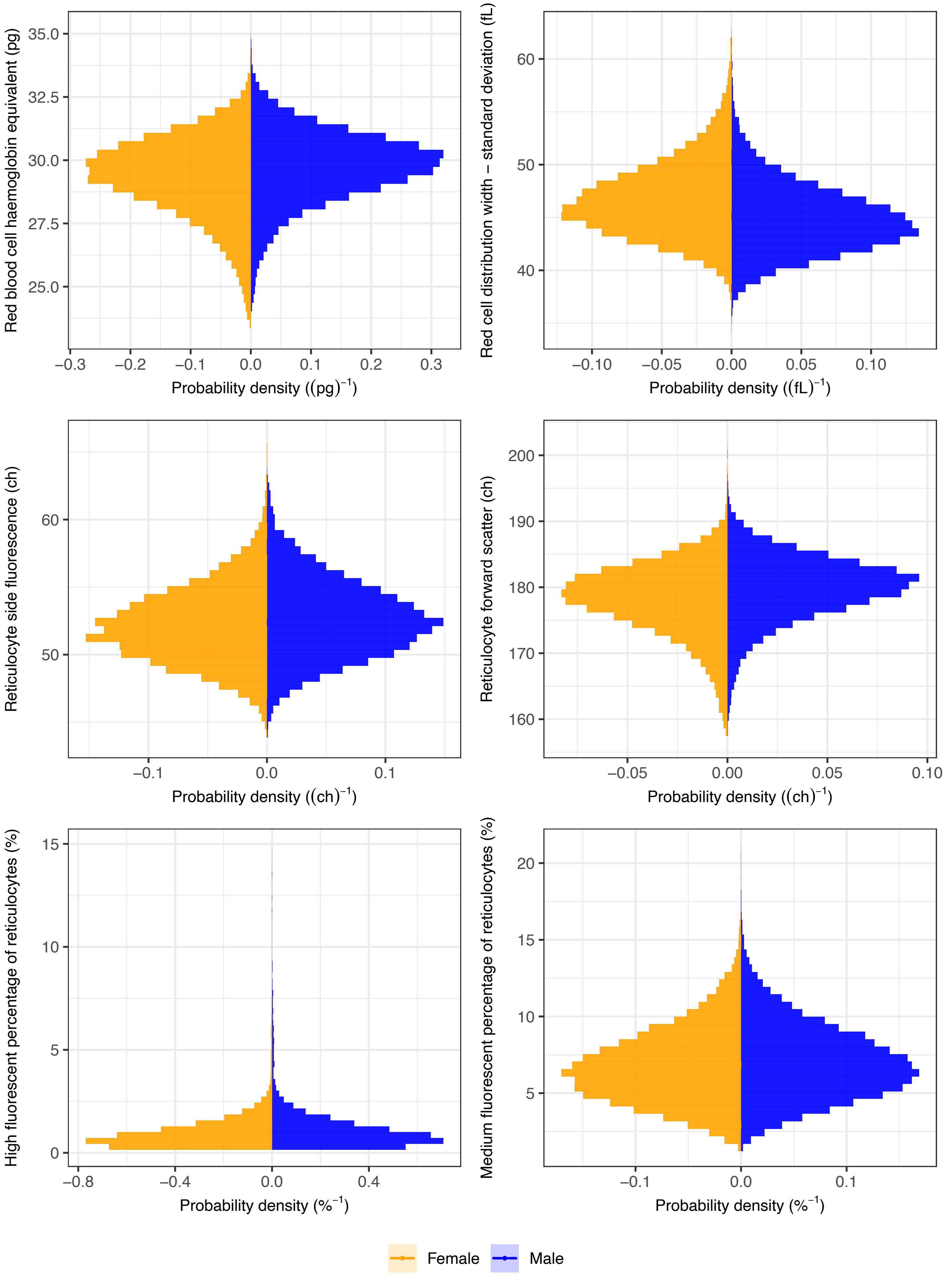
Red blood cell haemoglobin equivalent (pg)
Probability density ((pg)$^{-1}$)
Red cell distribution width – standard deviation (fL)
Probability density ((fL)$^{-1}$)
Reticulocyte side fluorescence (ch)
Probability density ((ch)$^{-1}$)
Reticulocyte forward scatter (ch)
Probability density ((ch)$^{-1}$)
High fluorescent percentage of reticulocytes (%)
Probability density (%$^{-1}$)
Medium fluorescent percentage of reticulocytes (%)
Probability density (%$^{-1}$)
Female
Male

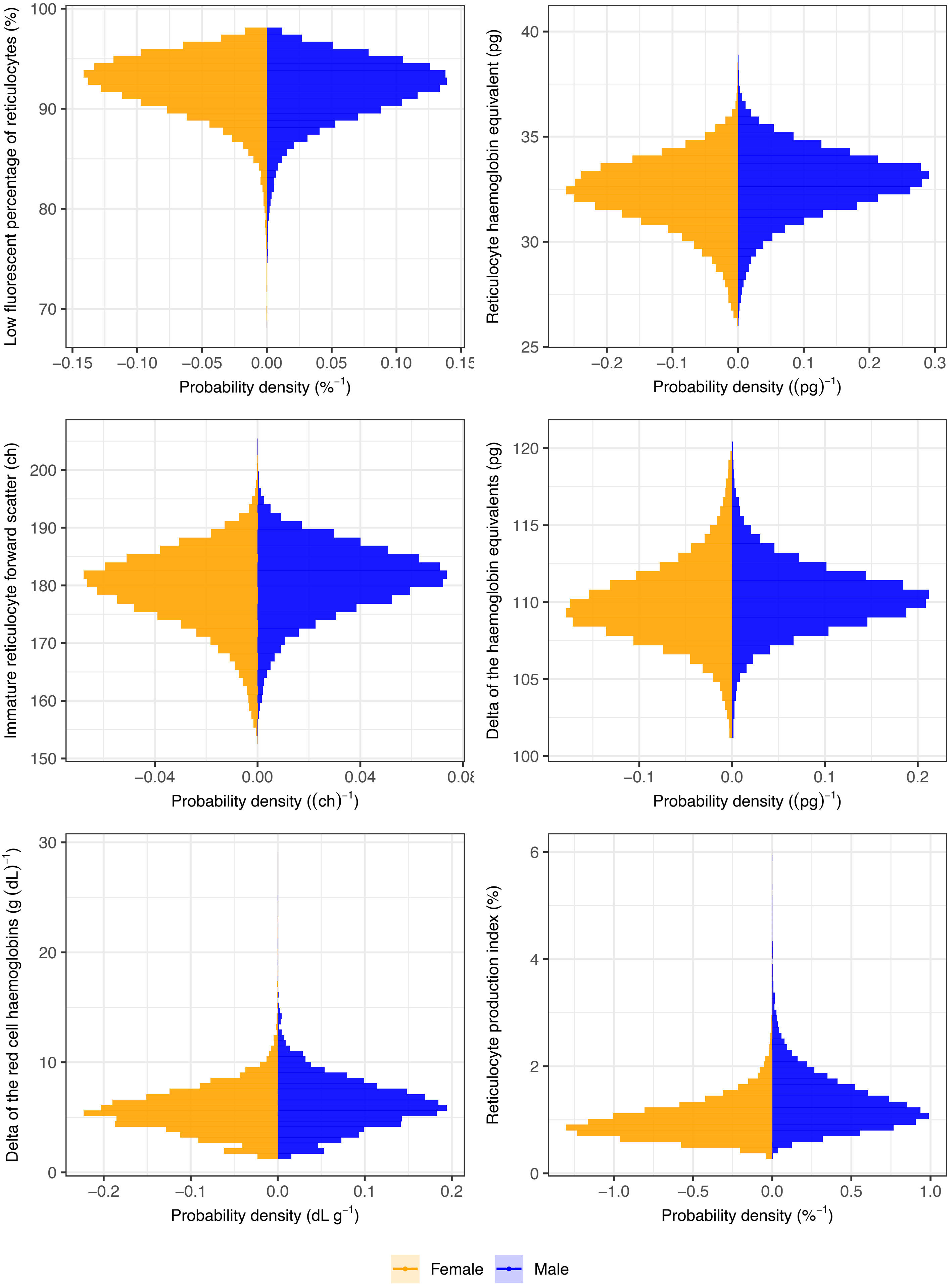

Low fluorescent percentage of reticulocytes (%)
Probability density (%$^{-1}$)
Reticulocyte haemoglobin equivalent (pg)
Probability density ((pg)$^{-1}$)
Immature reticulocyte forward scatter (ch)
Probability density ((ch)$^{-1}$)
Delta of the haemoglobin equivalents (pg)
Probability density ((pg)$^{-1}$)
Delta of the red cell haemoglobins (g (dL)$^{-1}$)
Probability density (dL g$^{-1}$)
Reticulocyte production index (%)
Probability density (%$^{-1}$)
Female
Male

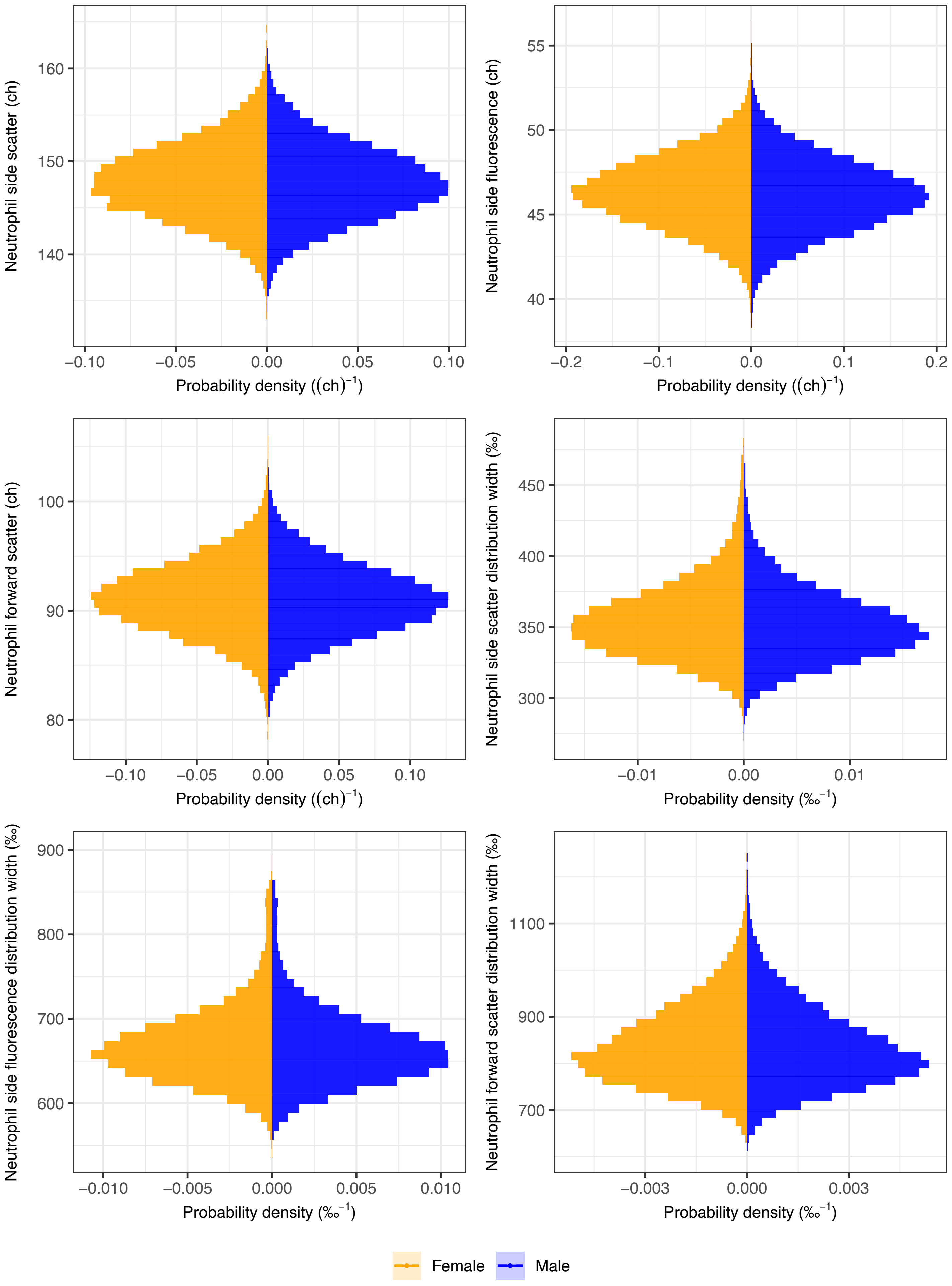
Neutrophil side scatter (ch)
Probability density ((ch)$^{-1}$)
Neutrophil side fluorescence (ch)
Probability density ((ch)$^{-1}$)
Neutrophil forward scatter (ch)
Probability density ((ch)$^{-1}$)
Neutrophil side scatter distribution width (‰)
Probability density (‰$^{-1}$)
Neutrophil side fluorescence distribution width (‰)
Probability density (‰$^{-1}$)
Neutrophil forward scatter distribution width (‰)
Probability density (‰$^{-1}$)
Female
Male

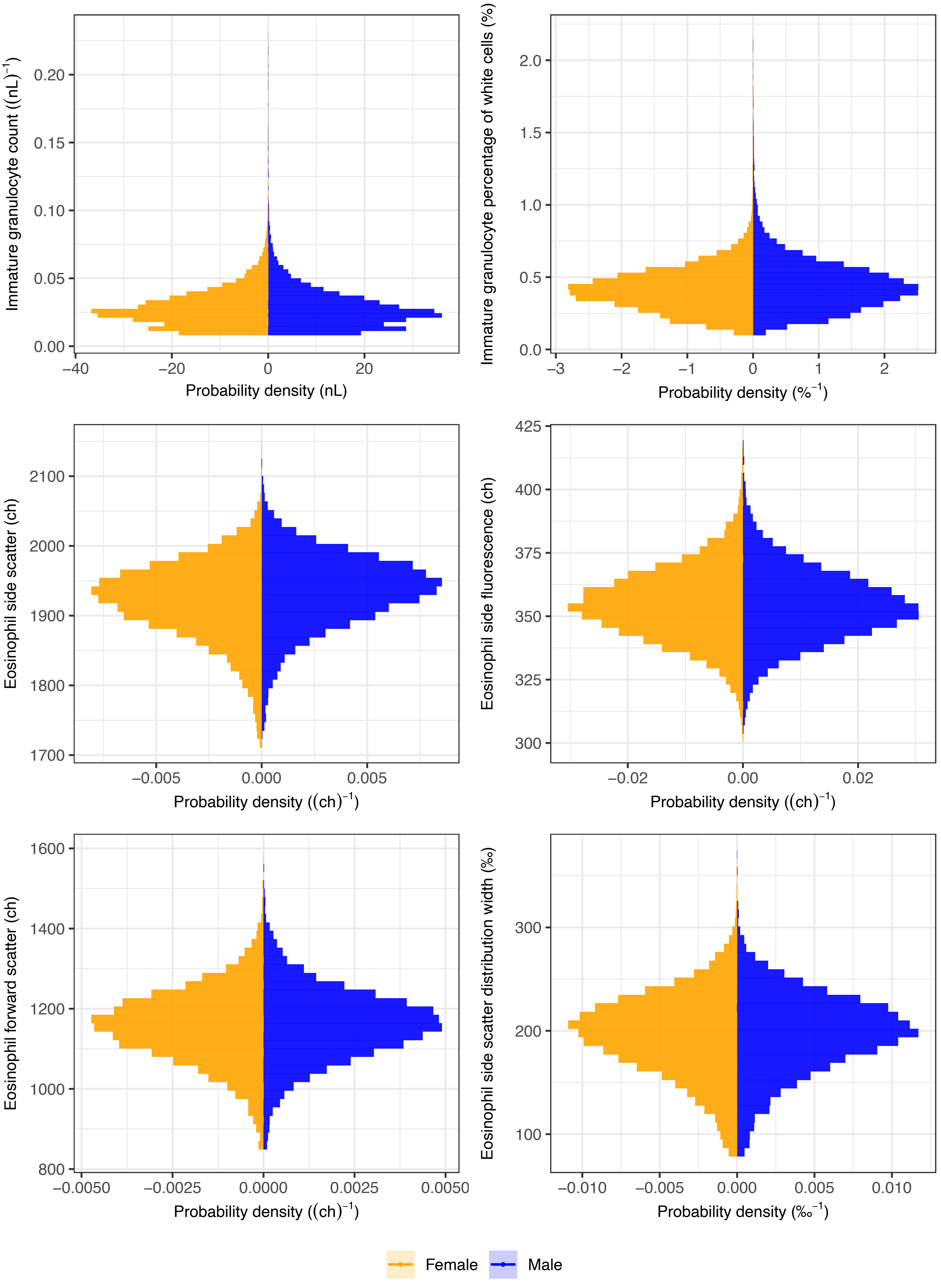
Immature granulocyte count ((nL)^-1)
0.20
0.15
0.10
0.05
0.00
−40
−20
0
20
Probability density (nL)
Immature granulocyte percentage of white cells (%)
2.0
1.5
1.0
0.5
0.0
−3
−2
−1
0
1
2
Probability density (%^-1)
Eosinophil side scatter (ch)
2100
2000
1900
1800
1700
−0.005
0.000
0.005
Probability density ((ch)^-1)
Eosinophil side fluorescence (ch)
425
400
375
350
325
300
−0.02
0.00
0.02
Probability density ((ch)^-1)
Eosinophil forward scatter (ch)
1600
1400
1200
1000
800
−0.0050
−0.0025
0.0000
0.0025
0.0050
Probability density ((ch)^-1)
Eosinophil side scatter distribution width (‰)
300
200
100
−0.010
−0.005
0.000
0.005
0.010
Probability density (‰^-1)
Female
Male

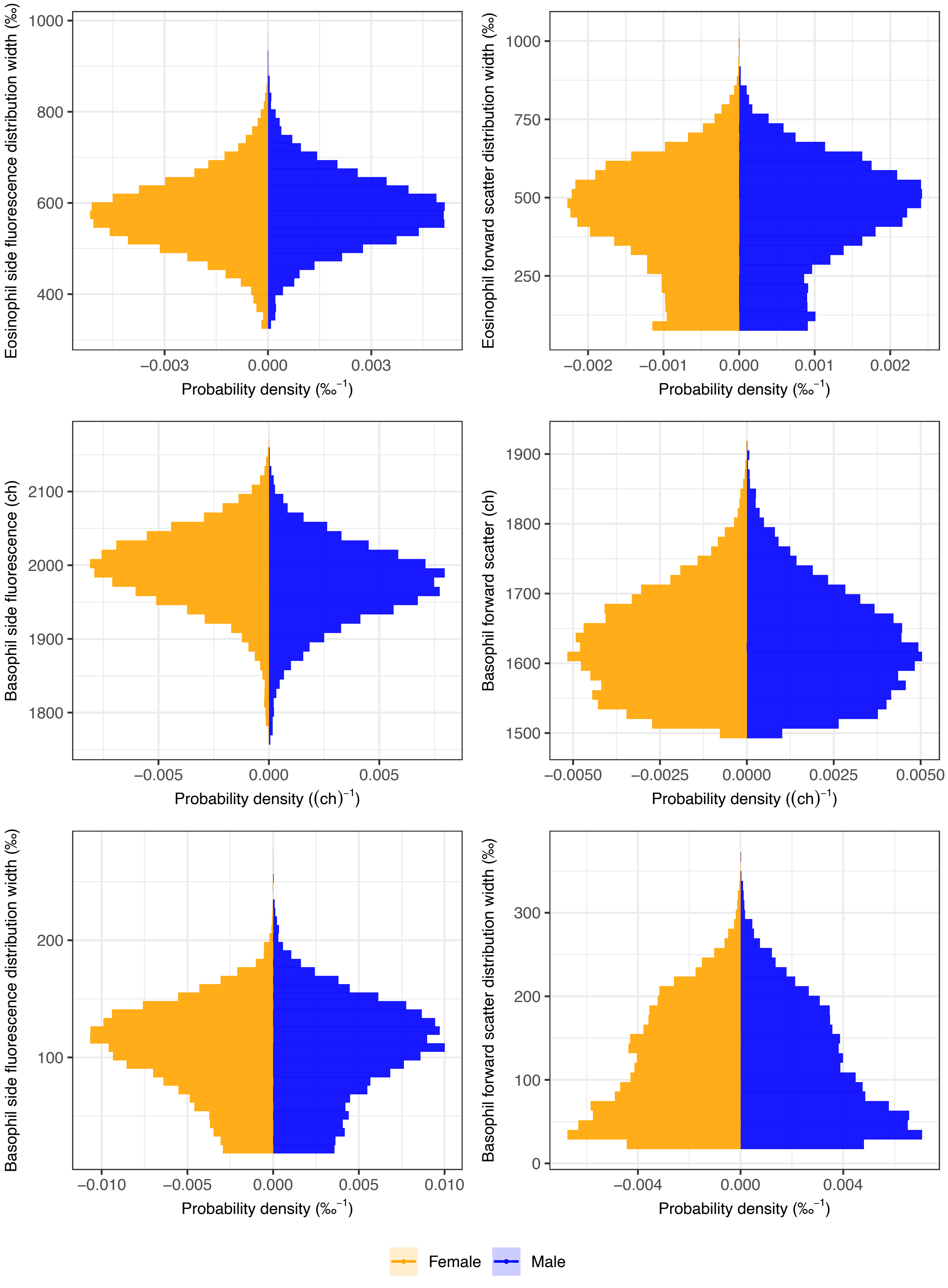
Eosinophil side fluorescence distribution width (‰)
Probability density (‰$^{-1}$)
Eosinophil forward scatter distribution width (‰)
Probability density (‰$^{-1}$)
Basophil side fluorescence (ch)
Probability density ((ch)$^{-1}$)
Basophil forward scatter (ch)
Probability density ((ch)$^{-1}$)
Basophil side fluorescence distribution width (‰)
Probability density (‰$^{-1}$)
Basophil forward scatter distribution width (‰)
Probability density (‰$^{-1}$)
Female
Male

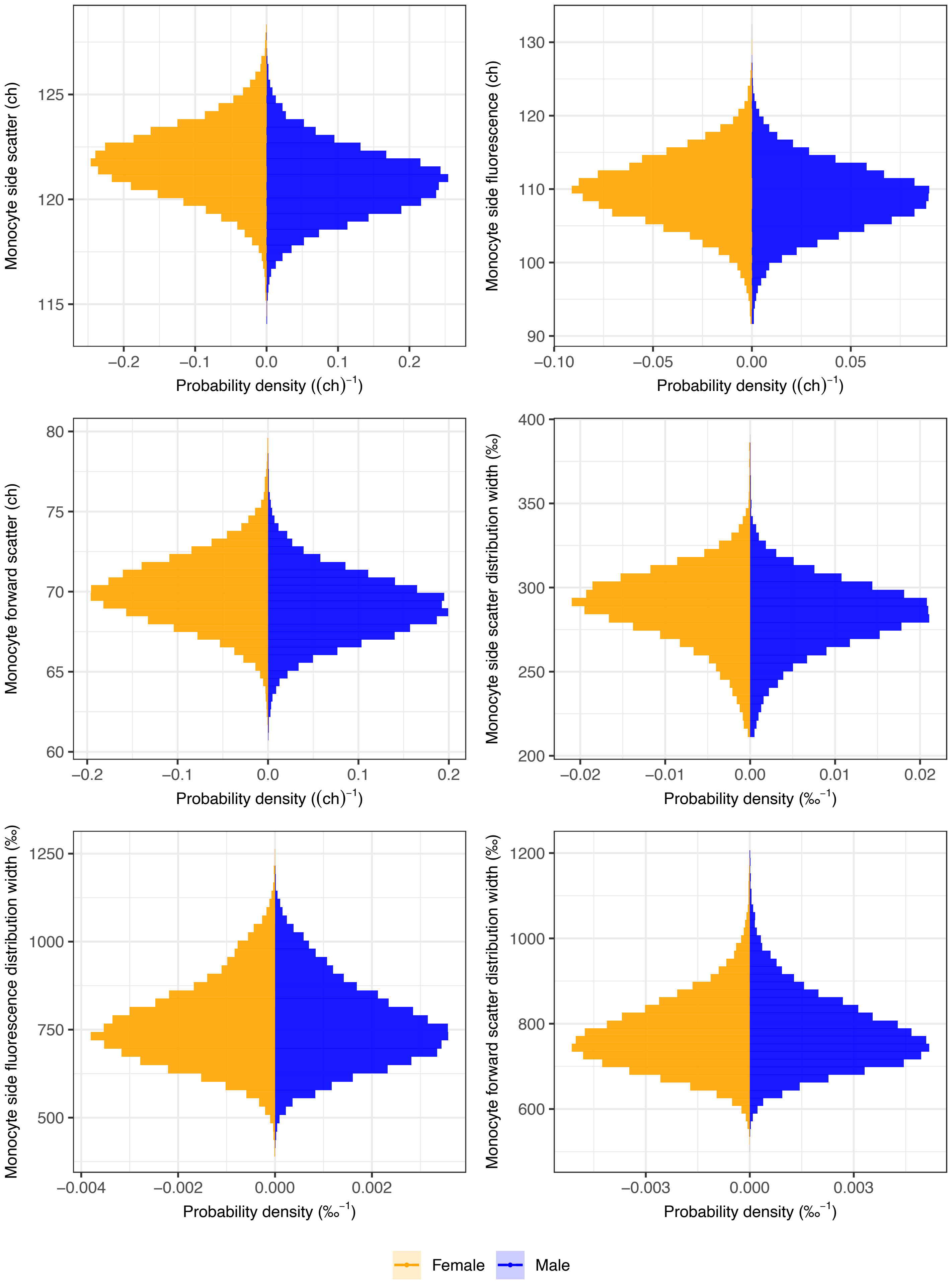
Monocyte side scatter (ch)
Probability density ((ch)$^{-1}$)
Monocyte side fluorescence (ch)
Probability density ((ch)$^{-1}$)
Monocyte forward scatter (ch)
Probability density ((ch)$^{-1}$)
Monocyte side scatter distribution width (‰)
Probability density (‰$^{-1}$)
Monocyte side fluorescence distribution width (‰)
Probability density (‰$^{-1}$)
Monocyte forward scatter distribution width (‰)
Probability density (‰$^{-1}$)
Female
Male

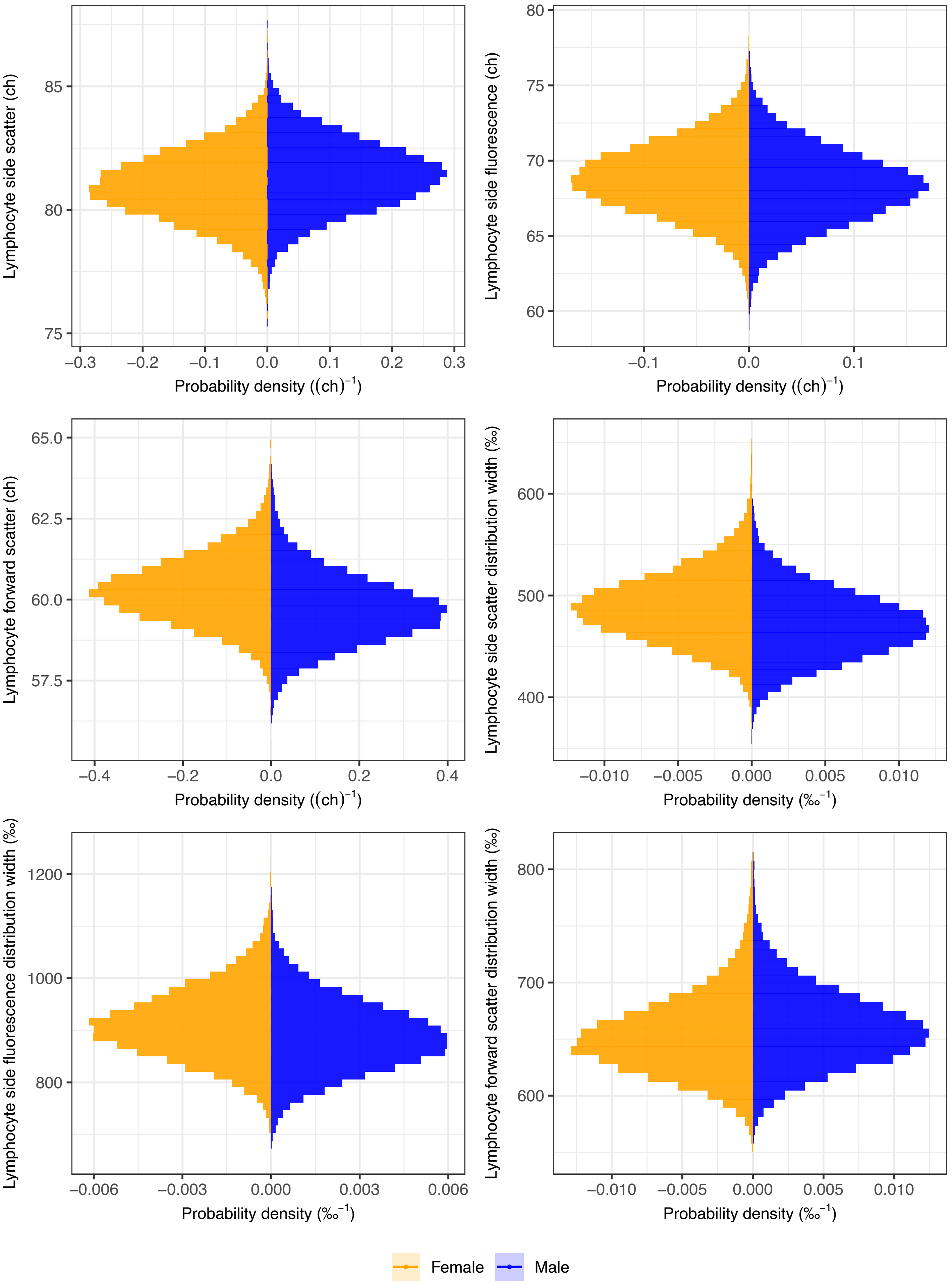
Lymphocyte side scatter (ch)
Probability density ((ch)−1)
Lymphocyte side fluorescence (ch)
Probability density ((ch)−1)
Lymphocyte forward scatter (ch)
Probability density ((ch)−1)
Lymphocyte side scatter distribution width (‰)
Probability density (‰−1)
Lymphocyte side fluorescence distribution width (‰)
Probability density (‰−1)
Lymphocyte forward scatter distribution width (‰)
Probability density (‰−1)
Female
Male

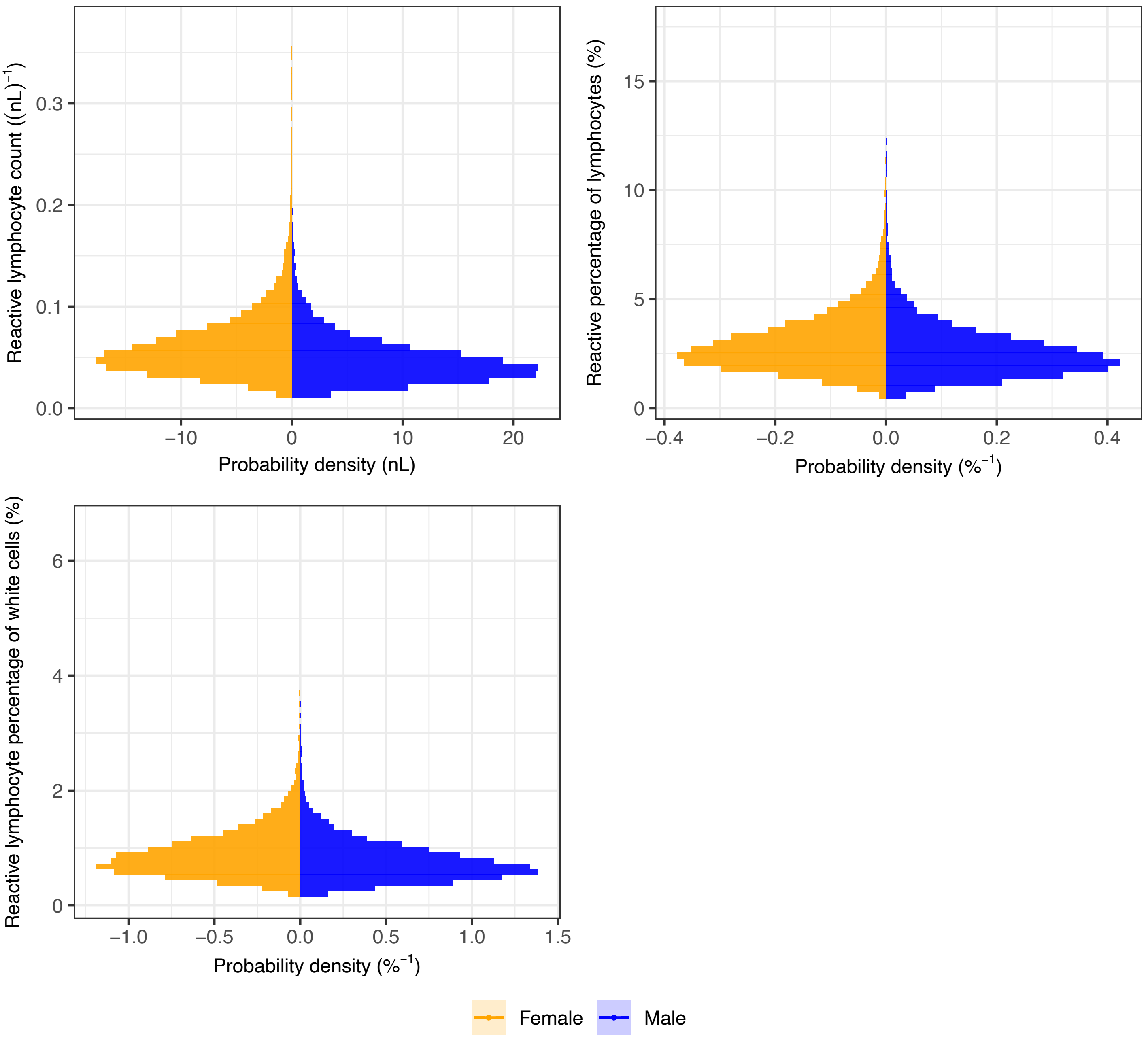


**Supplementary Fig. 1 | Histograms of technically adjusted ncCBC traits stratified by sex.** Probability density histograms of the 63 ncCBC traits stratified by sex (orange=female, blue=male). The data are from the technically adjusted traits (Methods) restricted to the participants who contribute data to the GWAS of the respective trait, the numbers of which are given in Supplementary Data 2.

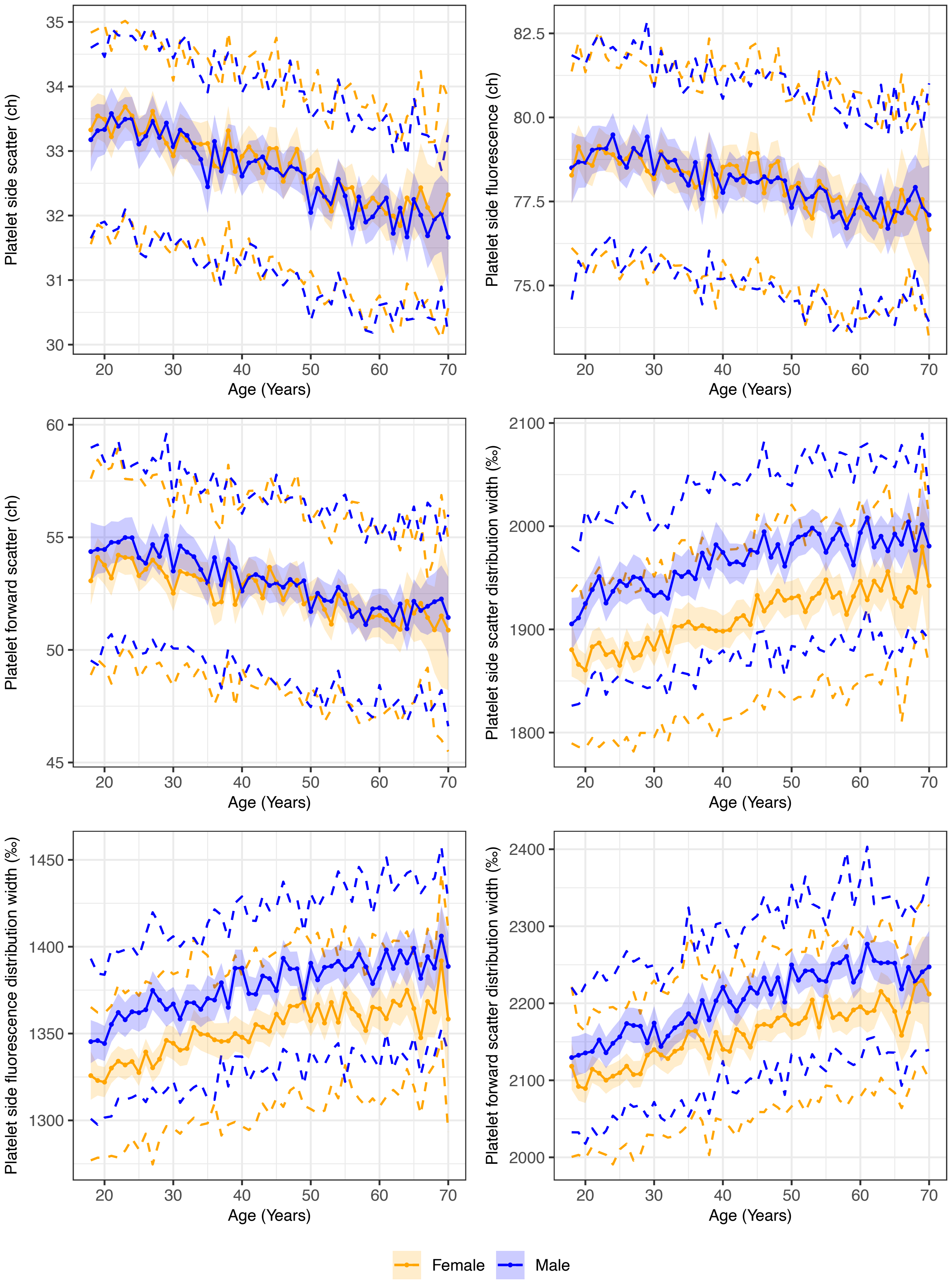

Platelet side scatter (ch)
Platelet side fluorescence (ch)
Platelet forward scatter (ch)
Platelet side scatter distribution width (‰)
Platelet side fluorescence distribution width (‰)
Platelet forward scatter distribution width (‰)
Age (Years)
Female
Male

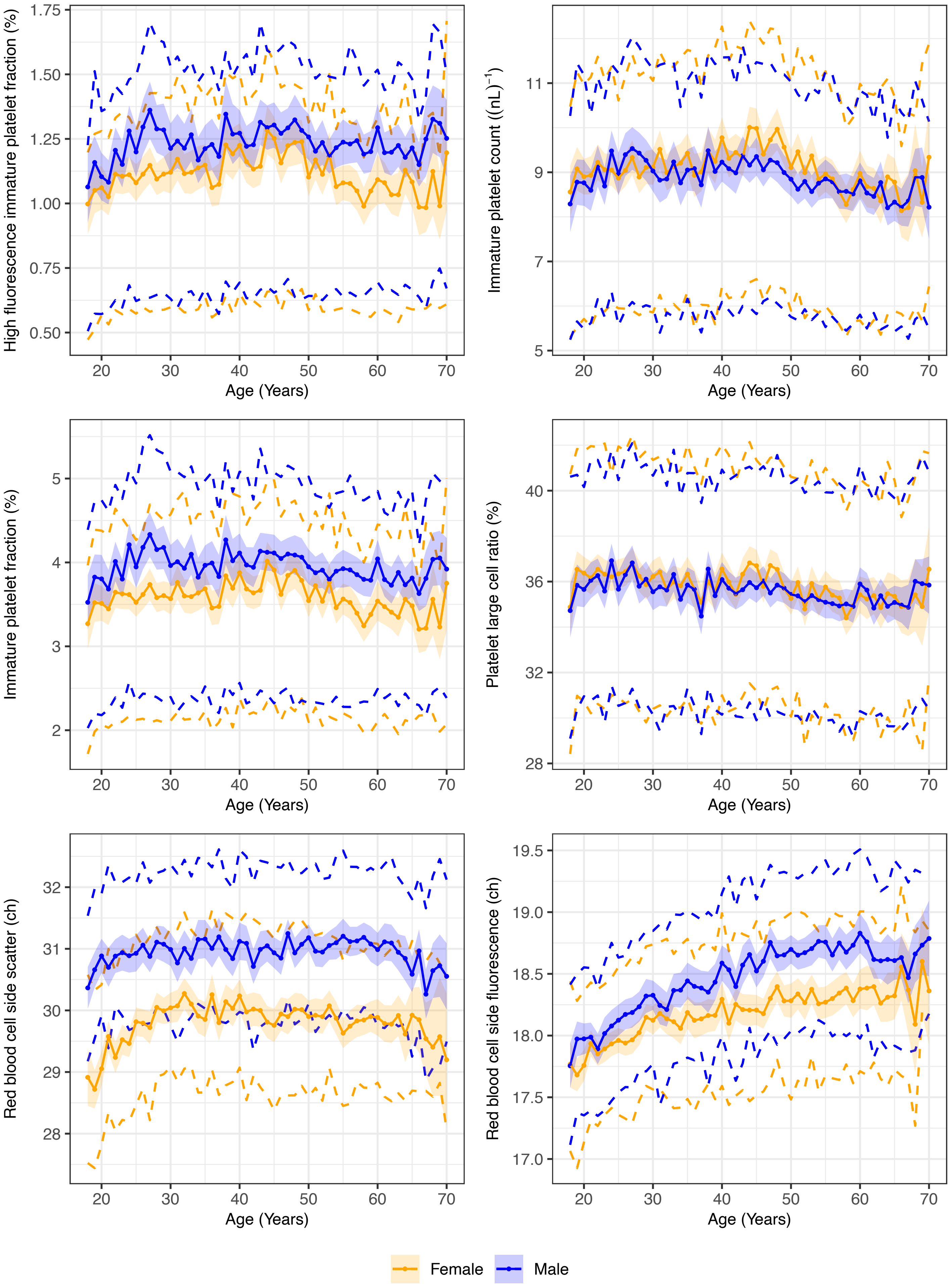

High fluorescence immature platelet fraction (%)
Immature platelet count ((nL)$^{-1}$)
Immature platelet fraction (%)
Platelet large cell ratio (%)
Red blood cell side scatter (ch)
Red blood cell side fluorescence (ch)
Age (Years)
Female
Male

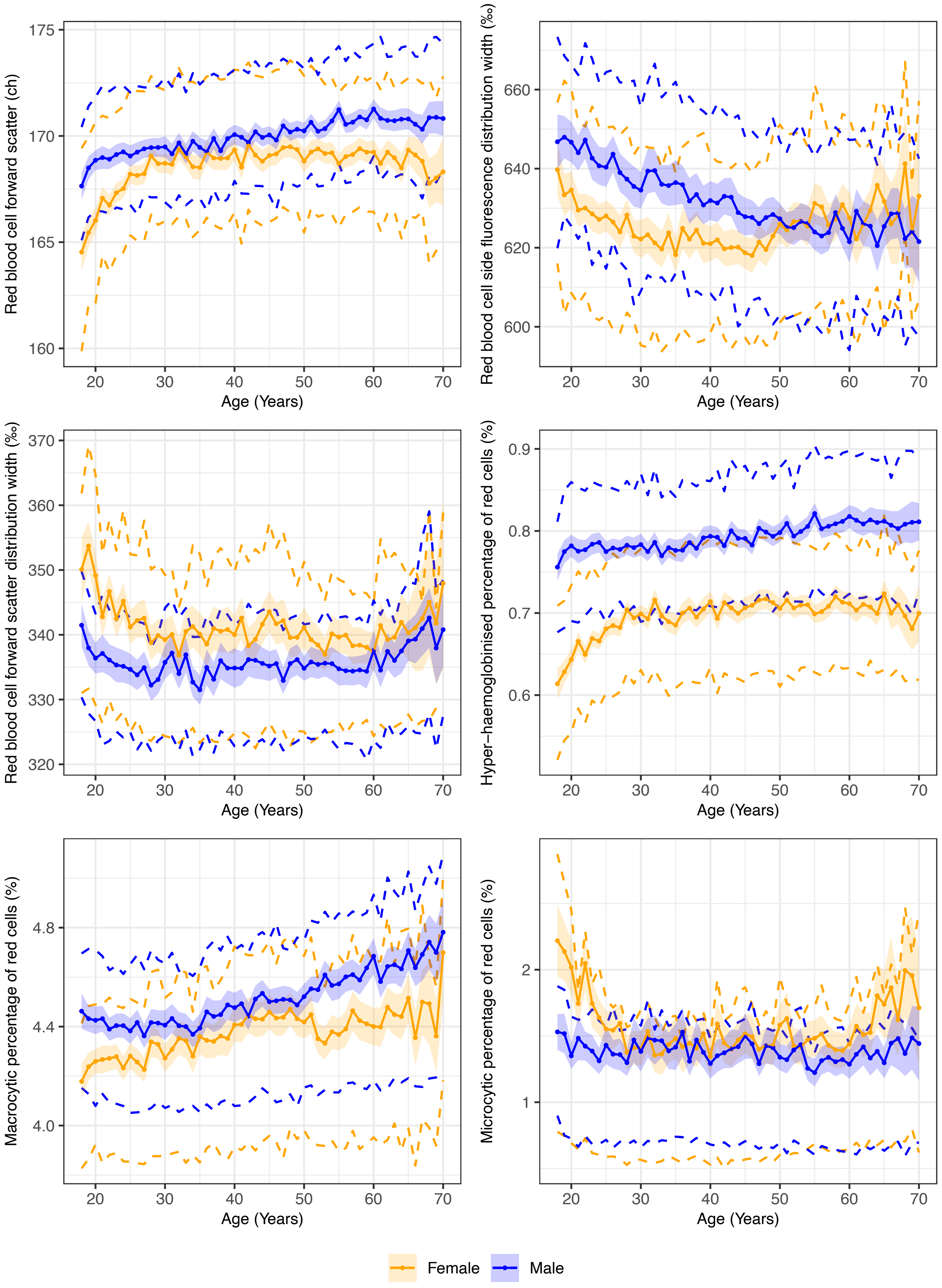
Red blood cell forward scatter (ch)
Red blood cell side fluorescence distribution width (‰)
Red blood cell forward scatter distribution width (‰)
Hyper−haemoglobinised percentage of red cells (%)
Macrocytic percentage of red cells (%)
Microcytic percentage of red cells (%)
Age (Years)
Female
Male

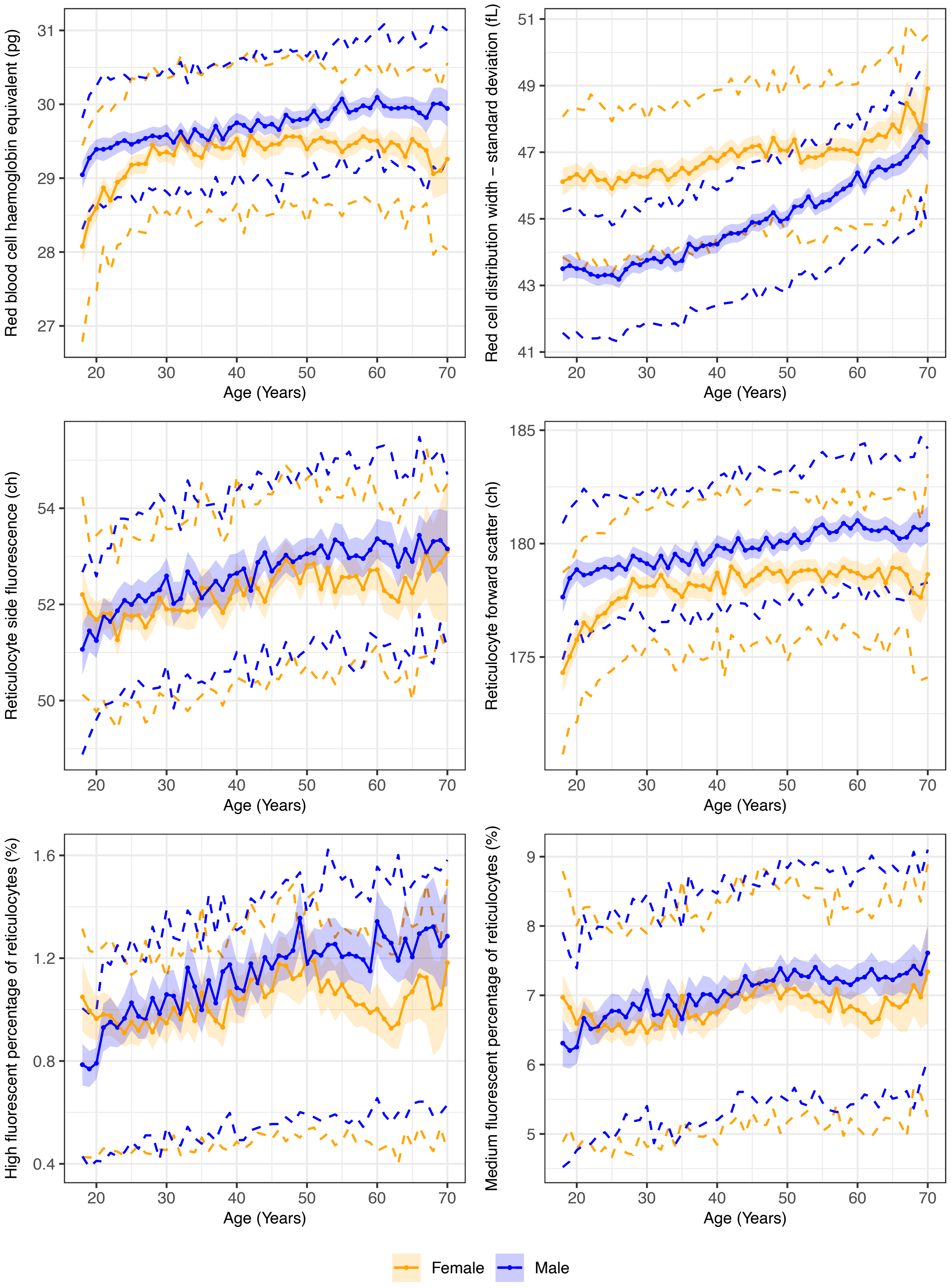

Red blood cell haemoglobin equivalent (pg)
Red cell distribution width – standard deviation (fL)
Reticulocyte side fluorescence (ch)
Reticulocyte forward scatter (ch)
High fluorescent percentage of reticulocytes (%)
Medium fluorescent percentage of reticulocytes (%)
Age (Years)
Female
Male

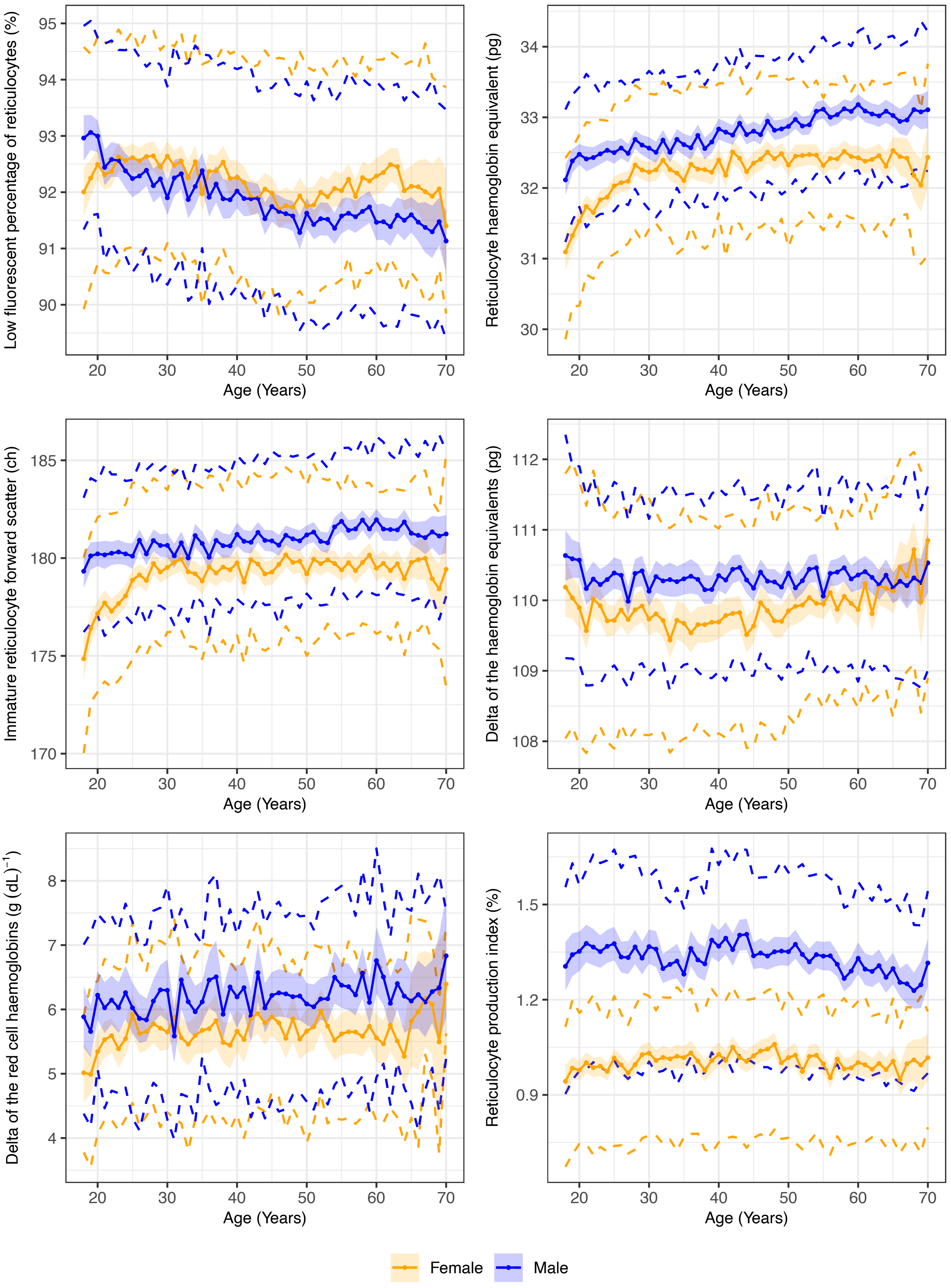

Low fluorescent percentage of reticulocytes (%)
Reticulocyte haemoglobin equivalent (pg)
Immature reticulocyte forward scatter (ch)
Delta of the haemoglobin equivalents (pg)
Delta of the red cell haemoglobins (g (dL)$^{-1}$)
Reticulocyte production index (%)
Age (Years)
Female
Male

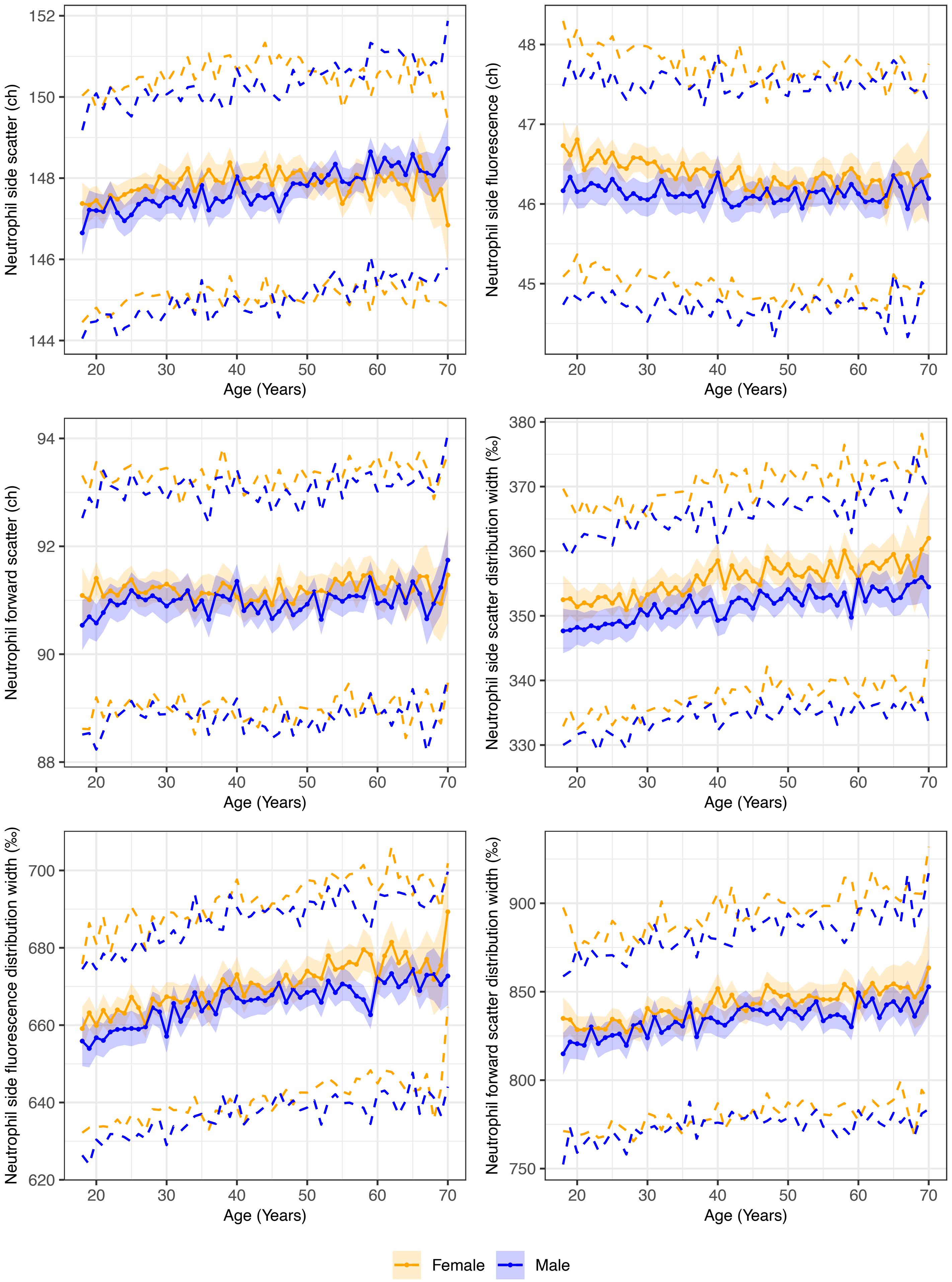

Neutrophil side scatter (ch)
Neutrophil side fluorescence (ch)
Neutrophil forward scatter (ch)
Neutrophil side scatter distribution width (‰)
Neutrophil side fluorescence distribution width (‰)
Neutrophil forward scatter distribution width (‰)
Age (Years)
Female
Male

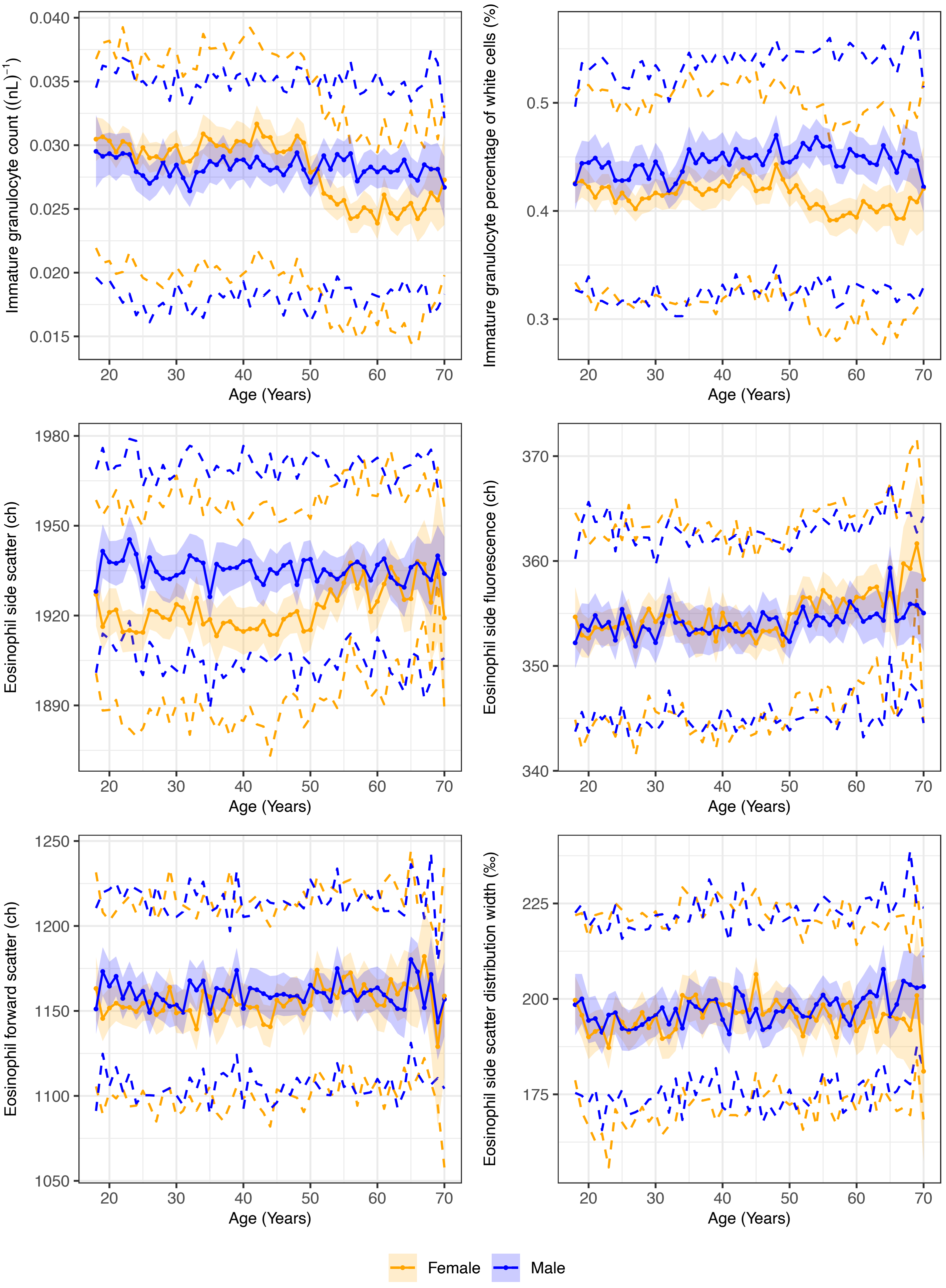
Immature granulocyte count ((nL)$^{-1}$)
Immature granulocyte percentage of white cells (%)
Eosinophil side scatter (ch)
Eosinophil side fluorescence (ch)
Eosinophil forward scatter (ch)
Eosinophil side scatter distribution width (‰)
Age (Years)
Female
Male

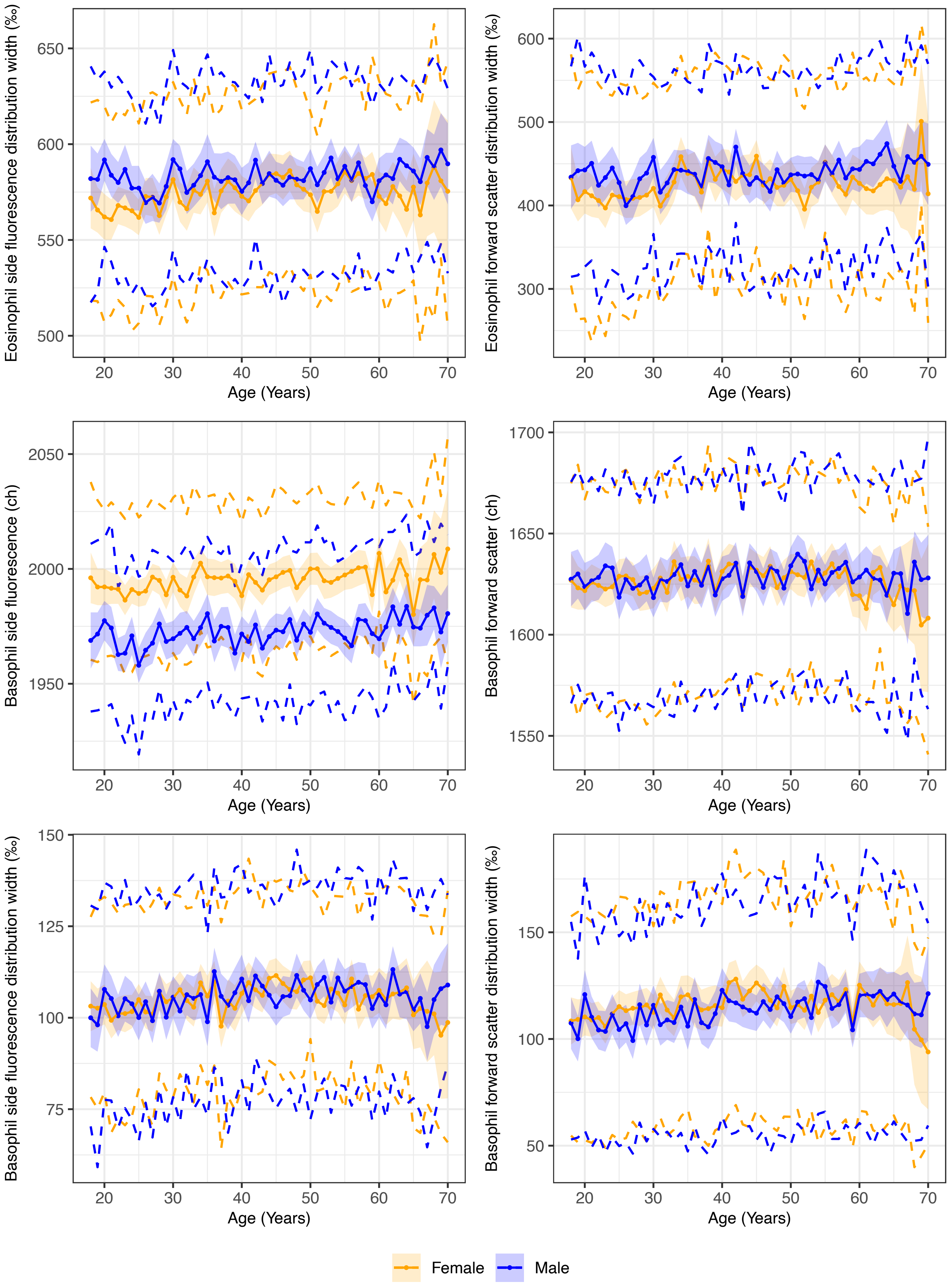
Eosinophil side fluorescence distribution width (‰)
Eosinophil forward scatter distribution width (‰)
Basophil side fluorescence (ch)
Basophil forward scatter (ch)
Basophil side fluorescence distribution width (‰)
Basophil forward scatter distribution width (‰)
Age (Years)
Female
Male

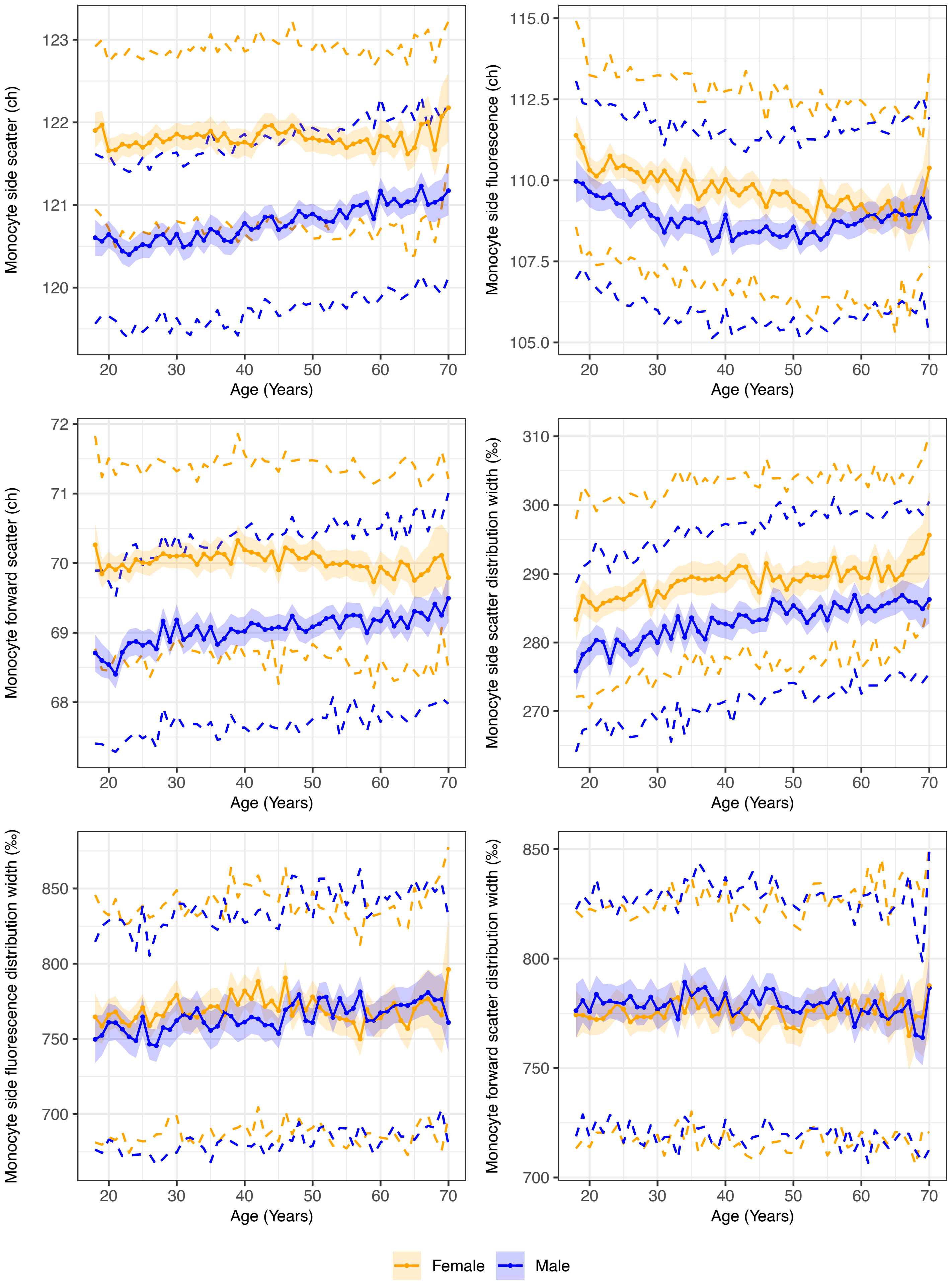
Monocyte side scatter (ch)
Monocyte side fluorescence (ch)
Monocyte forward scatter (ch)
Monocyte side scatter distribution width (‰)
Monocyte side fluorescence distribution width (‰)
Monocyte forward scatter distribution width (‰)
Age (Years)
Female
Male

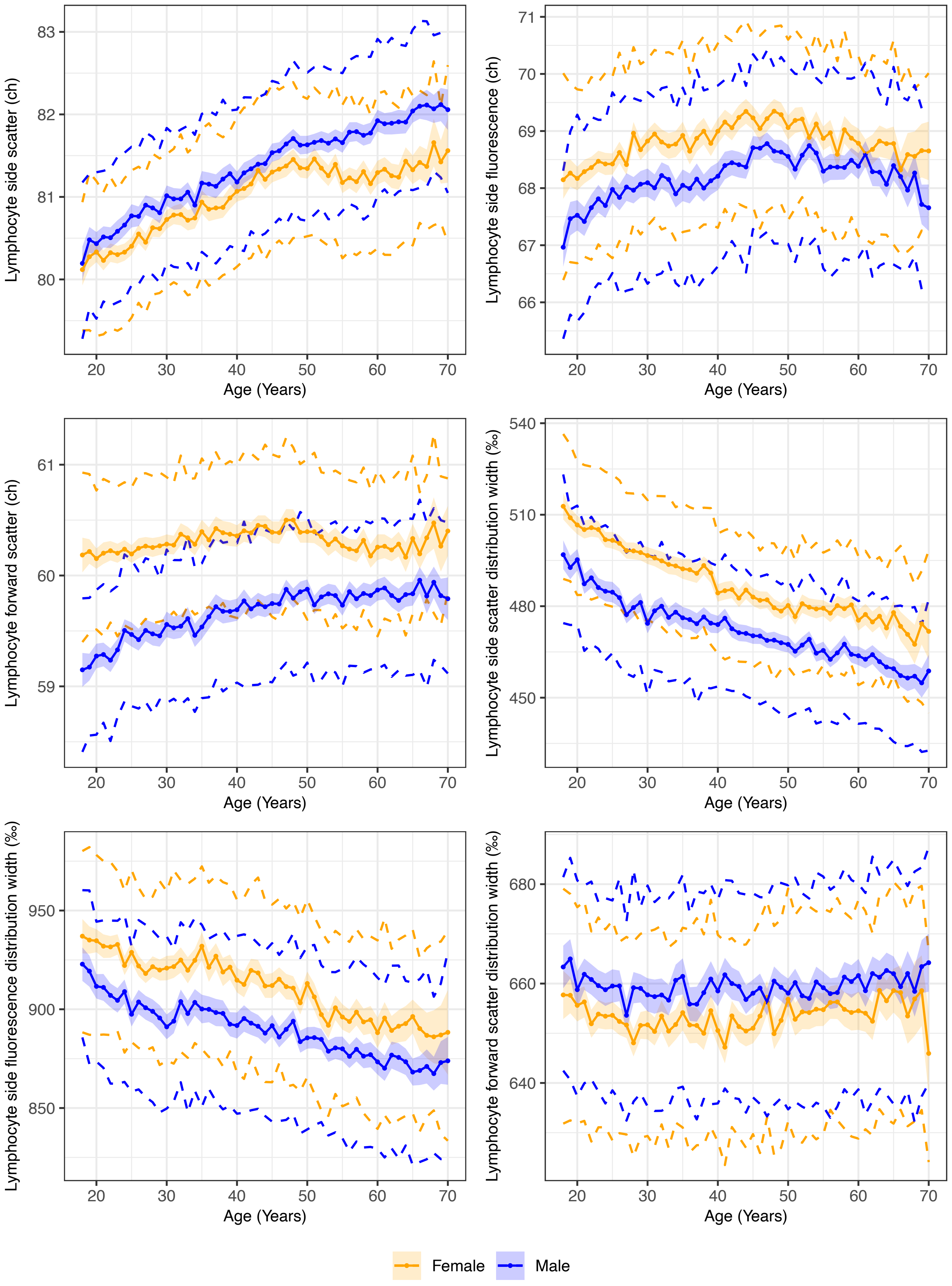

Lymphocyte side scatter (ch)
Lymphocyte side fluorescence (ch)
Lymphocyte forward scatter (ch)
Lymphocyte side scatter distribution width (‰)
Lymphocyte side fluorescence distribution width (‰)
Lymphocyte forward scatter distribution width (‰)
Age (Years)
Female
Male

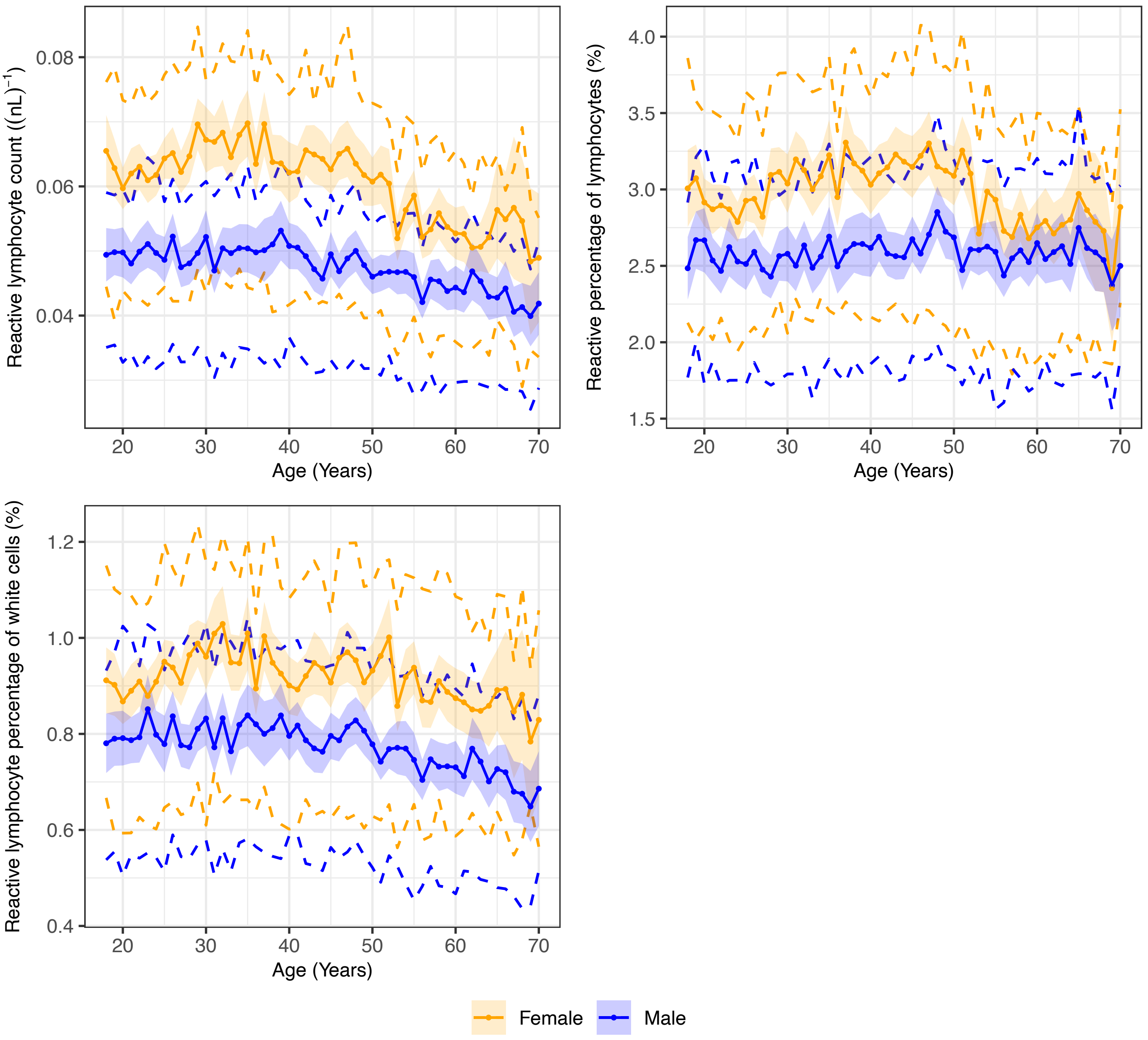


**Supplementary Fig. 2 | Covariation between technically adjusted ncCBC traits and age stratified by sex.** Covariation between the 63 nCBC traits and participant age stratified by participant sex. Parameters of the stratified trait distributions were estimated in bins corresponding to years of age. The linearly interpolated coloured points show estimates of the within strata-means and the underlying coloured ribbons show the corresponding 95% confidence intervals. The dashed lines show estimates of the upper and lower quartiles. The data are from the technically adjusted traits (Methods) restricted to the participants who contribute data to the GWAS of the respective trait, the numbers of which are given in Supplementary Data 2.

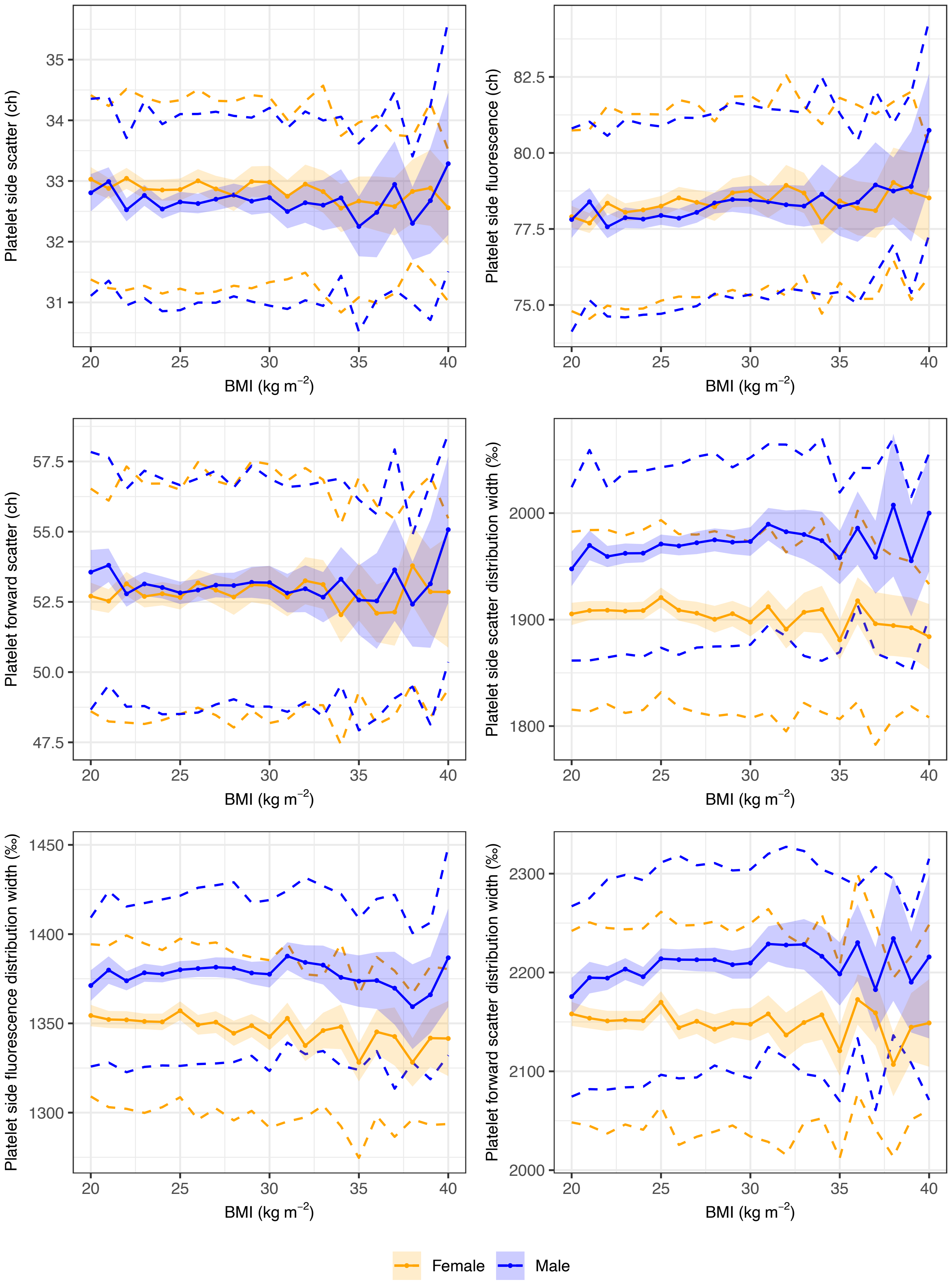
Platelet side scatter (ch)
Platelet side fluorescence (ch)
Platelet forward scatter (ch)
Platelet side scatter distribution width (‰)
Platelet side fluorescence distribution width (‰)
Platelet forward scatter distribution width (‰)
BMI (kg m$^{-2}$)
Female
Male

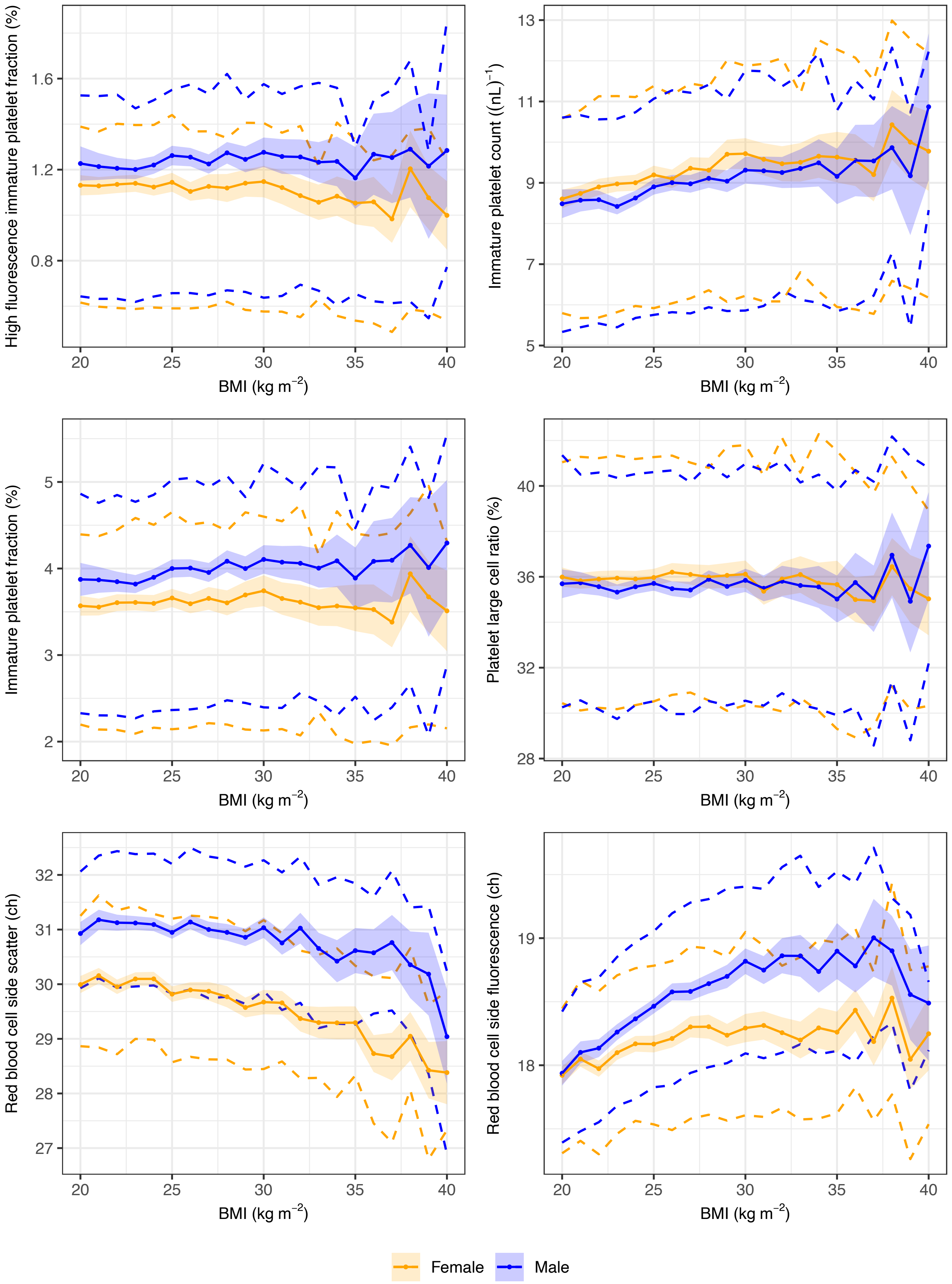
High fluorescence immature platelet fraction (%)
Immature platelet count ((nL)$^{-1}$)
Immature platelet fraction (%)
Platelet large cell ratio (%)
Red blood cell side scatter (ch)
Red blood cell side fluorescence (ch)
BMI (kg m$^{-2}$)
Female
Male

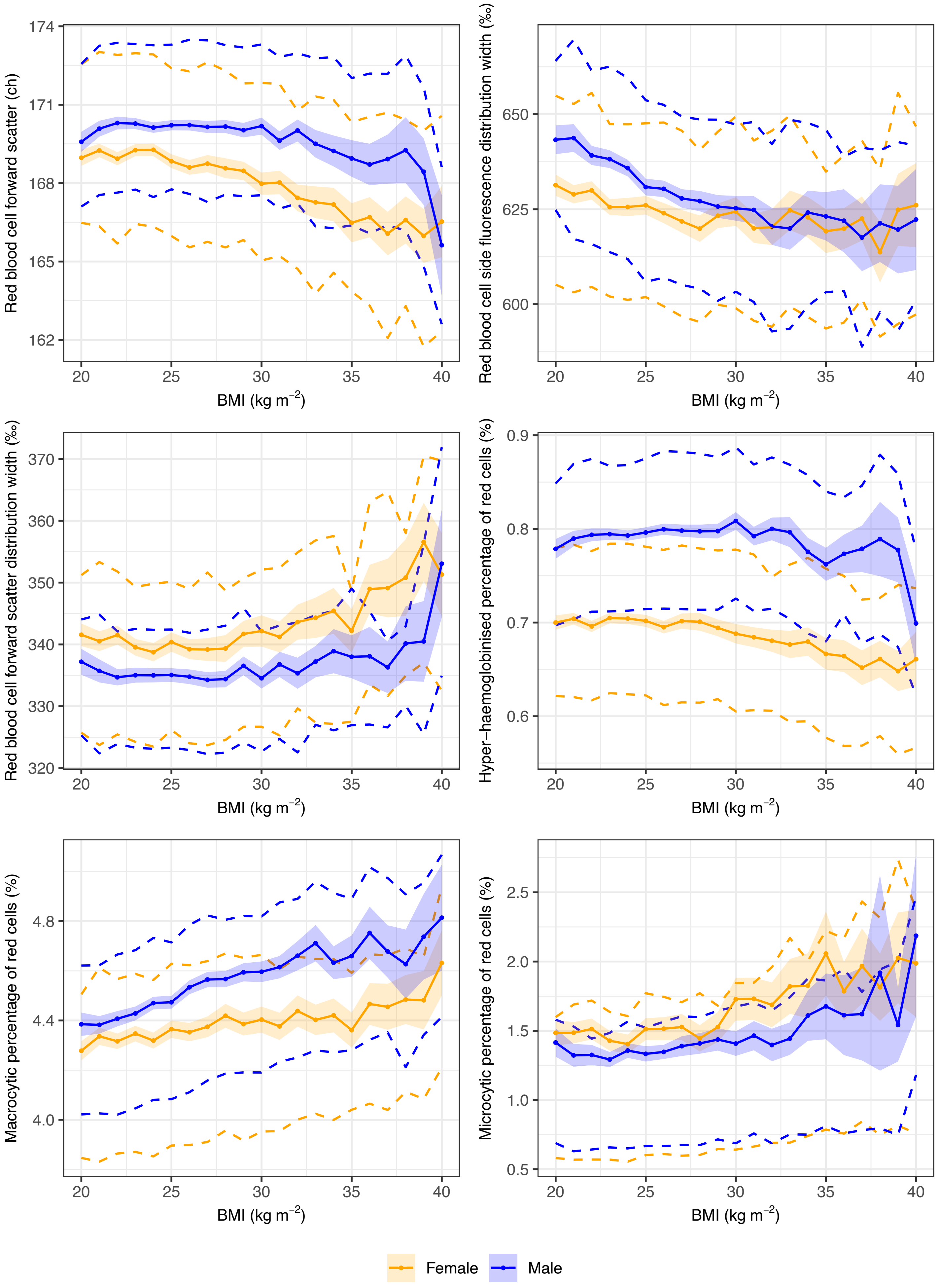

Red blood cell forward scatter (ch)
Red blood cell side fluorescence distribution width (‰)
Red blood cell forward scatter distribution width (‰)
Hyper−haemoglobinised percentage of red cells (%)
Macrocytic percentage of red cells (%)
Microcytic percentage of red cells (%)
BMI (kg m$^{-2}$)
Female
Male

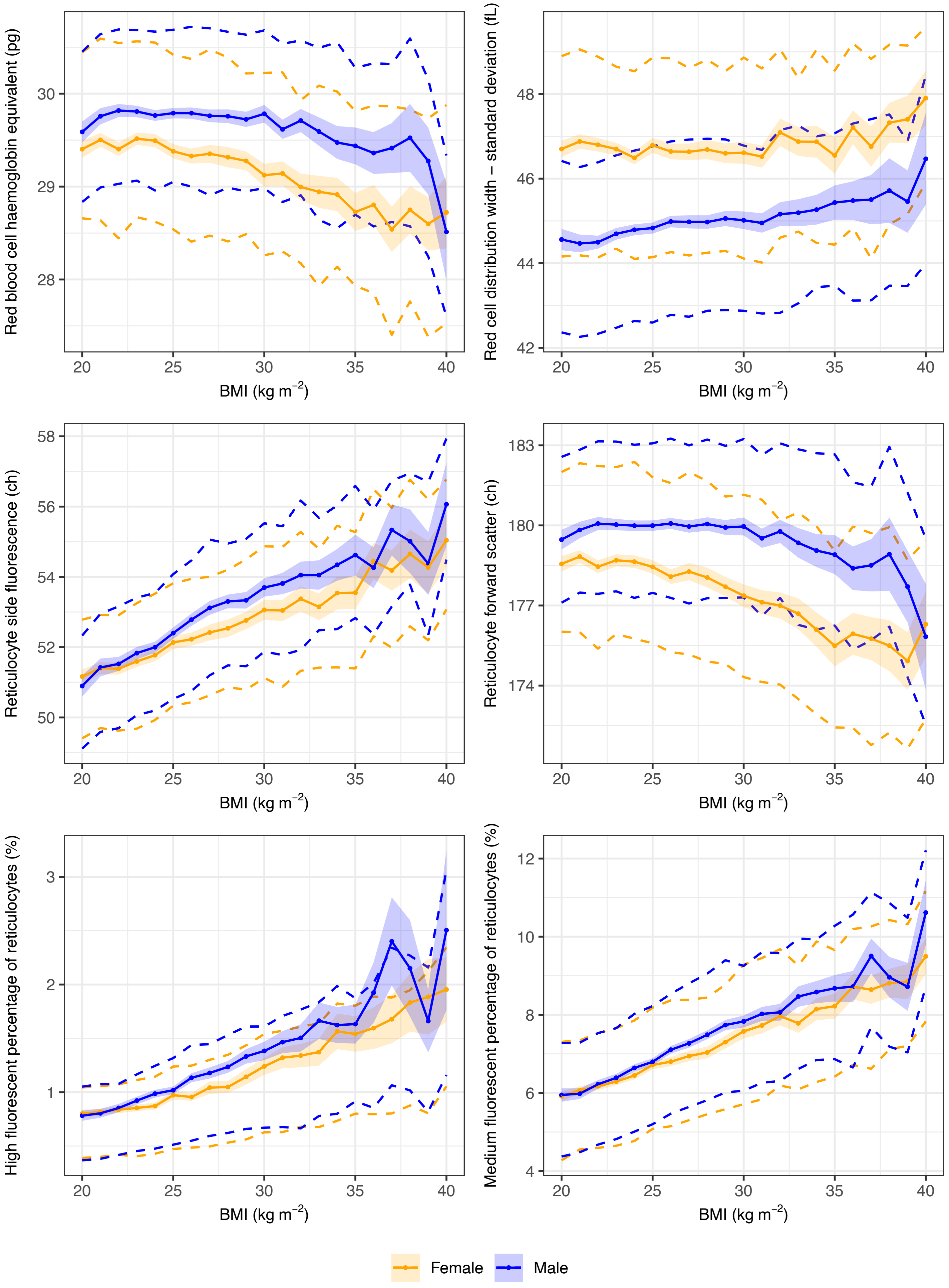
Red blood cell haemoglobin equivalent (pg)
Red cell distribution width – standard deviation (fL)
Reticulocyte side fluorescence (ch)
Reticulocyte forward scatter (ch)
High fluorescent percentage of reticulocytes (%)
Medium fluorescent percentage of reticulocytes (%)
BMI (kg m$^{-2}$)
Female
Male

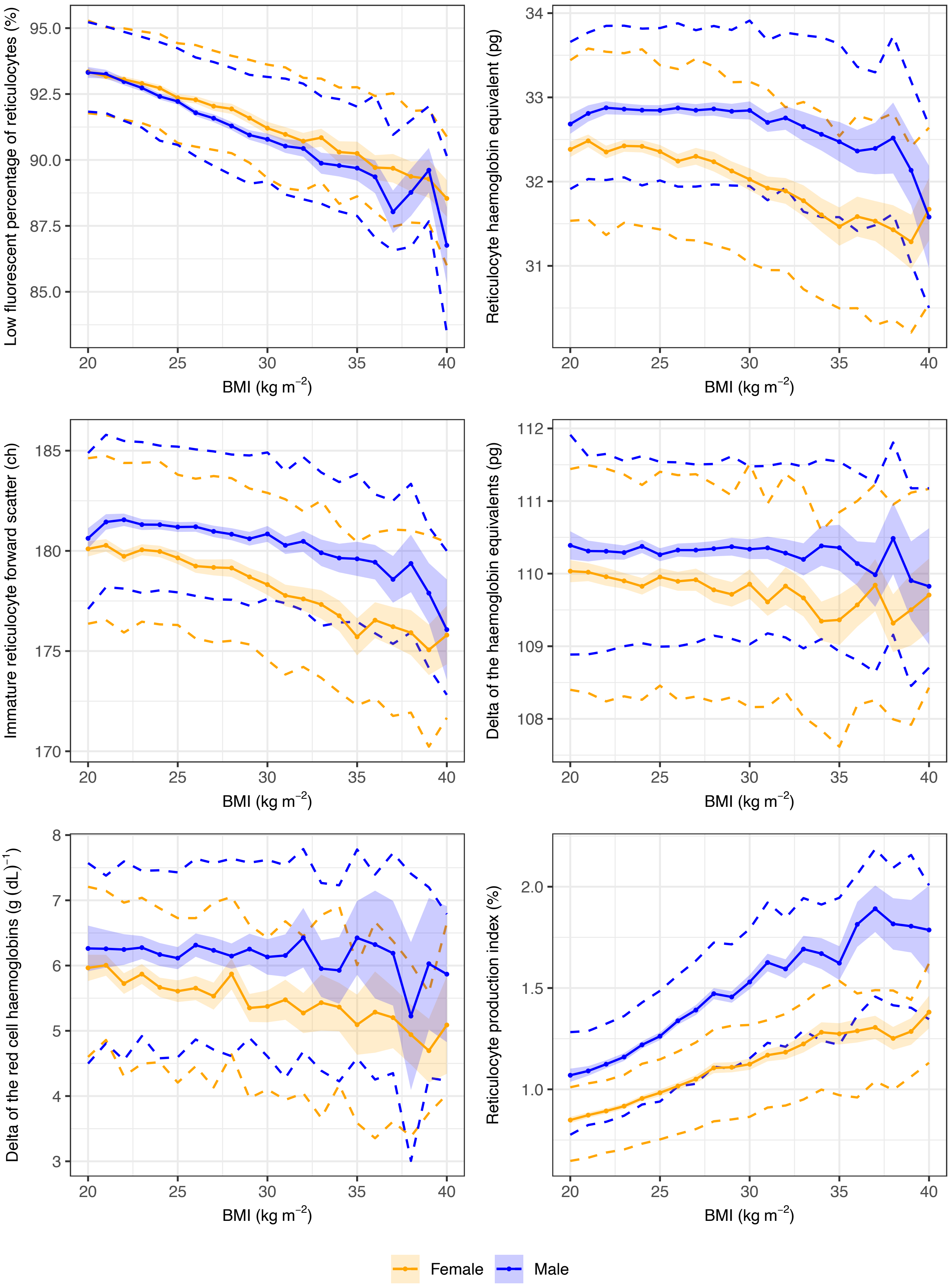
Low fluorescent percentage of reticulocytes (%)
Reticulocyte haemoglobin equivalent (pg)
Immature reticulocyte forward scatter (ch)
Delta of the haemoglobin equivalents (pg)
Delta of the red cell haemoglobins (g (dL)^-1)
Reticulocyte production index (%)
BMI (kg m^-2)
Female
Male

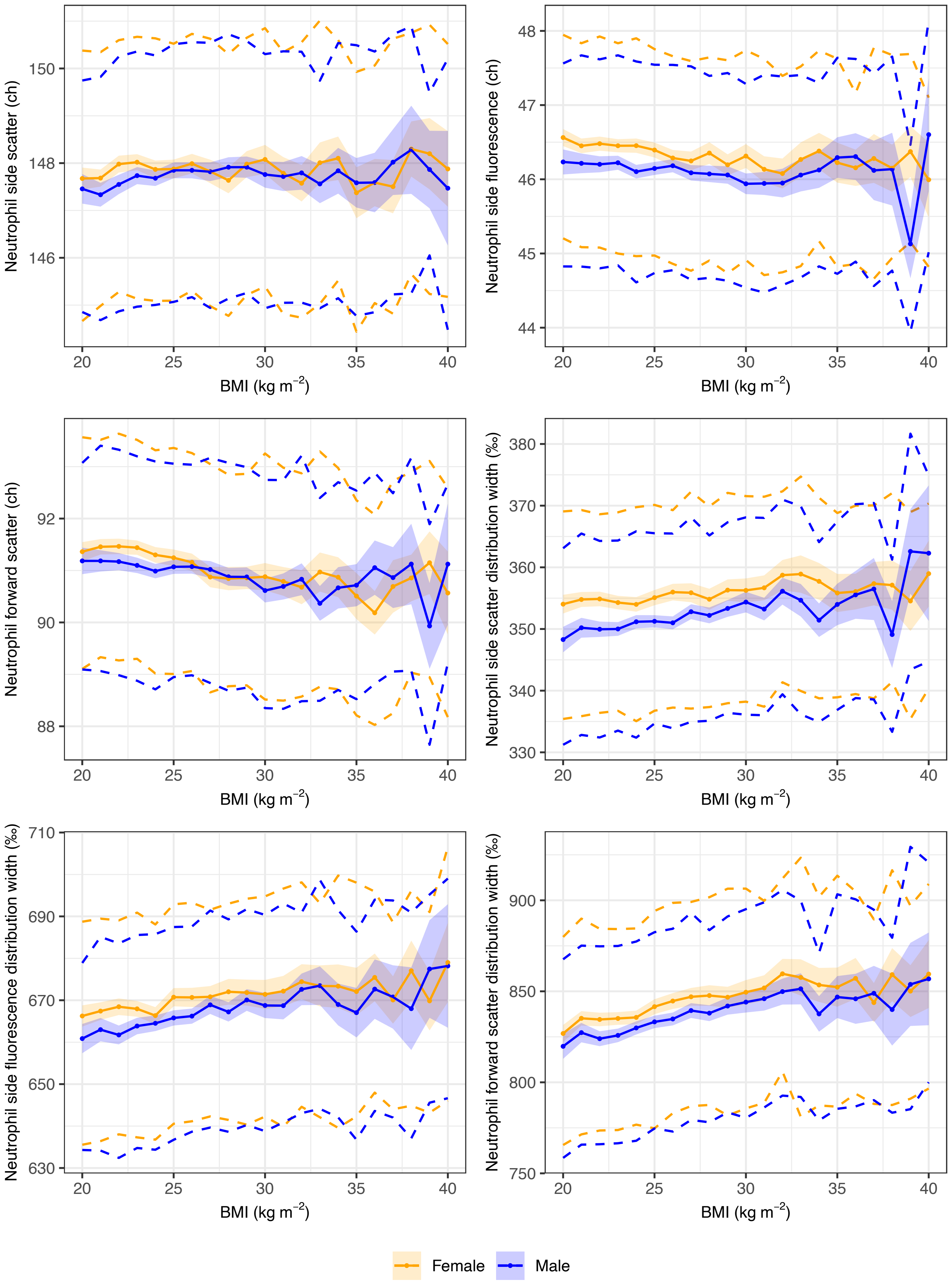

Neutrophil side scatter (ch)
Neutrophil side fluorescence (ch)
Neutrophil forward scatter (ch)
Neutrophil side scatter distribution width (‰)
Neutrophil side fluorescence distribution width (‰)
Neutrophil forward scatter distribution width (‰)
BMI (kg m$^{-2}$)
Female
Male

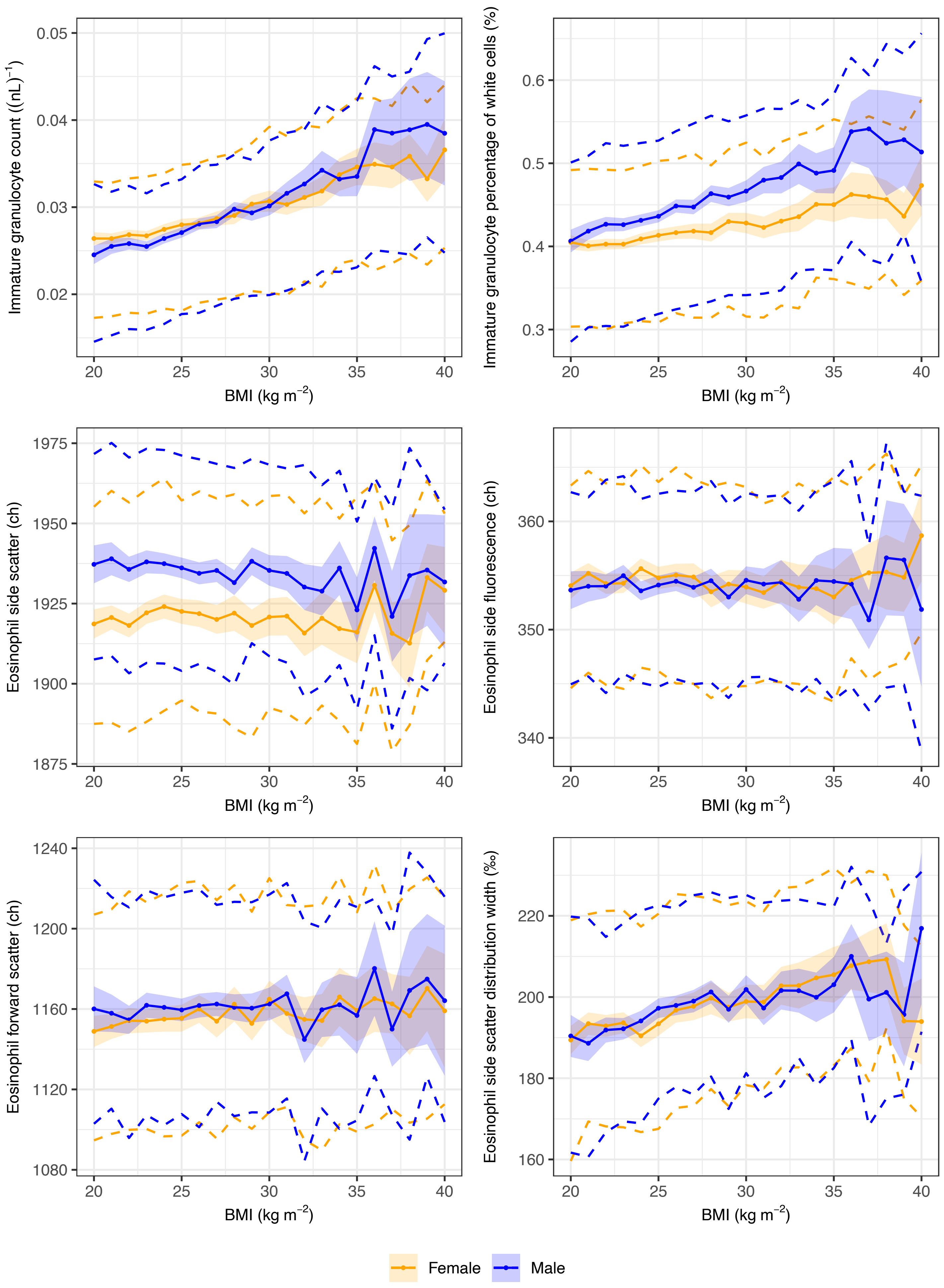
Immature granulocyte count ((nL)$^{-1}$)
Immature granulocyte percentage of white cells (%)
Eosinophil side scatter (ch)
Eosinophil side fluorescence (ch)
Eosinophil forward scatter (ch)
Eosinophil side scatter distribution width (‰)
BMI (kg m$^{-2}$)
Female
Male

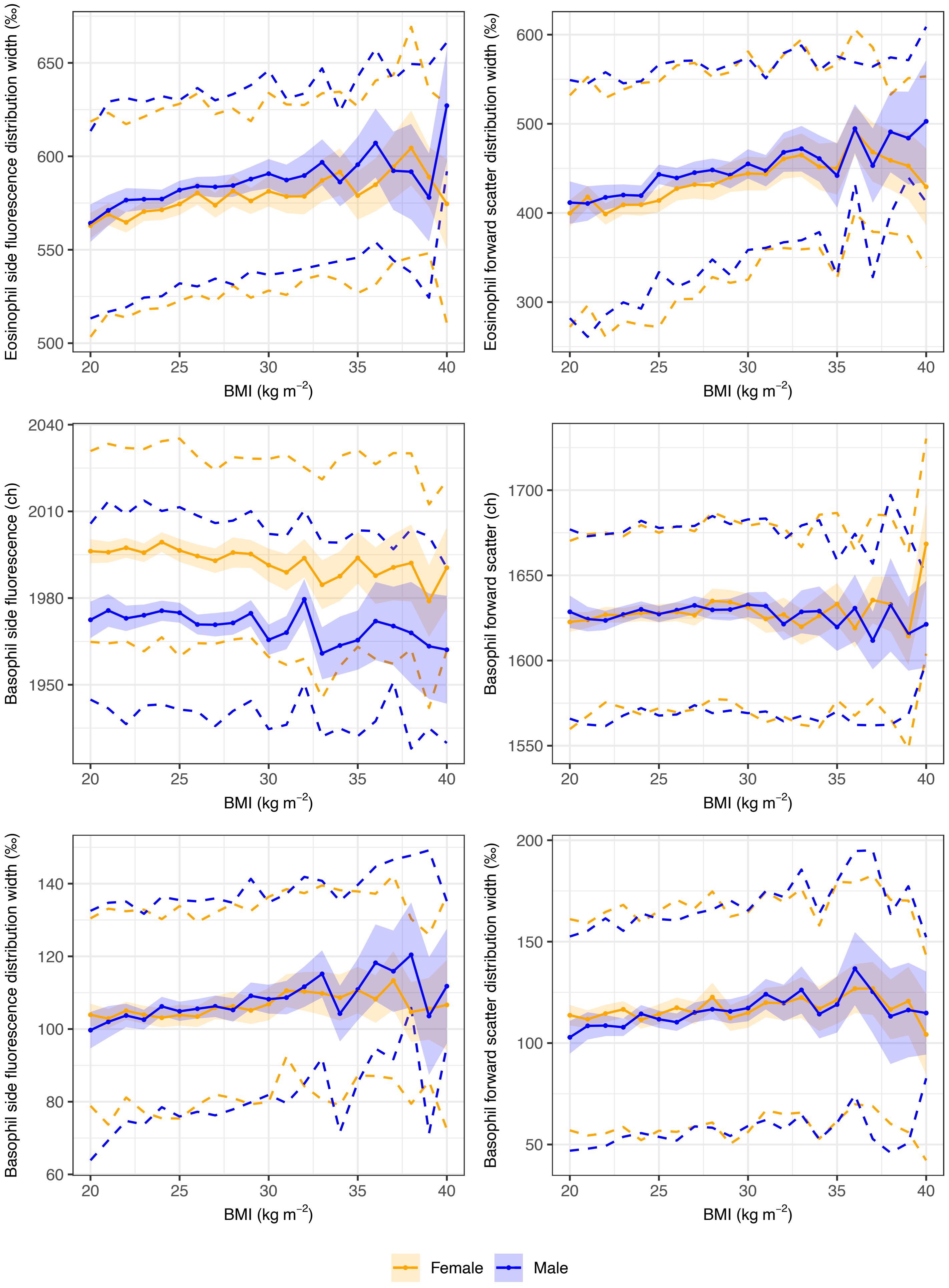
Eosinophil side fluorescence distribution width (‰)
Eosinophil forward scatter distribution width (‰)
Basophil side fluorescence (ch)
Basophil forward scatter (ch)
Basophil side fluorescence distribution width (‰)
Basophil forward scatter distribution width (‰)
BMI (kg m$^{-2}$)
Female
Male

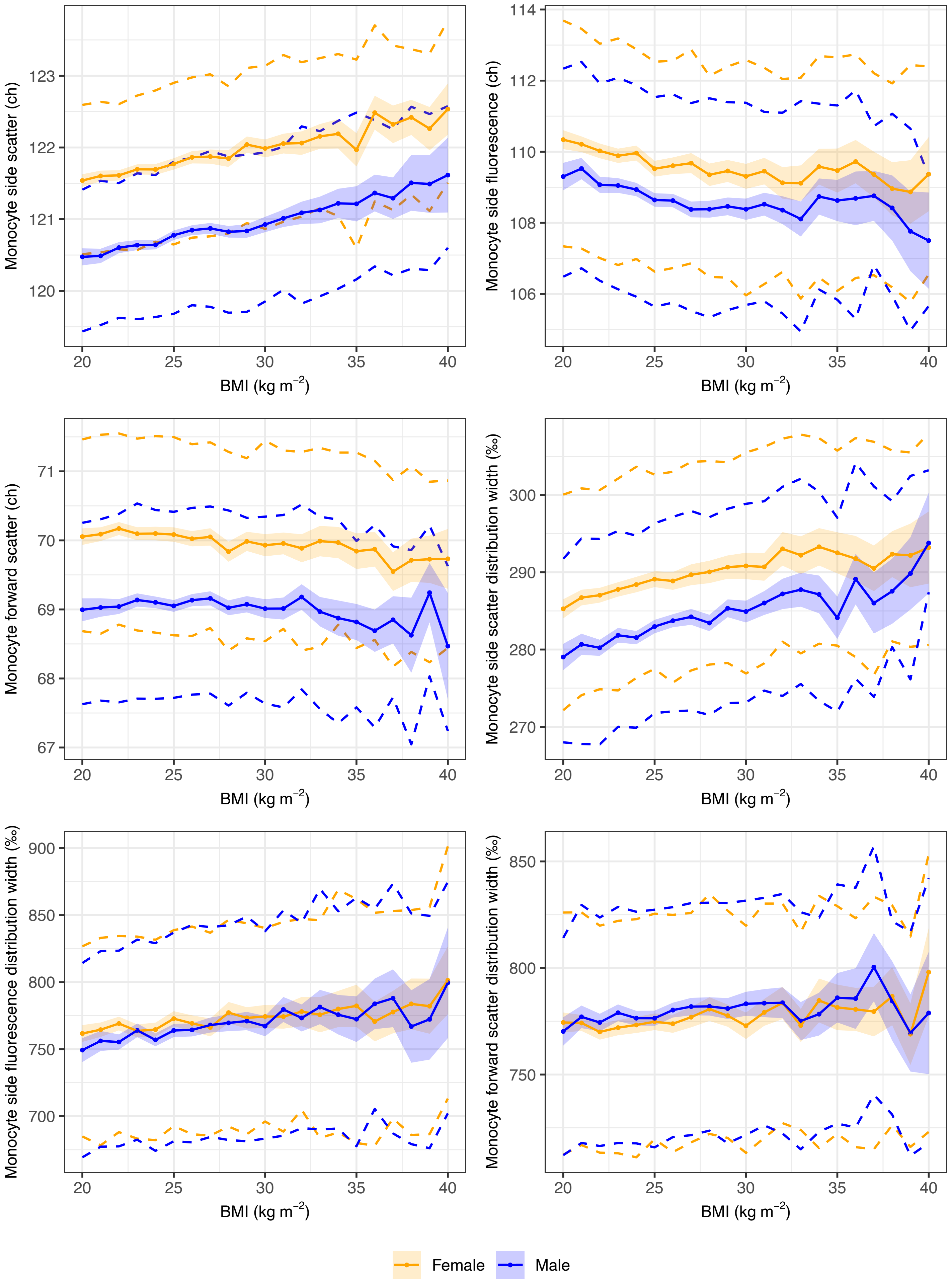
Monocyte side scatter (ch)
Monocyte side fluorescence (ch)
Monocyte forward scatter (ch)
Monocyte side scatter distribution width (‰)
Monocyte side fluorescence distribution width (‰)
Monocyte forward scatter distribution width (‰)
BMI (kg m⁻²)
Female
Male

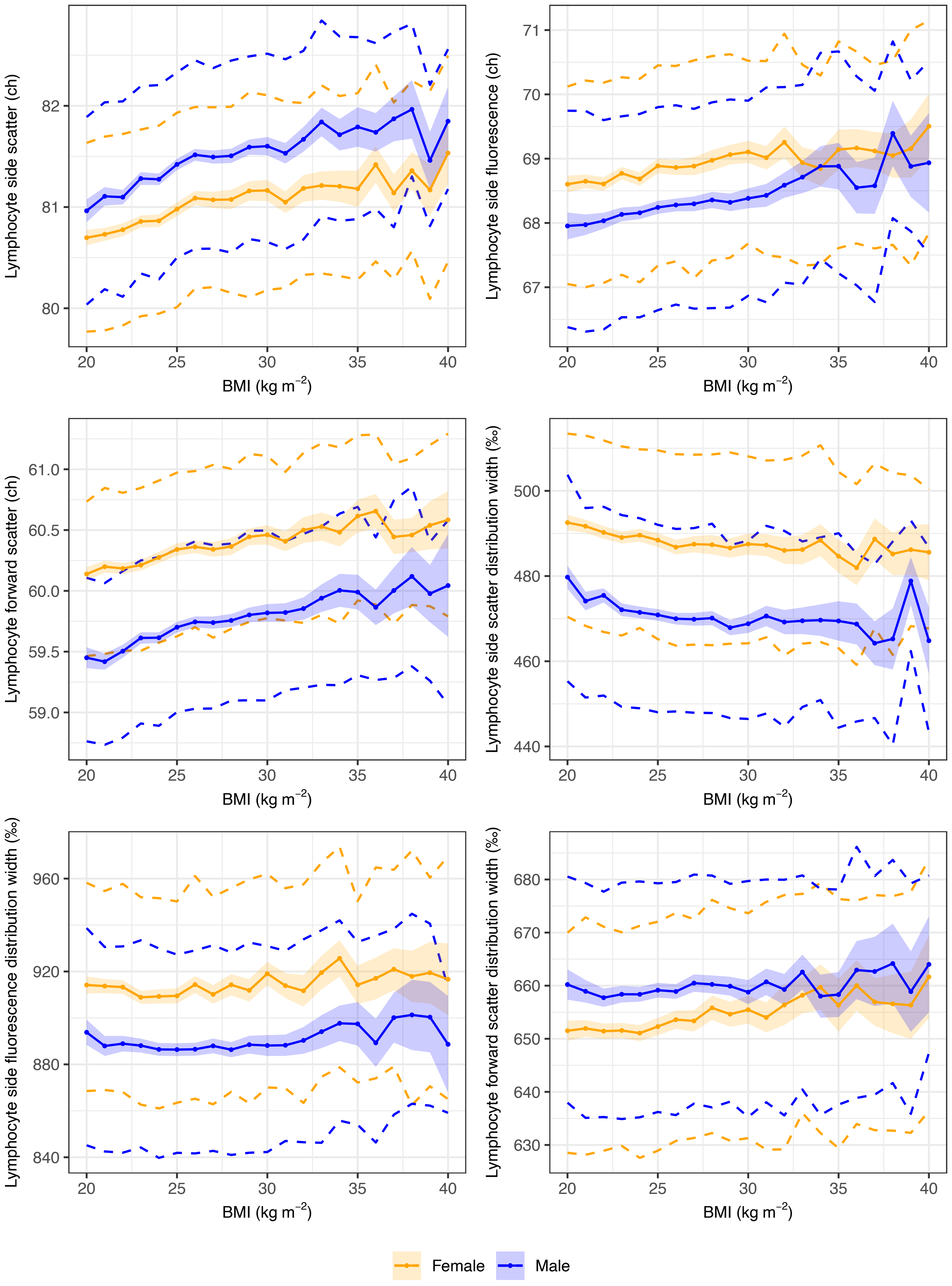
Lymphocyte side scatter (ch)
80
81
82
BMI (kg m$^{-2}$)
Lymphocyte side fluorescence (ch)
67
68
69
70
71
Lymphocyte forward scatter (ch)
59.0
59.5
60.0
60.5
61.0
Lymphocyte side scatter distribution width (‰)
440
460
480
500
Lymphocyte side fluorescence distribution width (‰)
840
880
920
960
Lymphocyte forward scatter distribution width (‰)
630
640
650
660
670
680
20
25
30
35
40
Female
Male

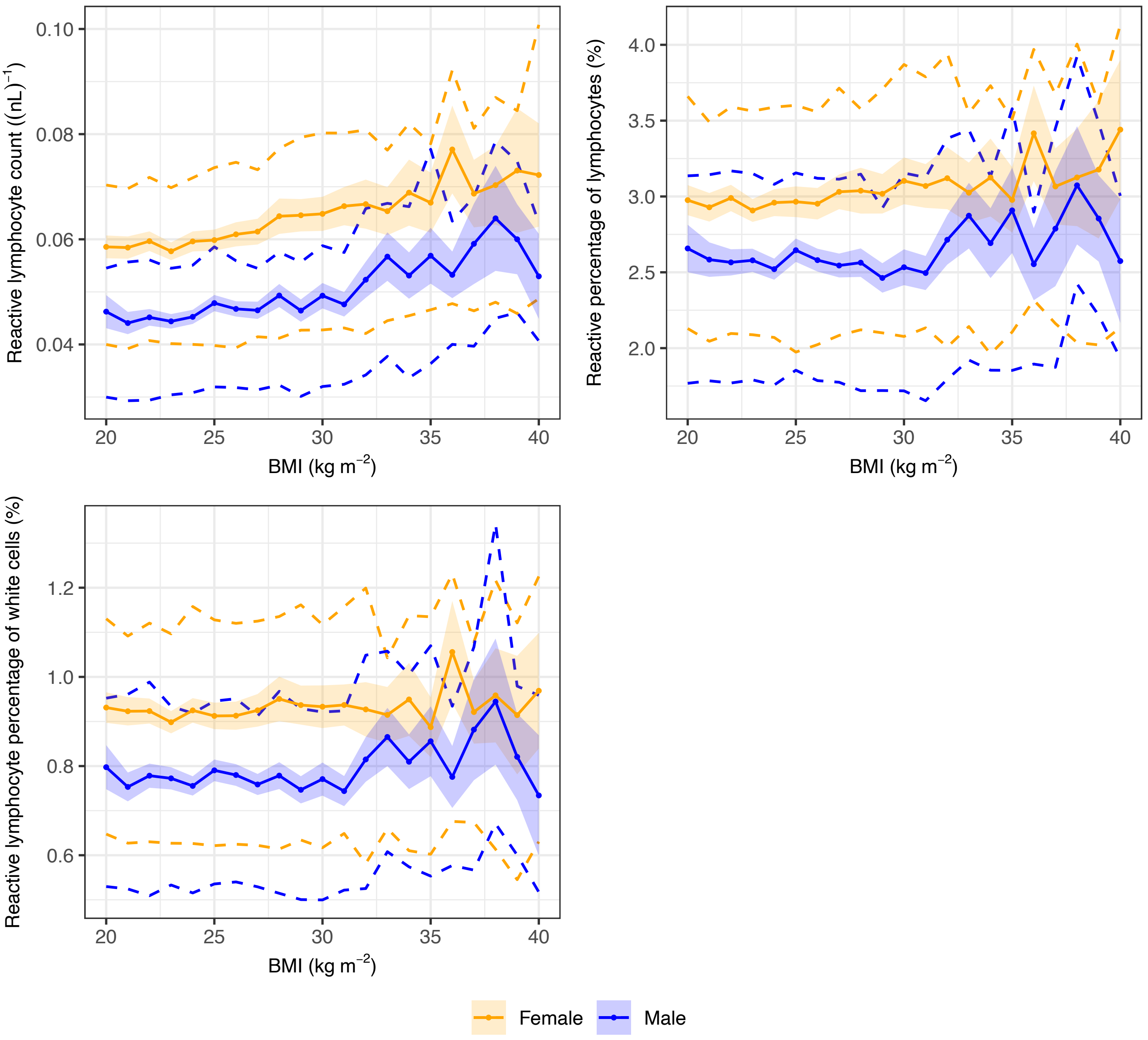


**Supplementary Fig. 3 | Covariation between technically adjusted ncCBC traits and BMI stratified by sex.** Covariation between the 63 nCBC traits and participant body mass index (BMI) stratified by participant sex. Parameters of the stratified trait distributions were estimated in bins corresponding to 1kg $m^{-2}$ of BMI. The linearly interpolated coloured points show estimates of the within strata-means and the underlying coloured ribbons show the corresponding 95% confidence intervals. The dashed lines show estimates of the upper and lower quartiles. The data are from the technically adjusted traits (Methods) restricted to the participants who contribute data to the GWAS of the respective trait, the numbers of which are given in Supplementary Data 2.

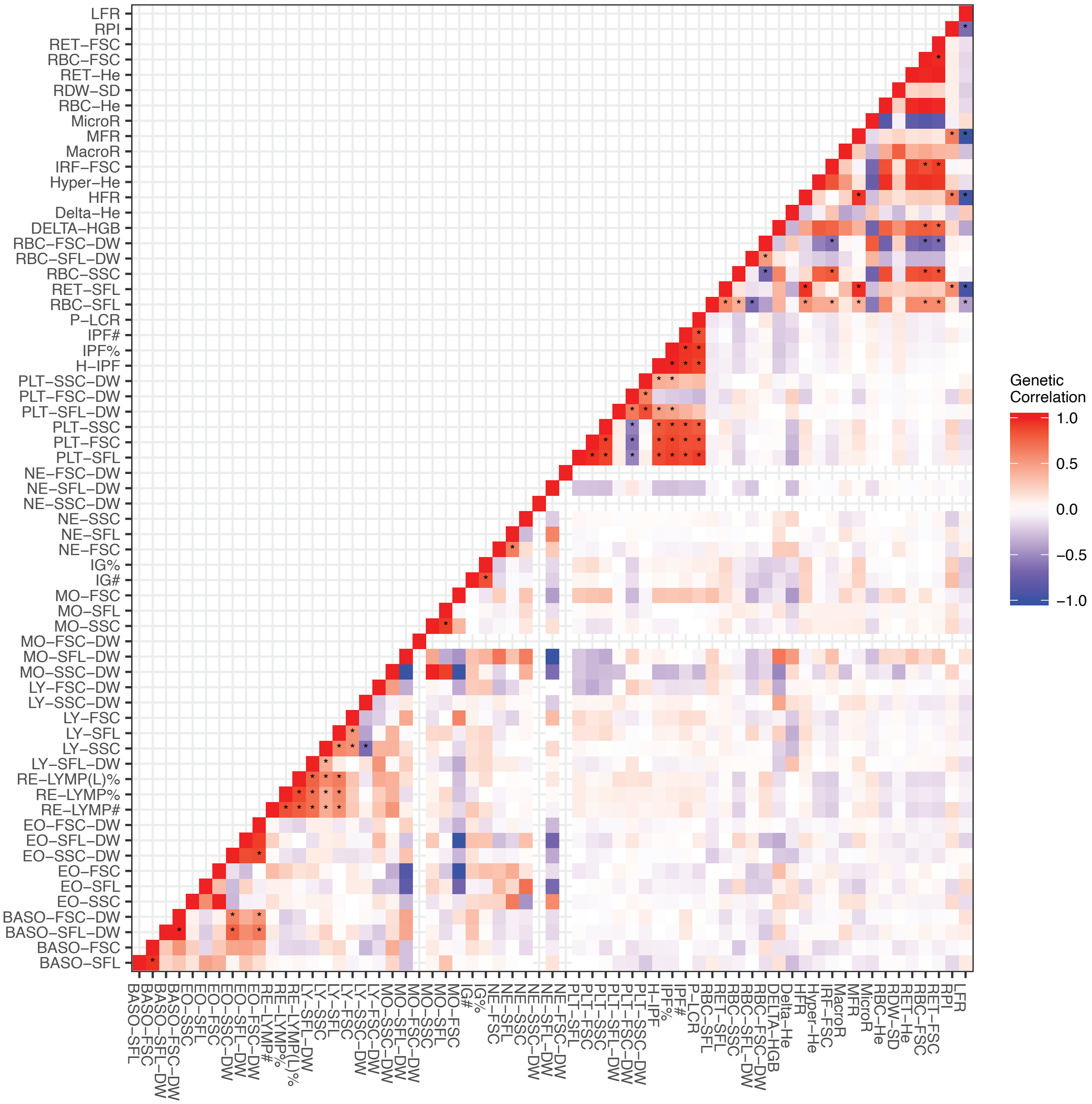


**Supplementary Fig. 4 | Genetic correlation within ncCBC traits.** A heatmap of estimates of genetic correlation between ncCBC phenotypes, made by applying LD Score regression to GWAS summary statistics[1,2]. The transparent rows and columns correspond to phenotypes (NE-FSC-DW, NE-SSC-DW, MO-FSC-DW) for which it was not possible to estimate genetic correlation due to an insufficiency of genetic association signals. The absolute genetic correlation between pairs of phenotypes of platelets and between pairs of phenotypes of red cells is generally higher than the absolute genetic correlation between pairs of phenotypes of subtypes of white cells. There is limited evidence of strong absolute correlations between pairs of phenotypes of different cell-types. The starred cells are those for which a (two-sided) test of the null-hypothesis that the corresponding correlation is equal to zero is rejected at a Bonferroni corrected significance threshold ($P$-value < $1.28 \times 10^{-5}$).

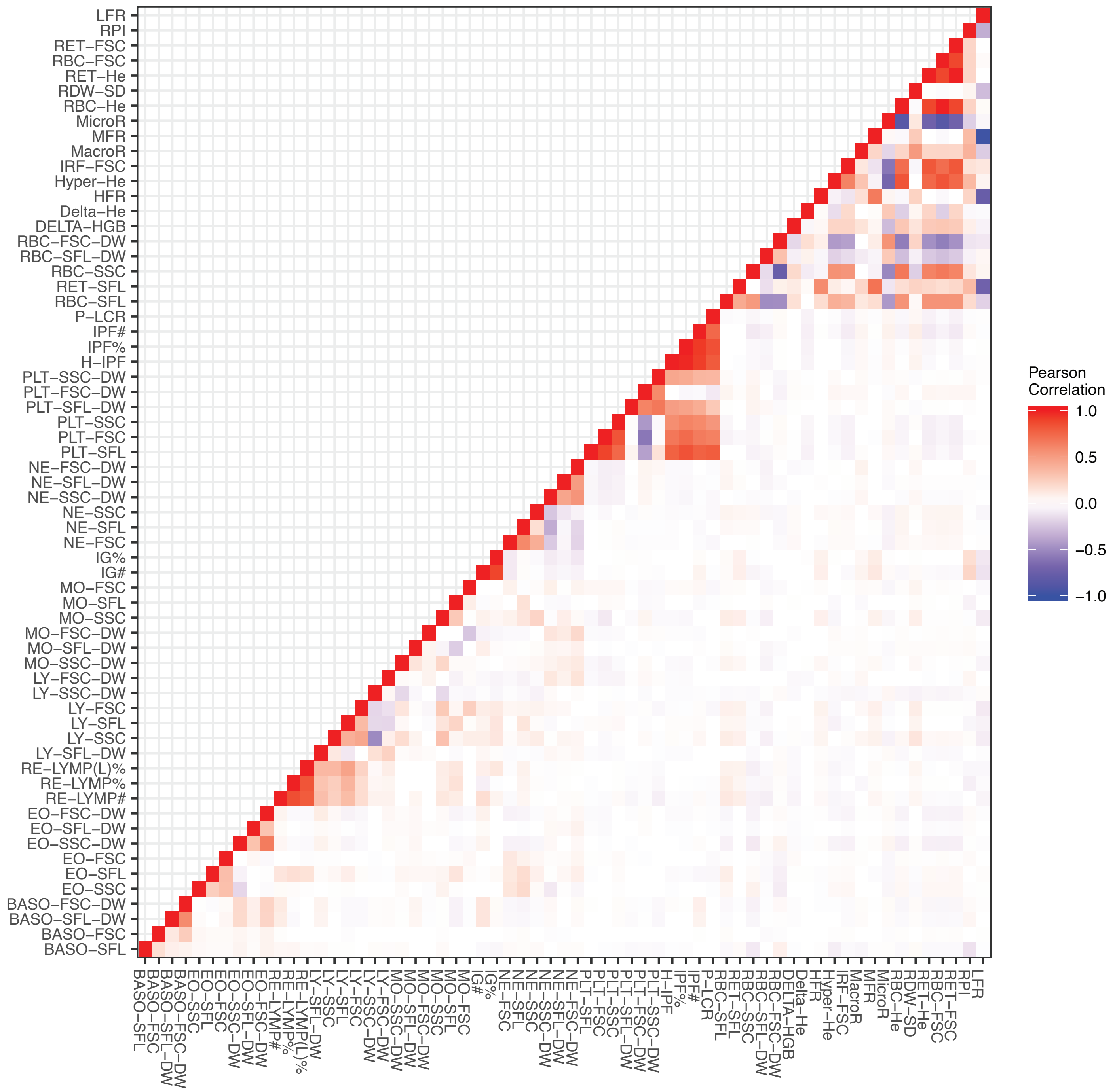


**Supplementary Fig. 5 | Phenotypic correlation within ncCBC traits.** A heatmap of the Pearson correlations between the ncCBC phenotypes in the sub-sample of the INTERVAL cohort used for the GWAS analysis. Correlations between pairs of phenotypes of red cells and pairs of phenotypes of platelets are stronger on average than correlations between pairs of phenotypes of white cells. There is low correlation between pairs of phenotypes of different cell-types.

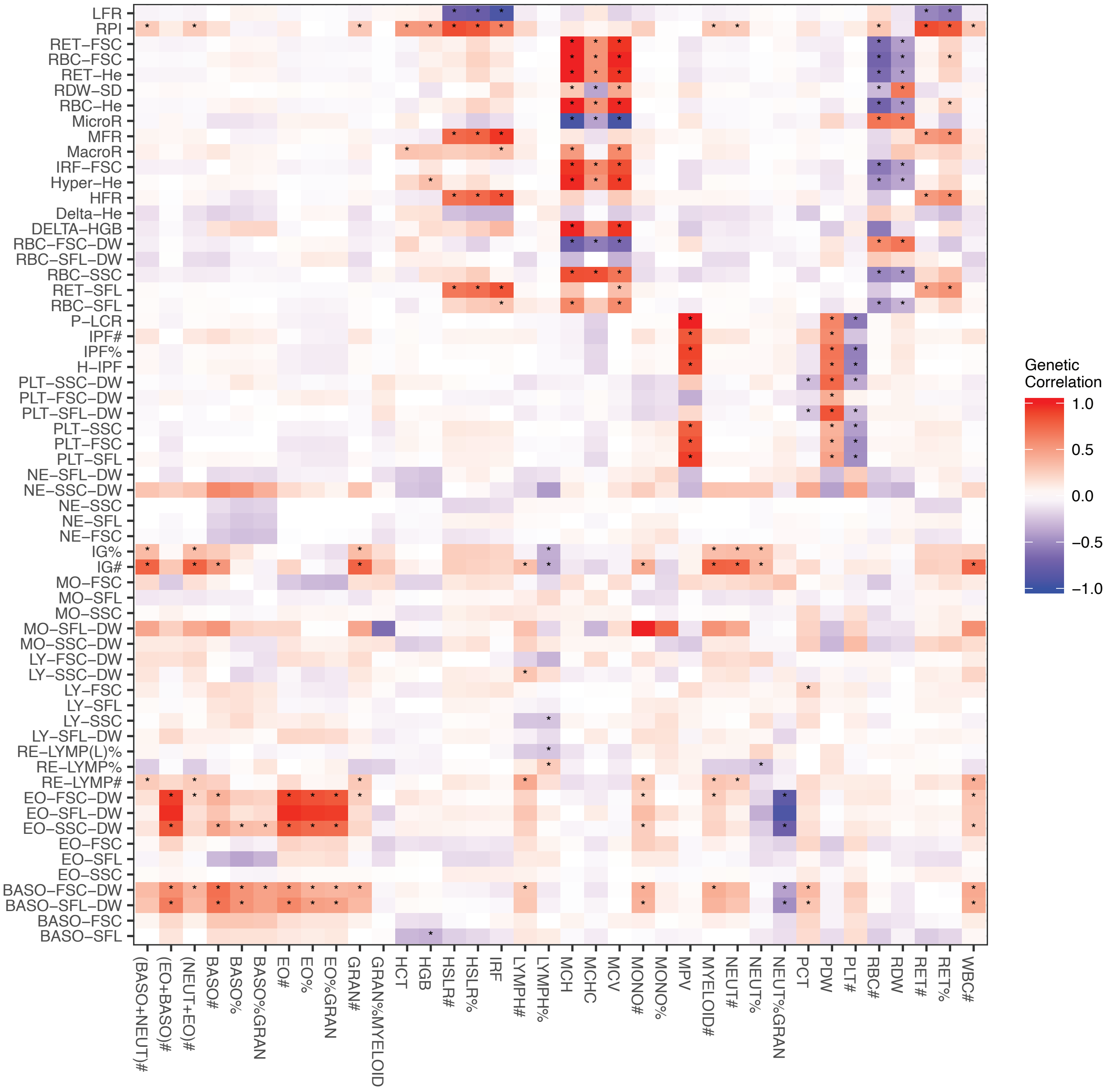


**Supplementary Fig. 6 | Genetic correlation between ncCBC traits and cCBC traits.** A heatmap of estimates of genetic correlation between the ncCBC traits and the haematological traits studied by Vuckovic et al.[3] made by applying LD Score regression to GWAS summary statistics generated from the INTERVAL cohort[4]. There is limited genetic correlation between ncCBC phenotypes of white cells and previously studied standard haematological traits. The starred cells are those for which a (two-sided) test of the null-hypothesis that the corresponding correlation is equal to zero is rejected at a Bonferroni corrected significance threshold (*P*-value $< 2.27 \times 10^{-5}$).

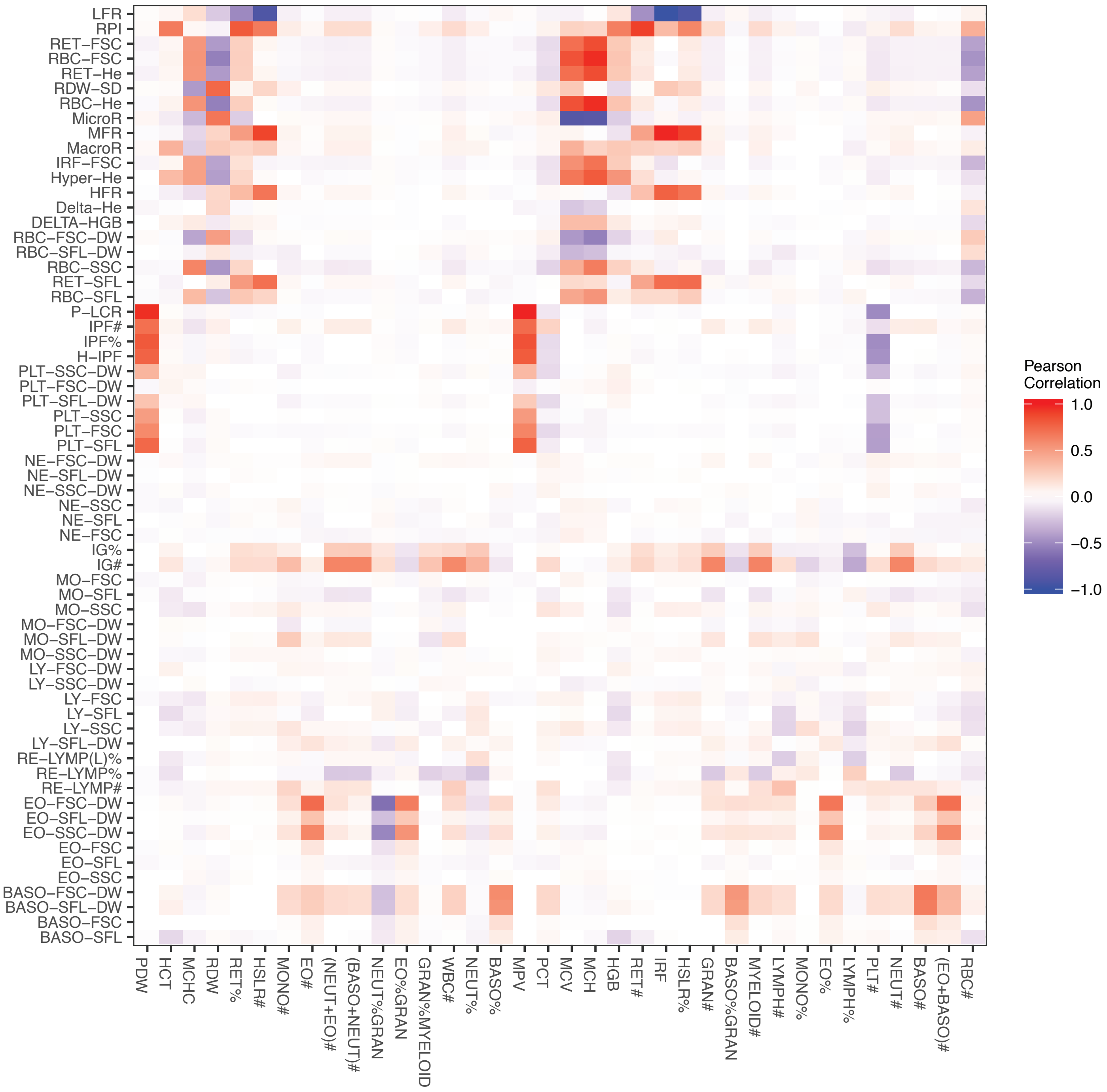


**Supplementary Fig. 7 | Phenotypic correlation between ncCBC traits and cCBC traits.** A heatmap of the Pearson correlation between the ncCBC phenotypes and the haematological traits studied by Vuckovic et al.[3] in the sub-sample of the INTERVAL cohort used for the GWAS analysis[4]. There is limited Pearson correlation between ncCBC phenotypes of white cells and previously studied standard haematological traits.

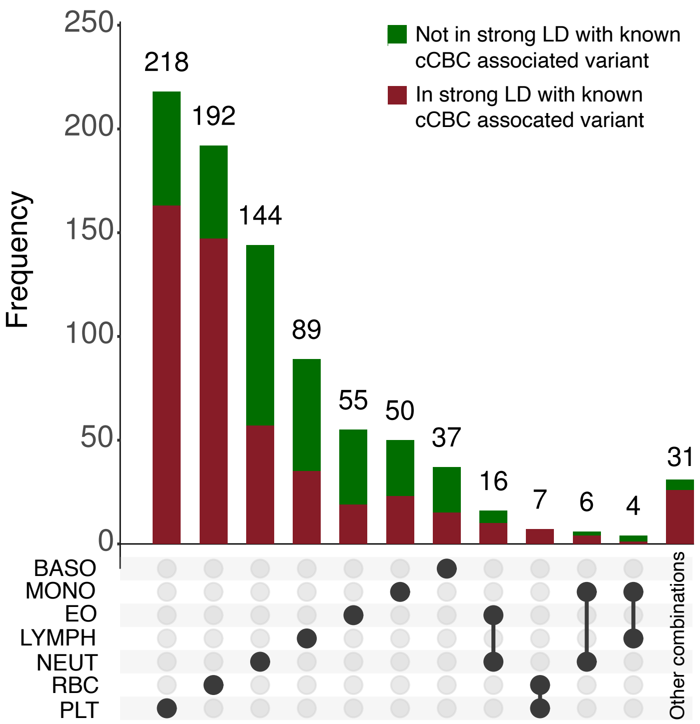


**Supplementary Fig. 8 | The joint distribution of the number of LD clumps by cell-type.** A bar plot counting LD clumps by the cell-types of the ncCBC traits with which the variants they contain are associated. For all but 64 of the clumps, the variants in each clump were associated with traits of a single cell-type. The green fraction of each bar corresponds to the LD clumps that contain no variant in LD ($r^2$>0.8) with a variant identified by Vuckovic et al. or Chen et al.[3,5]

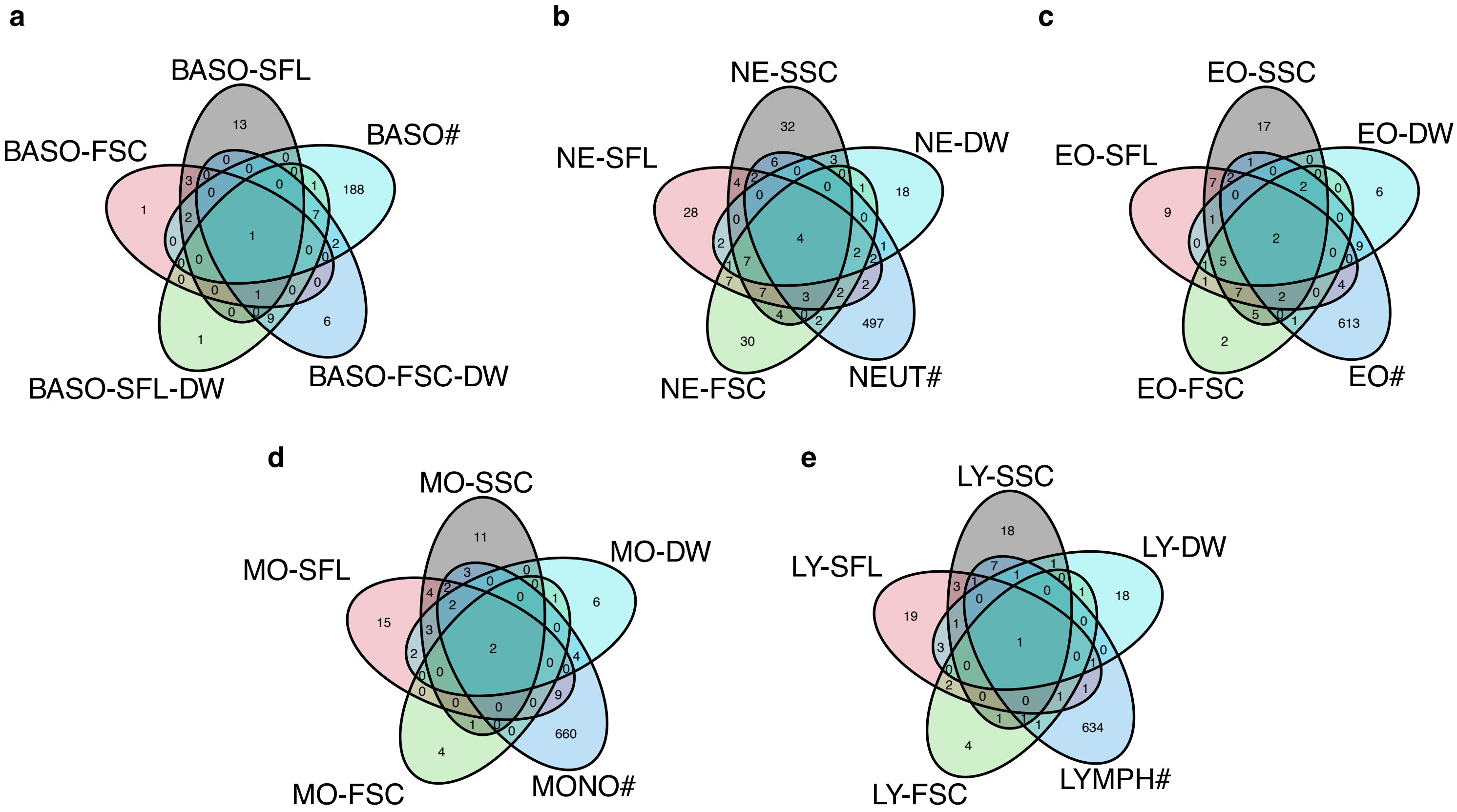


**Supplementary Fig. 9 | The joint distribution of the number of LD clumps by traits within cell-types.** Venn diagrams showing the count distribution of LD clumps cross-classified by association with selected ncCBC (-SSC, -SFL, -FSC, -SSC-DW, -SFL-DW, -FSC-DW) traits and CBC cell count traits of basophils (**a**), neutrophils (**b**), eosinophils (**c**), monocytes (**d**) and lymphocytes (**e**). The suffix '-DW' absent a trait type (i.e. absent -SSC, -SFL, -FSC) means any of the distribution width traits of the given cell-type. (e.g. the region labelled NE-DW counts clumps containing at least one variant associated with one of NE-SSC-DW, NE-SFL-DW or NE-FSC-DW).

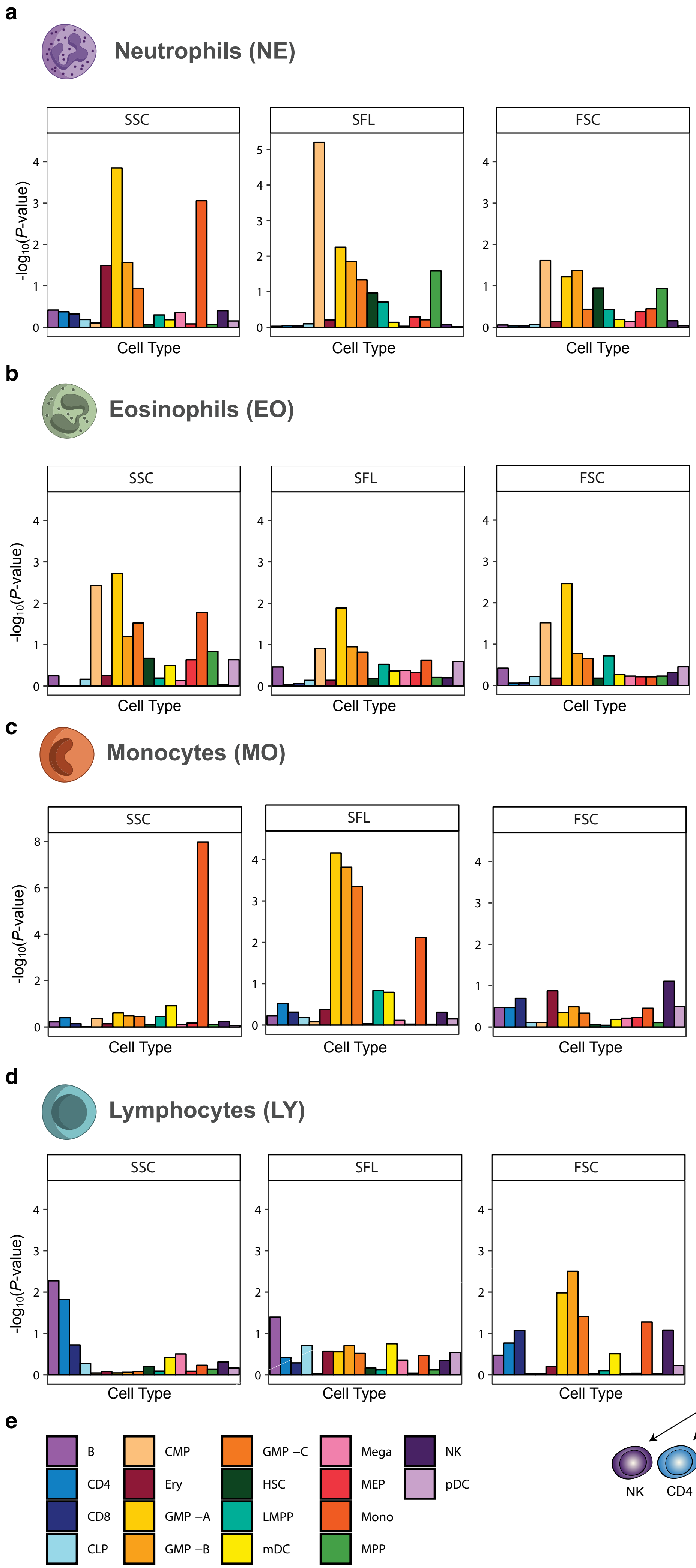


**Supplementary Fig. 10 | ATAC-seq enrichment analysis for neutrophils (a), eosinophils (b), monocytes (c), and lymphocytes (d).** Bar plots showing -$\log_{10}$(*P*-value)s for tests (performed by g-chromVAR) of the null hypothesis that there is no enrichment of associated genetic-variants in regions of open chromatin adjusting for GC content and average ATAC-seq peak intensity. Each sub-panel corresponds to associations with a different flow cytometry phenotype. Each bar corresponds to regions of open chromatin measured in a different cell-type. No adjustments have been made for multiple testing. **e** A legend for panels a-d indicating the cell-type corresponding to the colour of each bar. **f** Diagram of a haematopoietic tree with cell-types coloured according to the scheme displayed in panel e. We thank Joanna Westmoreland for the artwork in panels a-d; panel f is reproduced with permission from Ulirsch, J. C. et al. Interrogation of human hematopoiesis at single-cell and single-variant resolution. Nat. Genet. 51, 683–693 (2019), Springer Nature[6].

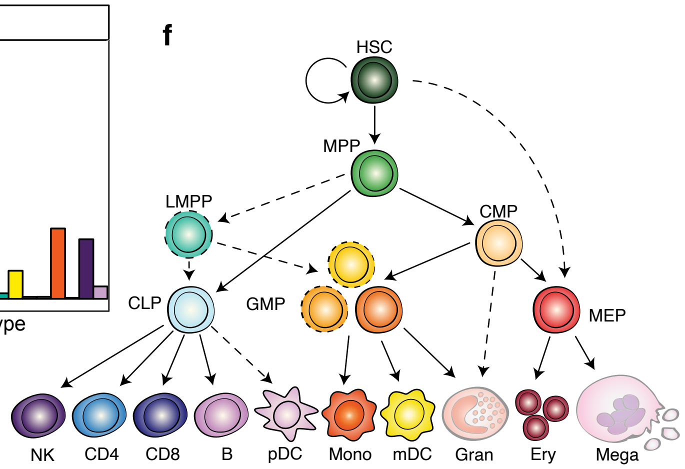

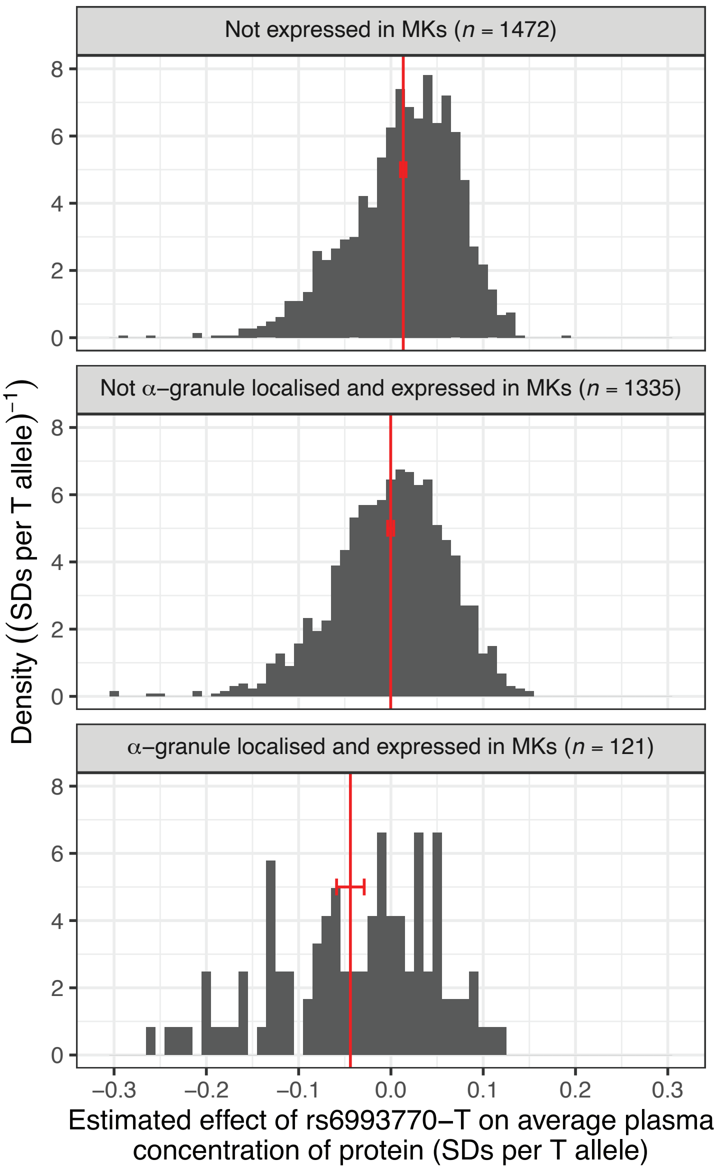


**Supplementary Fig. 11 | Distribution of effect sizes of genetic associations between rs6993770-T and plasma protein concentrations by protein location.** Histograms of estimates of the additive allelic effect size (measured in SD of phenotypic variance per allele) of rs69933770-T on the mean concentration of 2,928 plasma proteins, taken from Sun et al.[7] The mean effect size amongst proteins expressed in MKs (bottom and middle panel) is lower than the mean effect size amongst proteins which are not expressed in MKs (top panel). Within MKs, the mean effect size amongst proteins localised to α-granules (bottom panel) is lower than the mean effect size amongst proteins expressed in MKs but not localised to α-granules (middle panel). The difference in averages, adjusted for gene expression level measured by RNA-seq of MKs, was estimated to be 0.038 phenotypic standard deviations per allele (*P*-value $5.6 \times 10^{-10}$), implying that rs6993770-T differentially reduces the plasma concentration of proteins expressed in MKs according to whether or not they are stored in α-granules. The vertical red lines indicate the means of the corresponding histograms. The red error bars represent the 95% confidence intervals of the estimates of the means.

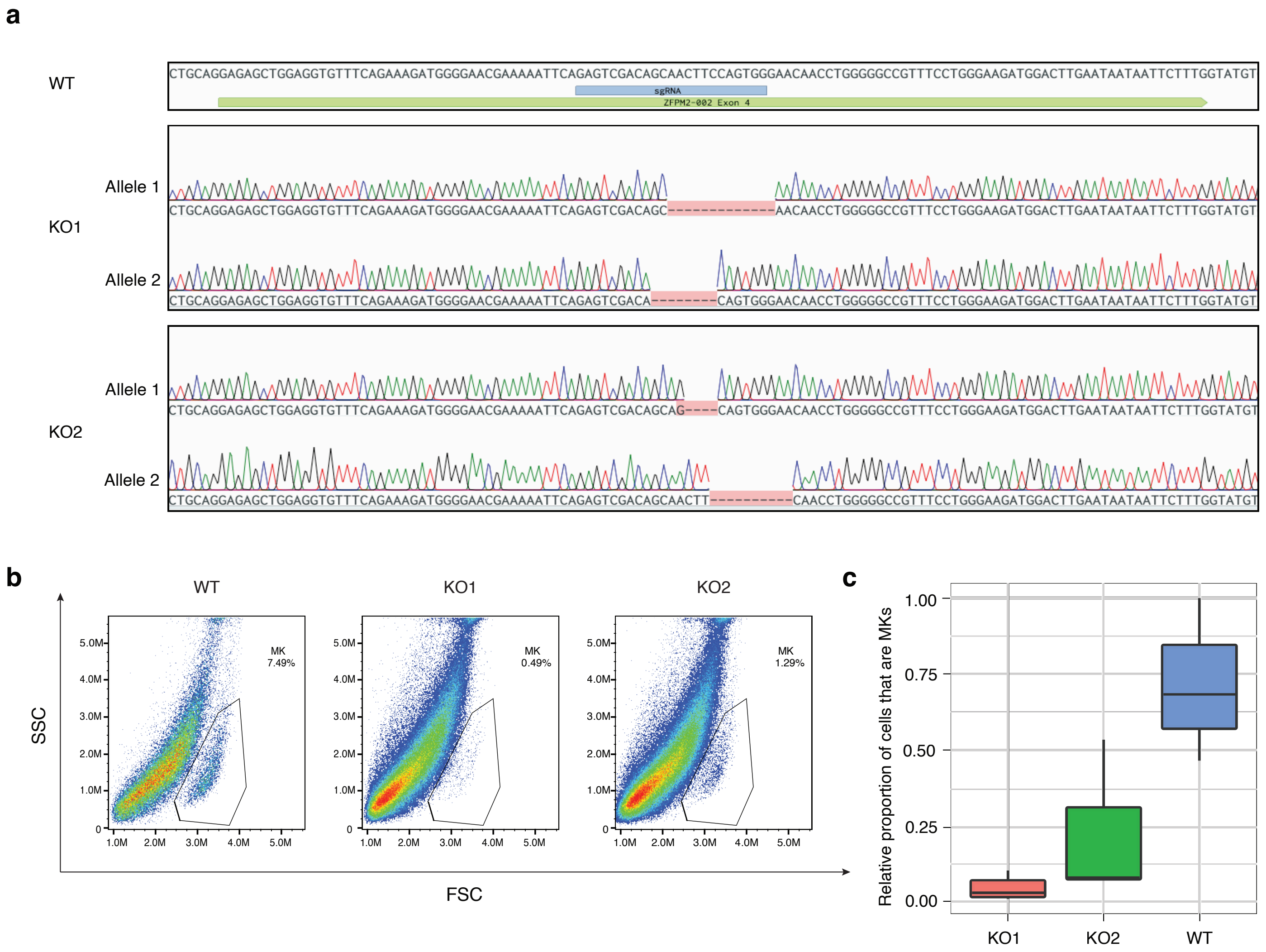


**Supplementary Fig. 12 | Effect of ablation of *ZFPM2* on megakaryopoiesis. a** Confirmation by Sanger sequencing of the genotypes of, and the out-of-frame deletions in, the *ZFPM2* knockout cell lines (KO1 and KO2). Red shadings highlight mutations and deletions with respect to the reference sequence, which is that of the wild type (WT) cell line. **b** Flow cytometry plots from arbitrarily selected technical replicates of the differentiated WT, KO1 and KO2 cell lines (FSC = forward scatter; SSC = side scatter). The gated regions correspond to the MK populations. The overlying numbers indicate the percentage of cells in each plot that are gated. **c** Box plot showing, for each biological replicate, the distribution of the proportion of cells in each nested technical replicate (three per cell line) that are MKs relative to the greatest such proportion amongst the wild type technical replicates. The centre line and the lower and upper hinges of each box plot correspond respectively to the median and the first and third quartiles of the data. The upper whiskers extend to the greatest data point no greater than the third quartile plus 1.5 times the interquartile range. The lower whiskers are defined symmetrically.

## Supplementary References


1. Bulik-Sullivan, B. K. et al. LD Score regression distinguishes confounding from polygenicity in genome-wide association studies. *Nat. Genet.* **47**, 291–295 (2015).
2. Bulik-Sullivan, B. et al. An atlas of genetic correlations across human diseases and traits. *Nat. Genet.* **47**,1236–1241 (2015).
3. Vuckovic, D. et al. The Polygenic and Monogenic Basis of Blood Traits and Diseases. *Cell* **182**, 1214–1231.e11 (2020).
4. Astle, W. J. et al. The Allelic Landscape of Human Blood Cell Trait Variation and Links to Common Complex Disease. *Cell* **167**, 1415–1429.e19 (2016).
5. Chen, M.-H. et al. Trans-ethnic and Ancestry-Specific Blood-Cell Genetics in 746,667 Individuals from 5 Global Populations. *Cell* **182**, 1198–1213.e14 (2020).
6. Ulirsch, J. C. et al. Interrogation of human hematopoiesis at single-cell and single-variant resolution. *Nat. Genet.* **51**, 683–693 (2019).
7. Sun, B. B. et al. Genomic atlas of the human plasma proteome. *Nature* **558**, 73–79 (2018).
8. Ferreira, M. A. et al. Shared genetic origin of asthma, hay fever and eczema elucidates allergic disease biology. *Nat. Genet.* **49**, 1752–1757 (2017).
9. Lambert, J. et al. Meta-analysis of 74,046 individuals identifies 11 new susceptibility loci for Alzheimer's disease. *Nat. Genet.* **45**, 1452–1458 (2013).
10. Demenais, F. et al. Multiancestry association study identifies new asthma risk loci that colocalize with immune-cell enhancer marks. *Nat. Genet.* **50**, 42–53 (2018).
11. Dubois, P. C. A. et al. Multiple common variants for celiac disease influencing immune gene expression. *Nat. Genet.* **42**, 295–302 (2010).
12. Trynka, G. et al. Dense genotyping identifies and localizes multiple common and rare variant association signals in celiac disease. *Nat. Genet.* **43**, 1193–1201 (2011).
13. Nikpay, M. et al. A comprehensive 1000 Genomes–based genome-wide association meta-analysis of coronary artery disease. *Nat Genet* **47**, 1121–1130 (2015).
14. Liu, J. Z. et al. Association analyses identify 38 susceptibility loci for inflammatory bowel disease and highlight shared genetic risk across populations. *Nat. Genet.* **47**, 979–986 (2015).
15. Lange, K. M. de et al. Genome-wide association study implicates immune activation of multiple integrin genes in inflammatory bowel disease. *Nat. Genet.* **49**, 256–261 (2017).
16. Paternoster, L. et al. Multi-ancestry genome-wide association study of 21,000 cases and 95,000 controls identifies new risk loci for atopic dermatitis. *Nat. Genet.* **47**, 1449–1456 (2015).
17. Sawcer, S. et al. Genetic risk and a primary role for cell-mediated immune mechanisms in multiple sclerosis. *Nature* **476**, 214–219 (2011).
18. Beecham, A. H. et al. Analysis of immune-related loci identifies 48 new susceptibility variants for multiple sclerosis. *Nat. Genet.* **45**, 1353–1360 (2013).
19. Patsopoulos, N. A. et al. Multiple sclerosis genomic map implicates peripheral immune cells and microglia in susceptibility. *Science* **365**, (2019).
20. Liu, J.Z. et al. Dense fine-mapping study identifies new susceptibility loci for primary biliary cirrhosis. *Nat. Genet.* **44**, 1137–1141 (2012).
21. Cordell, H. J. et al. International genome-wide meta-analysis identifies new primary biliary cirrhosis risk loci and targetable pathogenic pathways. *Nat. Commun.* **6**, 8019 (2015).
22. Ji, S.-G. et al. Genome-wide association study of primary sclerosing cholangitis identifies new risk loci and quantifies the genetic relationship with inflammatory bowel disease. *Nat. Genet.* **49**, 269–273 (2017).
23. Bentham, J. et al. Genetic association analyses implicate aberrant regulation of innate and adaptive immunity genes in the pathogenesis of systemic lupus erythematosus. *Nat. Genet.* **47**, 1457–1464 (2015).
24. Onengut-Gumuscu et al. Fine mapping of type 1 diabetes susceptibility loci and evidence for colocalization of causal variants with lymphoid gene enhancers. *Nat. Genet.* **47**, 381–386
