# Supplementary material for: A genome-wide association study of blood cell morphology identifies cellular proteins implicated in disease aetiology
Source: Nat Commun. 2023 Aug 18;14:5023. doi: 10.1038/s41467-023-40679-y (PMC10439125; doi:10.1038/s41467-023-40679-y)
Supplement: Supplementary file 2 — Description of Additional Supplementary Files [file 41467_2023_40679_MOESM2_ESM.pdf]

Description of Additional Supplementary Files

Akbari et al., A genome-wide association study of blood cell morphology identifies cellular proteins implicated in disease aetiology

Please refer to the Supplementary Information for the list of supplementary references.

File: Supplementary Data.xlsx

Supplementary Data Legends

Supplementary Data 1 | Demographic characteristics of 41,515 participants contributing to the ncCBC GWAS. a The distribution of sex, menopause status, smoking status and the season in which the haematology analyser measurement was made, in the sample of INTERVAL study participants that contributed to the genetic association analysis. The seasons are: Winter (December-February), Spring (March-May), Summer (June-August) and Autumn (September-November). **b** The means and standard deviations of age, body weight and body mass index in the sample of participants that contributed to the genetic association analysis.

Supplementary Data 2 | Descriptions of the ncCBC phenotypes. Each row corresponds to one of the 63 ncCBC phenotypes for which we performed a genetic association analysis. The first seven columns summarise properties of the phenotypes. In sequence they contain: the cell-type that the phenotype measures, the standard abbreviated name of the underlying trait, a long form name for the trait, the channel of the Sysmex XN instrument used to measure the phenotype, the units of measurement of the trait, a description of the trait, and the method by which the trait value is computed by the analyser (whether it is directly measured or computed from the measured values of other traits). The subsequent columns indicate the cCBC phenotype with which the phenotype has the greatest absolute Pearson correlation, the Pearson correlation (*r*) with that phenotype and the Pearson correlation between baseline and two-year follow up measurements of the phenotype. The penultimate and final columns respectively record the number of participants contributing to the GWAS of the phenotype and the estimate of the Genomic Control inflation factor (λ) from the GWAS summary statistics.

Supplementary Data 3 | Statistical summaries of distributions of ncCBC phenotypes. Each row corresponds to one of the 63 ncCBC phenotypes for which we performed a genetic association analysis. The first four columns have been described in the legend to Supplementary Data 2. The statistics in the subsequent columns are derived from the sample of individuals contributing to the GWAS that have complete data on sex, menopausal status, age and BMI. The fifth column reports the total variance of each technically adjusted trait (on the measured scale). The subsequent three triplets of columns report, respectively for males, pre-menopausal females and post-menopausal females, an estimate of the mean of the technically adjusted trait (linearly adjusted for age), the corresponding 95% confidence intervals and the variance of the technically adjusted trait. The adjustment for age is such that the estimate of the mean of the trait corresponds to the average age in the sample, which is 43.7 years. Then follows: an estimate of the per year effect of age on the mean of the trait linearly adjusted for sex and menopausal status and the corresponding standard error; the percentage of trait variance explained by multiple linear regression on sex, menopausal status and age; an estimate of the per kg m^{-2} effect of BMI on the mean of the trait linearly adjusted for sex, menopausal status and age and the corresponding standard error; the percentage of trait variance explained by multiple linear regression on sex, menopausal status, age and BMI.

Supplementary Data 4 | Summary of conditionally significant variant-trait associations. A table of the 2,172 conditionally significant variant-trait associations identified from GWAS of the 63 ncCBC traits listed in Supplementary Data 2. The 'Signal ID' column contains an identifier for the LD clump to which the associated variant belongs (any pair of variants with $r^2 > 0.8$ belong to the same clump). The physical coordinates of the associated variants are reported with respect to genome reference assembly GRCh37. The information recorded about each associated variant includes its minor allele frequency, its most serious VEP consequence, the corresponding gene symbol(s) and notes from a literature search on the function of the gene(s). Summary statistics are reported from univariable and multivariable analyses. For the univariable analyses (the primary GWAS), BOLT-LMM was used to fit mixed-effects models and perform quasi-likelihood score (χ^2) tests for additive allelic association. For the multivariable analyses, linear regression models were fit in which all variants exhibiting a conditionally significant association with the relevant trait were included as independent variables (Methods) and two sided *t*-tests were performed for additive allelic association. In all cases, $-\log_{10}$P-values are reported without adjustment for multiple comparisons. The 'FINEMAP Credible Set ID' column reports an identifier for the 95% posterior probability credible set to which the associated variant belongs, the total number of variants in that credible set is given in the final column.

Supplementary Data 5 | Summary of blood cell-type specific eQTL colocalisations with ncCBC association signals. A table of the 2,172 conditionally significant variant-trait associations, including rudimentary information about each variant (see legend to Supplementary Data 4), the HGNC assigned gene name corresponding to the transcript of any eQTL exhibiting colocalising association (see Methods), the corresponding blood cell-type (PLA=platelets, CD4=$CD4^+$ T-cells, CD8=$CD8^+$ T-cells, CD14=$CD14^+$ monocytes, CD15=$CD15^+$ neutrophils, and CD19=$CD19^+$ B-cells) and the posterior probability that the signals colocalise.

Supplementary Data 6 | BLUEPRINT expression and subcellular location. A table of the 2,172 conditionally significant variant-trait associations including rudimentary information about each variant (see legend to Supplementary Data 4), the cell-type of maximum expression of the gene corresponding to the most serious VEP consequence in the BLUEPRINT project RNA-seq data, the expression level of that gene measured in $\log_2$ fragments per kilobase of transcript per million mapped fragments ($\log_2$FPKM) in the trait-appropriate cell-type, the GO annotation for the subcellular location of the gene product and the localisation of the gene product in the relevant granule compartment in neutrophil cells (AG= azurophilic granules, CM=cell membrane, FG=ficolin-1 rich granules, SG=specific granules, SV=secretory vesicle).

Supplementary Data 7 | Summary of plasma protein concentration pQTL colocalisations with ncCBC association signals. A table of 2,172 conditionally significant variant-trait associations, including rudimentary information about each variant (see legend to Supplementary Data 4), the SOMAmer ID of any pQTL exhibiting colocalising association, an indication of whether any colocalising pQTL is cis or trans to the gene in question and the posterior probability that the signals colocalise[7].

Supplementary Data 8 | Summary of disease risk colocalisations with ncCBC association signals. A table of 2,172 conditionally significant variant-trait associations, including rudimentary information about each variant (see legend to Supplementary Data 4), the complex disease of any colocalising association and the posterior probability that the signals colocalise. The complex diseases considered are: allergic disease[8], Alzheimer's disease[9], asthma[10], coeliac disease[11,12], coronary artery disease[13], Crohn's disease[14,15], eczema[16], hayfever or rhinitis[8], inflammatory bowel disease[14,15], multiple sclerosis[17,18,19], primary biliary cirrhosis[20,21], primary sclerosing cholangitis[22], systemic lupus erythematosus[23], type 1 diabetes[24] and ulcerative colitis[14]. The numbers in square brackets after the complex disease name refer to the supplementary references and indicate the study from which the disease summary statistics where taken.
