# Supplementary material for: A genome-wide association study of blood cell morphology identifies cellular proteins implicated in disease aetiology
Source: Nat Commun. 2023 Aug 18;14:5023. doi: 10.1038/s41467-023-40679-y (PMC10439125; doi:10.1038/s41467-023-40679-y)
Supplement: Supplementary file 4 — Reporting Summary [file 41467_2023_40679_MOESM4_ESM.pdf]

nature portfolio

Corresponding author(s): William J. Astle

Last updated by author(s): 8th July 2023

Reporting Summary

Nature Portfolio wishes to improve the reproducibility of the work that we publish. This form provides structure for consistency and transparency in reporting. For further information on Nature Portfolio policies, see our Editorial Policies and the Editorial Policy Checklist.

Statistics

For all statistical analyses, confirm that the following items are present in the figure legend, table legend, main text, or Methods section.

n/a	Confirmed	
☐	☒	The exact sample size (*n*) for each experimental group/condition, given as a discrete number and unit of measurement
☐	☒	A statement on whether measurements were taken from distinct samples or whether the same sample was measured repeatedly
☐	☒	The statistical test(s) used AND whether they are one- or two-sided<br>*Only common tests should be described solely by name; describe more complex techniques in the Methods section.*
☐	☒	A description of all covariates tested
☐	☒	A description of any assumptions or corrections, such as tests of normality and adjustment for multiple comparisons
☐	☒	A full description of the statistical parameters including central tendency (e.g. means) or other basic estimates (e.g. regression coefficient) AND variation (e.g. standard deviation) or associated estimates of uncertainty (e.g. confidence intervals)
☐	☒	For null hypothesis testing, the test statistic (e.g. *F*, *t*, *r*) with confidence intervals, effect sizes, degrees of freedom and *P* value noted<br>*Give P values as exact values whenever suitable.*
☐	☒	For Bayesian analysis, information on the choice of priors and Markov chain Monte Carlo settings
☒	☐	For hierarchical and complex designs, identification of the appropriate level for tests and full reporting of outcomes
☐	☒	Estimates of effect sizes (e.g. Cohen's *d*, Pearson's *r*), indicating how they were calculated

Our web collection on statistics for biologists contains articles on many of the points above.

Software and code

Policy information about availability of computer code

Data collection	The Sysmex XN-1000 haematology analyser software was used to collect data from the Sysmex instrument.
Data analysis	Our research made use of the following R packages from CRAN: bit64 0.8-2, data.table 1, doMC 1.1.0, dplyr 0.1, ellipse 0.0.1, extrafont 0.9, forcats 0.1.0, foreach 1.2.0-1, GGally 0.1, ggplot2 0.5, ggrepel 0.3, ggthemes 1.3.1, grid 0.7-4, Hmisc 2.0-0, htmlwidgets 0.3.2, jsonlite 0.9.0, lattice 0.2-3, lubridate 0.1, MASS 7.3-0, mgcv 0.1-1, openxlsx 1.0.3, plotly 2.0.2, plyr 0.1, qqman 0.1.0, RColorBrewer 0.1-1, RcppEigen 0.1.1, reshape 0.4, reshape2 1, rgl 0.64-10, scales 0.1.0, scatterD3 0.1.1, seqminer 0.1, stringr 0.1.10, survival 2.6, svglite 1.0.0, tidyr 0.1, UpSetR 0.0.4 and the following R packages from Bioconductor: BiocManager 3.10, biomaRt 2.42.1, clusterProfiler 3.14.3, GenomicRanges 1.38.0, limma 3.42.2, qvalue 2.18.0, rhdf5 2.30.1 and the R packages g-chromVAR, gwas-pw 0.21. We also used Affymetrix Power Tools 1.18.0, BOLT-LMM 2.3.1, FINEMAP 1.3.1, IDEAS, LD Score 1.0.0, PLINK 1.90b3l, SHAPEIT3, Variant Effect Predictor 98.3, qctool 2.0.6. Our R code for the genetic association analysis is available in the git repository https://github.com/ParsaAkbari/UKBB500K-Conditional-Analysis.

For manuscripts utilizing custom algorithms or software that are central to the research but not yet described in published literature, software must be made available to editors and reviewers. We strongly encourage code deposition in a community repository (e.g. GitHub). See the Nature Portfolio guidelines for submitting code & software for further information.

Data

Policy information about availability of data

All manuscripts must include a data availability statement. This statement should provide the following information, where applicable:

- Accession codes, unique identifiers, or web links for publicly available datasets
- A description of any restrictions on data availability
- For clinical datasets or third party data, please ensure that the statement adheres to our policy

For ethical and legal reasons access to INTERVAL data are subject to controls. Bona fide scientists can seek access to relevant de-identified individual participant data — including genetic, haematology analyser and proteomic data — and a copy of the trial's data dictionary by applying to the INTERVAL Data Access Committee using the email address helpdesk@intervalstudy.org.uk. The INTERVAL Data Access Committee (supplemented, when required, by expertise from additional external scientists) meets several times a year to review applications according to the usual academic criteria of scientific validity and feasibility. Following approval by the INTERVAL Data Access Committee, a material transfer or research collaboration agreement will be agreed and signed with the applicants. Applicants might be requested to provide reimbursement of data management or preparation costs, as the INTERVAL trial is no longer in receipt of funding. Applicants will be required to provide updates to the INTERVAL Data Access Committee on their use of the INTERVAL trial data, including provision of copies of any publications. Applicants will be required to adhere in publications with the INTERVAL trial's policy for acknowledgment of the trial's funders, stakeholders, and scientific or technical contributors. The GRCh37 genome reference build is available for download from https://grch37.ensembl.org/info/data/ftp/index.html. Genomewide summary statistics may be downloaded by anonymous ftp from ftp://ftp.sanger.ac.uk/pub/project/humgen/summary_statistics/sysmex_blood_cell_genetics. The data from Ulirsch et al. are available from https://github.com/caleblareau/ singlecell_bloodtraits/, from the Gene Expression Omnibus (GEO) under accession GSE119453 and from the Sequence Read Archive (SRA) under accession PRJNA491478. Other MK epigenetic data were generated by the BLUEPRINT project and are available in the EGA dataset EGAD00001001871.

Research involving human participants, their data, or biological material

Policy information about studies with human participants or human data. See also policy information about sex, gender (identity/presentation), and sexual orientation and race, ethnicity and racism.

Reporting on sex and gender	Where analyses have been stratified they have been stratified by biological sex (e.g. Figure 2, Supplementary Figures 1-3). Where adjustments have been made to reduce residual variation (e.g. the primary genetic association analysis) such adjustments are made by biological sex. Sex was determined genetically and by self-reporting. Any participants for whom these methods proved inconsistent were excluded.
Reporting on race, ethnicity, or other socially relevant groupings	No analyses were performed according by race or ethnicity. Genetic analyses were performed in the European ancestry participants of the INTERVAL study (determined from genetic data) in order to reduce the possibility of confounding by population structure and to allow integration with summary data from other European ancestry genetic studies.
Population characteristics	The study participants are healthy blood donors. A detailed description of the characteristics of the study sample is given in Di Angelantonio et al., The Lancet, 2017 (https://doi.org/10.1016/50140-6736(17)31928-1)
Recruitment	The recruitment protocol is described in detail in Moore et al., Trials 2014 (https://doi.org/10.1186/1745-6215-15-363).
Ethics oversight	The INTERVAL trial received ethics committee approval from the National Research Ethics Service Committee East of England -- Cambridge East (Research Ethics Committee (REC) reference 11/EE/0538)

Note that full information on the approval of the study protocol must also be provided in the manuscript.

Field-specific reporting

Please select the one below that is the best fit for your research. If you are not sure, read the appropriate sections before making your selection.

☒ Life sciences ☐ Behavioural & social sciences ☐ Ecological, evolutionary & environmental sciences

For a reference copy of the document with all sections, see nature.com/documents/nr-reporting-summary-flat.pdf

Life sciences study design

All studies must disclose on these points even when the disclosure is negative.

Sample size	The INTERVAL study was designed primarily as a randomised trial to assess the optimal time interval between blood donations. The power calculations for the trial are described in Moore et al. Trials 2014, 15:363 (http://www.trialsjournal.com/content/15/1/363). Although the power of the trial is not directly relevant to the present work, these calculations determined the size of the study sample. A power calculation for genetic association analysis, shows that a test for additive allelic association between a common variant (minor allele frequency=50%) and a normally distributed quantitative trait has 80% power to detect an effect size corresponding to 0.047 standard deviations of phenotypic variance at a critical value of α=8.31 x 10^{-9}. Previous genome wide association studies have shown that such genetic effect sizes are quite common for human complex quantitative traits.
Data exclusions	Individuals were excluded because of non-European ancestry, poor quality genotypes, discordant self-reported and genetically inferred sex,

Data exclusions	phenotyping more than 36 hours after venipuncture, missing (unmeasured) phenotype data and outlying phenotype data.
Replication	Replication was not possible because we are not aware of any cohorts of a similar size phenotyped by the Sysmex XN haematology analyser. However, full blood count trait associations identified in INTERVAL previously using the same analysis protocol have a high rate of replicability in other datasets (e.g. UK Biobank).
Randomization	There was no controlled randomisation because this is a genetic study. However, as a consequence of the approximate panmixia of the study population and Mendel's law of segregation each study participant can be considered to have been randomised naturally to a genotype at each variant tested.
Blinding	The investigators prepared the phenotype data without reference to the genotype data and were therefore effectively blinded to the 'intervention' (allocation of participants to genotype group at each variant).

Reporting for specific materials, systems and methods

We require information from authors about some types of materials, experimental systems and methods used in many studies. Here, indicate whether each material, system or method listed is relevant to your study. If you are not sure if a list item applies to your research, read the appropriate section before selecting a response.

Materials & experimental systems

n/a	Involved in the study	
☐	☒	Antibodies
☐	☒	Eukaryotic cell lines
☒	☐	Palaeontology and archaeology
☒	☐	Animals and other organisms
☒	☐	Clinical data
☒	☐	Dual use research of concern
☒	☐	Plants

Methods

n/a	Involved in the study	
☒	☐	ChIP-seq
☐	☒	Flow cytometry
☒	☐	MRI-based neuroimaging

Antibodies

Antibodies used	BD Pharmingen APC Mouse Anti-Human CD41a, Clone HIP8, cat 559777.<br>BD Pharmingen PE Mouse Anti-Human CD42b, Clone HIP1, cat 555473
Validation	https://www.bdbiosciences.com/content/bdb/paths/generate-tds-document.de.559777.pdf<br>https://www.bdbiosciences.com/content/bdb/paths/generate-tds-document.de.555473.pdf

Eukaryotic cell lines

Policy information about cell lines and Sex and Gender in Research

Cell line source(s)	HPSI1113i-qolg_3 HipSci cell line (Clolg3; https://www.hipsci.org/lines/#/lines/HPSI1113i-qolg_3)
Authentication	Authenticated by HipSci, we routinely genotype our cells and did not observe any major variation overtime in comparison to reference genotypes.
Mycoplasma contamination	We routinely screen for mycoplasma using the MycoAlerf" detection kit and discard any cells for which results suggests contamination.
Commonly misidentified lines<br>(See ICLAC register)	HPSI1113i-qolg_3 is not a commonly misidentified cell line.

Flow Cytometry

Plots

Confirm that:

☒ The axis labels state the marker and fluorochrome used (e.g. CD4-FITC).

☒ The axis scales are clearly visible. Include numbers along axes only for bottom left plot of group (a 'group' is an analysis of identical markers).

☒ All plots are contour plots with outliers or pseudocolor plots.

☒ A numerical value for number of cells or percentage (with statistics) is provided.

Methodology

Sample preparation	*Describe the sample preparation, detailing the biological source of the cells and any tissue processing steps used.*
Instrument	*Identify the instrument used for data collection, specifying make and model number.*
Software	*Describe the software used to collect and analyze the flow cytometry data. For custom code that has been deposited into a community repository, provide accession details.*
Cell population abundance	*Describe the abundance of the relevant cell populations within post-sort fractions, providing details on the purity of the samples and how it was determined.*
Gating strategy	*Describe the gating strategy used for all relevant experiments, specifying the preliminary FSC/SSC gates of the starting cell population, indicating where boundaries between "positive" and "negative" staining cell populations are defined.*

☒ Tick this box to confirm that a figure exemplifying the gating strategy is provided in the Supplementary Information.
